# Pain assessment for people with dementia: a systematic review of systematic reviews of pain assessment tools

**DOI:** 10.1186/1471-2318-14-138

**Published:** 2014-12-17

**Authors:** Valentina Lichtner, Dawn Dowding, Philip Esterhuizen, S José Closs, Andrew F Long, Anne Corbett, Michelle Briggs

**Affiliations:** School of Healthcare, University of Leeds, Leeds, UK; Columbia University School of Nursing, 617 West 168th Street, New York, NY 10032 USA; Center for Home Care Policy and Research, Visiting Nurse Service of New York, 5 Penn Plaza, New York, NY 10001 USA; Wolfson Centre for Age-Related Diseases, King’s College London, London, SE1 1UL UK; Institute of Health and Wellbeing, Leeds Beckett University, Leeds, UK

**Keywords:** Pain assessment, Pain scales, Aged, Dementia, Meta-review

## Abstract

**Background:**

There is evidence of under-detection and poor management of pain in patients with dementia, in both long-term and acute care. Accurate assessment of pain in people with dementia is challenging and pain assessment tools have received considerable attention over the years, with an increasing number of tools made available. Systematic reviews on the evidence of their validity and utility mostly compare different sets of tools. This review of systematic reviews analyses and summarises evidence concerning the psychometric properties and clinical utility of pain assessment tools in adults with dementia or cognitive impairment.

**Methods:**

We searched for systematic reviews of pain assessment tools providing evidence of reliability, validity and clinical utility. Two reviewers independently assessed each review and extracted data from them, with a third reviewer mediating when consensus was not reached. Analysis of the data was carried out collaboratively. The reviews were synthesised using a narrative synthesis approach.

**Results:**

We retrieved 441 potentially eligible reviews, 23 met the criteria for inclusion and 8 provided data for extraction. Each review evaluated between 8 and 13 tools, in aggregate providing evidence on a total of 28 tools. The quality of the reviews varied and the reporting often lacked sufficient methodological detail for quality assessment. The 28 tools appear to have been studied in a variety of settings and with varied types of patients. The reviews identified several methodological limitations across the original studies. The lack of a ‘gold standard’ significantly hinders the evaluation of tools’ validity. Most importantly, the samples were small providing limited evidence for use of any of the tools across settings or populations.

**Conclusions:**

There are a considerable number of pain assessment tools available for use with the elderly cognitive impaired population. However there is limited evidence about their reliability, validity and clinical utility. On the basis of this review no one tool can be recommended given the existing evidence.

**Electronic supplementary material:**

The online version of this article (doi:10.1186/1471-2318-14-138) contains supplementary material, which is available to authorized users.

## Background

Dementia affects an estimated 44.4 million people worldwide – a figure which is set to rise – and represents an important public health issue, costing an estimated US$604 billion each year [1]. Dementia presents a particular challenge for treatment and care due to the progressive cognitive and functional decline that are hallmarks of the condition. In particular, loss of language and the ability to communicate raises the risk of unmet need. A critical example of this issue is in the assessment of pain.

Pain is common in older adults, with up to one third of community-dwelling people over 60 experiencing regular pain and 50% of people over 80 regularly taking analgesics [2]. Although pain in dementia is difficult to assess, the literature agrees that at least 50% of people with dementia also regularly experience pain [3], which is predominantly, but not exclusively related to the musculoskeletal system. Osteoarthritis is very common in these individuals [4], and pain is also frequently caused by falls, pressure ulcers, infections and underlying neuropathy due to comorbidities [5].

In addition to significant distress and discomfort, untreated pain can also be a causative factor in key symptoms and quality of life indicators for people with dementia. Behavioural and psychological symptoms of dementia such as agitation and aggression often arise as a result of underlying pain, presenting a considerable challenge for treatment and care that may lead to institutionalisation or prescriptions of antipsychotic medications that carry serious safety concerns [6]. The accurate and timely assessment of pain is therefore of critical importance when considering the overall care for people with dementia. In general, due to the subjectivity of pain, self-report is considered to be the gold standard for pain assessment. While people with mild to moderate dementia are often able to report their pain verbally or use simple visual or numerical pain intensity assessment tools, these options are not feasible for use with people with later stage dementia in whom communication ability is severely impaired [7, 8]. As a result previous work has shown that pain is frequently under-detected and poorly managed in people with dementia, in both long-term and acute care [9–12].

In the absence of accurate self-report it has been necessary to develop observational tools to be used in both research and practice, based on the interpretation of behavioural cues as a proxy for the presence of pain. This approach has resulted in a proliferation in the number of pain assessment instruments developed to identify behavioural indicators of pain in people with dementia and other cognitive impairment. The most structured of these are predominantly based on guidance published by the American Geriatrics Society [13], which presents six domains for pain assessment in older adults. These include facial expression, negative vocalisation, body language, changes in activity patterns, changes in interpersonal interactions and mental status changes. However, the interpretation of many of these behaviours is complex when applied to dementia due to considerable overlap with other common behavioural symptoms or cognitive deficits which may confound an assessment, manifesting from boredom, hunger, anxiety, depression or disorientation [14]. This increases the complexity of identifying the presence of pain accurately in patients with dementia and raises questions about the validity of existing instruments. The psychometric and discriminative properties and the clinical utility of currently available instruments are as yet unclear. As a result there is no clear guidance for clinicians and care staff on the effective assessment of pain, nor how this should inform treatment and care decision-making. A large number of systematic reviews have been published which analyse the relative value and strength of evidence of existing pain tools. There is a need for guidance on the best evidence available and for an overall comprehensive synthesis.

This meta-review forms part of a larger programme of research focusing on developing decision support interventions to assist with the assessment and management of pain in patients with dementia in an acute care setting. A key aspect of this work is to identify existing assessment tools with validation data pertaining to the treatment of people with dementia. This meta-review presents a thorough synthesis of current systematic review literature concerning the psychometric properties and clinical utility of pain assessment tools for the assessment of pain in adults with dementia. It provides a detailed picture of the state of the field in the complex task of assessing pain.

## Methods

For ease of reference, in this paper we refer to our systematic review of systematic reviews as a *meta-review*; we call the systematic reviews considered for inclusion in the meta-review *reviews* and refer to publications included in the reviews as *studies*; we use the term *records* to refer to the bibliographic data of publications of reviews (for the most part retrieved thought online database searches); the terms (pain assessment) *scales*, *tools* and *instruments* are used interchangeably. The process of the meta-review followed guidance from The Cochrane Collaboration [15] and the Joanna Briggs Institute [16]. In undertaking this meta-review, our review questions were the following:

1. Which tools are available to assess pain in adults with dementia?
2. In which settings are they used and with what patient populations?
3. What are their reliability, validity and clinical utility?

### Criteria for considering reviews for inclusion

Definitions of criteria for inclusion of reviews in the meta-review followed an adapted SPICE structure (Setting, Population, Intervention, Comparison, method of Evaluation) [16] (Table 1). We included systematic reviews of pain assessment tools involving adults with dementia or with cognitive impairment. Dementia and cognitive impairment were defined according to the US National Library of Medicine Medical Subject Heading (MeSH) vocabulary. Dementia was defined as “an acquired organic mental disorder with loss of intellectual abilities of sufficient severity to interfere with social or occupational functioning […]” [17]. The Dementia MeSH term covers more specific subheadings such as Alzheimer Disease or Vascular Dementia. Cognition Disorder was defined as: “Disturbances in the mental process related to thinking, reasoning, and judgment” [18] (distinct from, not including, Delirium). We did not include Learning Disorders, defined as: “Conditions characterized by a significant discrepancy between an individual’s perceived level of intellect and their ability to acquire new language and other cognitive skills. […]” [19]. Examples of learning disorders of this type are dyslexia, dyscalculia, and dysgraphia.

**Table 1:** **Inclusion and exclusion criteria**

	Criteria	Definitions
Setting	Reviews pertaining to any setting	*Settings* are for example acute hospitals, nursing homes, community settings.
Patient population	Reviews of studies limited to adult dementia patients or adults with cognitive impairment.	*Dementia* defined as “an acquired organic mental disorder with loss of intellectual abilities of sufficient severity to interfere with social or occupational functioning. The dysfunction is multifaceted […]”
	All stages of dementia in adults considered (e.g. mild, severe).	*Cognitive impairment* defined as Cognition Disorder: “Disturbances in the mental process related to thinking, reasoning, and judgment”. Does not include Learning Disorders. (Source: MeSH vocabulary -http://www.nlm.nih.gov/mesh/)
Intervention	Reviews of studies of the assessment of pain and of pain assessment tools. Reviews that include management of pain considered if they also cover assessment of pain.	Pain assessment as defined by IASP “entails a comprehensive evaluation of the patient’s pain, symptoms, functional status, and clinical history […]”.
	All forms of pain considered (e,g. acute pain, persistent), without distinction on location of pain (e.g. abdominal pain).	“[…] The assessment process is essentially a dialogue between the patient and the health care provider that addresses the nature, location and extent of the pain, and looks at the patient’s daily life, and concludes with the pharmaceutical and nonpharmaceutical treatment options available to manage it” [20]. Evaluation tools may be used in this process: “To varying degrees, these tools attempt to locate and quantify the severity and duration of the patient’s subjective pain experience […]” [20].
	Reviews of studies of pain assessment included, irrespective of the outcomes of the assessment (e.g. patients being in pain or not).	
Evaluation (method of)	Systematic reviews only to be included	Definition of systematic review:
		1. Review carried out systematically – i.e. publication that makes explicit the authors’ intention to review or summarise the literature (e.g. with review, overview, or meta-analysis in the title or in a section heading) [21].
		2. Satisfying the following criteria [22]:
		- Clear set of objectives: explicit and clear research question
		- Reproducible methodology: the paper clearly explains how the evidence was retrieved, including sources and search strategy and the inclusion (and exclusion) criteria
		- Assessment of validity of the findings (e.g. assessment of risk of bias)
		- Systematic presentation and synthesis of findings beyond those provided by single studies.
Additional criteria	Reviews to be included only if with data and/or assessment of reliability and/or validity and/or clinical utility	Reliability: “the degree to which the measurement is free from measurement error” [23].
		Validity: “the degree to which the [instrument] measures the construct(s) it purports to measure” [23].
	Inclusion limited to English language	Clinical utility: “the usefulness of the measure for decision making”, i.e. to inform further action, such as the administration of analgesics [24].

The definition of criteria for inclusion of potentially relevant reviews follows an adapted SPICE structure (Setting, Population, Intervention, Comparison, method of Evaluation) [16]. All criteria must be met for reviews to be included.

We included reviews regardless of setting (e.g. acute, or nursing/care homes), type, location or intensity of pain (e.g. acute pain, persistent), and outcomes of the pain assessment (e.g. patients being in pain or not). Reviews were included if they provided psychometric data for the pain assessment tools and were available in English. We excluded publications, such as narrative reviews or case reports, which did not provide psychometric data or were not categorized as systematic reviews [22] (see Table 1 for our systematic review definition).

### Search methods for identification of reviews

The following databases were searched (details provided in Table 2): Medline, All EBM Reviews (including Cochrane DSR, ACP Journal Club, DARE, CCTR, CMR, HTA, and NHSEED), Embase, PsycINFO, and CINHAL; the searches were carried out all on the same date (12 March 2013). Additional searches included the Joanna Briggs Institute (JBI) Library (The JBI Database of Systematic Reviews and Implementation Reports) and the Centre for Reviews and Dissemination database. Further data was retrieved through reference chaining. No grey literature was sought.

**Table 2:** **Literature search: databases and details of numbers of records retrieved**

Date/time	Database	# records retrieved (including duplicates)	# records retrieved (excluding duplicates)
12 March 2013 11:12	MEDLINE (specifically: Ovid MEDLINE 1946 to February Week 4 2013)	209	208
12 March 2013 11:22	Ovid MEDLINE In-Process & Other Non-Indexed Citations March 11, 2013	0	0
12 March 2013 11:14	All EBM Reviews - Cochrane DSR, ACP Journal Club, DARE, CCTR, CMR, HTA, and NHSEED	68	67
12 March 2013 12:05	Embase (1996 to 2013 Week 10)	74	73
12 March 2013 12:22	PsycINFO (1806 to March Week 1 2013)	68	0
12 March 2013 13:40	CINHAL	78	0
12 March 2013 14:15	The Joanna Briggs Institute (JBI) Library - The JBI Database of Systematic Reviews and Implementation Reports	6	0
12 March 2013 14:49	Centre for Reviews and Dissemination database	5	0
	Total:	508	441 (67 duplicates – i.e. 11 records retrieved in 2 or more databases)

A summary of the databases searched, the number of bibliographic records retrieved for each database, including and excluding duplicates. Duplicates consists of records retrieved in two or more databases.

The search strategy used a combination of text words and established indexing terms such as Medical Subject Headings (Table 3). The search was structured by the relevant SPICE concepts. Search terms were identified by comparing published search strategies adopted by reviews in similar areas (such as [21, 25]), or on the subject of pain or pain management tools, not specifically for the same patient population [26], using the search strategy for retrieving reviews outlined by Montori et al. [27]. Detailed search strategies were optimised for each electronic database searched (see Additional file 1).

**Table 3:** **Search strategy**

SPICE categories	Search terms*
Patient population: adults with dementia or cognitive impairment	1. Dementia.mp.
	2. Alzheimer.mp
	3. exp Dementia/
	4. exp Alzheimer Disease/
	5. 1 or 2 or 3 or 4
	6. exp Cognition Disorders/
	7. Cognitive impairment.mp.
	8. Cognitive function*.mp.
	9. exp mental retardation/
	10. 6 or 7 or 8 or 9
	11. 5 or 10
Intervention: pain assessment	12. (Assess$ adj5 pain).mp.
	13. (Measur$ adj5 pain).mp.
	14. (Scale$ adj5 pain).mp.
	15. (Rating adj5 pain).mp.
	16. exp Pain Measurement/
	17. exp Pain/di
	18. *Pain Measurement/mt
	19. exp *Pain Measurement/
	20. (Pain adj3 tool$).mp.
	21. 12 or 13 or 14 or 15 or 16 or 17 or 18 or 19 or 20
	22. 11 and 21
Limited to study design: reviews	23. meta-analysis.mp.
	24. meta-analysis.pt.
	25. review.pt.
	26. search:.tw.
	27. 23 or 24 or 25 or 26
	28. 22 and 27

*mp, pt, tw are abbreviations identifying specific fields in the OVID™ MEDLINE database – e.g. mp = title, abstract, original title, name of substance word, subject heading word, keyword heading word, protocol supplementary concept, rare disease supplementary concept, unique identifier. The / after each term is the OVID™ MEDLINE convention for a MESH term; the ‘exp’ abbreviation refers to the automatic expansion of a MeSH term to its sub-headings.

### Selection of reviews

**Figure 1:**
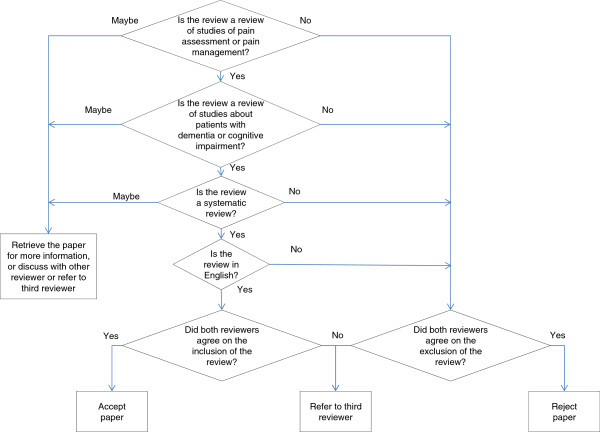
**Algorithm for inclusion/exclusion of reviews.** Screening algorithm (based on inclusion criteria as per Table 1). Each decision point has the three options for promoting records to the next stage of the winnowing process: ‘yes’ (inclusion), ‘no’ (exclusion), ‘maybe’. Depending on the stage of the reviewing process (on title and abstract only, or full text), the latter option may require retrieving the full text, discussion between reviewers, and/or referral to a third reviewer.

Four reviewers (DD, MB, VL, PE) screened all search results, initially on the basis of title and abstract and then the full text of potentially eligible papers (Figure 1). The results of the search were divided into two sets among the reviewers, so that each review was assessed by two people independently. When consensus could not be reached, the reviews were referred to a third party (SJC) (details of this process available in Additional files 2 and 3).

### Assessment of methodological quality of included reviews

The PRISMA guidance on systematic reviews explains how, in carrying out a systematic review, it is important “to distinguish between quality and risk of bias and to focus on evaluating and reporting the latter” [22]. The authors encourage the reviewers “to think ahead carefully about what risks of bias (methodological and clinical) may have a bearing on the results of their systematic reviews” [22]. In our meta-review, the risk of bias may reside in each review considered for inclusion, as well as in the original studies that comprise that review. We did not access the studies to be able to accurately judge their quality or risk of bias. In terms of each review, we assessed risk of bias in terms of how the review was conducted and the criteria applied for inclusion/exclusion. Critical appraisal was carried out by two independent reviewers, using the AMSTAR systematic review critical appraisal tool [28]. Critical appraisal and evaluation of potential bias was carried out at the time of data extraction, after screening was completed on the basis of the inclusion criteria.

### Data extraction and management

Data were extracted by two reviewers independently using a set of data extraction forms which was developed for the meta-review: 1) the AMSTAR checklist [28], 2) two forms for data about the reviews and 3) one form for data about the tools (Additional file 4). The latter included a field for data extraction on the user-centredness of the tools, informed by Dixon and Long’s work on the development of health status instruments [29]. The data extraction forms were both paper-based and built into a MS Access database. At the time of data extraction, the reviews eligible for inclusion were screened further on the basis of availability of psychometric data of tools. At this point, we found that some of the reviews initially identified as being eligible for inclusion in the meta-review did not provide psychometric data of tools and were subsequently excluded (this is discussed in detail in the results section). Data about the characteristics of the tool (e.g. tool design and instructions for use) were extracted from the reviews; we did not search for, nor retrieve, the original tools. The reviews were synthesised using a narrative synthesis approach.

## Results

**Figure 2:**
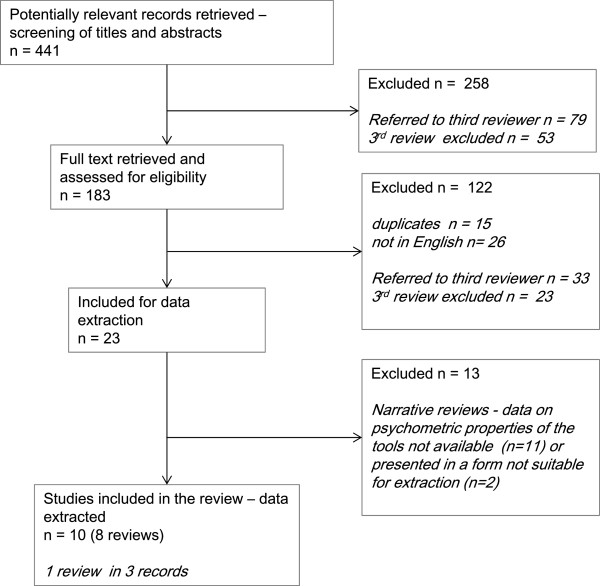
**Flow chart of retrieved sources and screening process.** Overview of the review process and number of retrieved, included and excluded records.

The search retrieved 441 potentially eligible unique records. After screening titles and abstracts, and further removing duplicates, we obtained the full text of 183 records and assessed these for eligibility. We identified 23 reviews as being potentially eligible for inclusion, of which 13 were excluded as they did not provide data on the psychometric properties of the tools. The remaining set included 10 records reporting data from eight reviews (Schofield et al. [30] review was reported in three separate studies [30–32], we have combined the results of this) (Figure 2). Table 4 provides details of the eight included reviews and Table 5 details of the 13 excluded reviews.

**Table 4:** **List of included reviews**

Review ID	Review	Notes on inclusion
[25]	Corbett A, Husebo B, Malcangio M, Staniland A, Cohen-Mansfield J, Aarsland D, et al. Assessment and treatment of pain in people with dementia. Nature Reviews Neurology. 2012; 8(5):264–74.	Meets inclusion criteria – data available on supplementary table
[33]	Herr K, Bjoro K, Decker S. Tools for Assessment of Pain in Nonverbal Older Adults with Dementia: A State-of-the-Science Review. Journal of Pain and Symptom Management. 2006; 31(2):170–92.	Meets inclusion criteria and psychometric data provided
[34]	Smith M. Pain assessment in nonverbal older adults with advanced dementia.Perspectives in Psychiatric Care. 2005; 41(3):99–113.	Meets inclusion criteria and psychometric data provided
[35]	Qi NS, Brammer JD, Creedy DK. The psychometric properties, feasibility and utility of behavioural-observation methods in pain assessment of cognitively impaired elderly people in acute and long-term care: A systematic review. 2012. 2012; 10(17).	Meets inclusion criteria and psychometric data provided
[36]	Juyoung P, Castellanos-Brown K, Belcher J. A Review of Observational Pain Scales in Nonverbal Elderly With Cognitive Impairments. Research on Social Work Practice. 2010; 20(6):651–64.	Meets inclusion criteria and psychometric data provided
[30]	Schofield P, Clarke A, Faulkner M, Ryan T, Dunham M, Howarth A. Assessment of pain in adults with cognitive impairment: A review of the tools. International Journal on Disability and Human Development. 2005; 4(2):59–66.	Include [30–32] as 1 review with 3 publications. This [30] is the primary reference; meets inclusion criteria and psychometric data provided
[24]	van Herk R, van Dijk M, Baar FP, Tibboel D, de Wit R. Observation Scales for Pain Assessment in Older Adults with Cognitive Impairments or Communication Difficulties. Nursing Research. 2007; 56(1):34-43	Meets inclusion criteria and psychometric data provided
[21]	Zwakhalen SMG, Hamers JPH, Abu-Saad HH, Berger MPF. Pain in elderly people with severe dementia: a systematic review of behavioural pain assessment tools. BMC Geriatrics. 2006; 6(3).	Meets inclusion criteria and psychometric data provided

Bibliographic information of the reviews included in this meta-review. For ease of referencing these are also included in the References, with the corresponding unique identifier.

**Table 5:** **List of excluded reviews**

Review ID	Review	Reason for exclusion
[37]	Thuathail AN, Welford C. Pain assessment tools for older people with cognitive impairment. Nursing Standard. 2011; 26(6):39–46.	Narrative review. The review covers several tools, with very little information of each. Comparison is difficult as no summary table is provided.
[38]	Lord B. Paramedic assessment of pain in the cognitively impaired adult patient. BMC Emergency Medicine. 2009; 9:20.	Narrative review. No data on psychometric properties.
[39]	While C, Jocelyn A. Observational pain assessment scales for people with dementia: a review. British Journal of Community Nursing. 2009; 14(10):438, 9–42.	A review of reviews. No data on psychometric properties of the tools.
[40]	Rutledge DN, Donaldson NE, Pravikoff DS. Update. Pain assessment and documentation. Special populations of adults. Online Journal of Clinical Innovations. 2002; 5(2):1–49.	Broad review of pain in a variety of different patient groups - of which adults with cognitive impairment a subset. No data on psychometric properties of the tools to extract.
[41]	Rutledge DR, Donaldson NE. Pain assessment and documentation. Part II - special populations of adults. Online Journal of Clinical Innovations. 1998; 1(6):1–29.	No data on psychometric properties of the tools. Review updated in [40].
[42]	Andrade DCd, Faria JWVd, Caramelli P, Alvarenga L, Galhardoni R, Siqueira SRD, et al. The assessment and management of pain in the demented and non-demented elderly patient. Arquivos de Neuro-Psiquiatria. 2011; 69(2B):387–94.	Narrative review, including physiology, assessment and management of pain. No data on psychometric properties of the tools.
[43]	Scherder E, Oosterman J, Swaab D, Herr K, Ooms M, Ribbe M, et al. Recent developments in pain in dementia. BMJ. 2005; 330(7489):461–4.	Narrative review, no data on psychometric properties of tools.
[44]	Lobbezoo F, Weijenberg RAF, Scherder EJA. Topical review: orofacial pain in dementia patients. A diagnostic challenge. Journal of Orofacial Pain. 2011; 25(1):6–14.	Narrative review. No data on psychometric properties of the tools. Focus: orofacial pain.
[45]	Herr K, Bursch H, Ersek M, Miller LL, Swafford K. Use of pain-behavioral assessment tools in the nursing home: expert consensus recommendations for practice. Journal of Gerontological Nursing. 2010; 36(3):18–29;	Expert reviewers were asked to rate each tool and provide a score; no data on psychometric properties of tools. The way in which the data are presented makes it unusable for our purposes.
[46]	McAuliffe L, Nay R, O’Donnell M, Fetherstonhaugh D. Pain assessment in older people with dementia: literature review. Journal of Advanced Nursing. 2009; 65(1):2–10.	A narrative review with no data on tools. Focus: barriers to successful pain assessment.
[47]	Miller LL, Talerico KA. Pain in older adults. Annual Review of Nursing Research. 2002; 20:63–88.	Narrative review. No data on psychometric properties of the tools.
[48]	Helfand M, Freeman M. Assessment and management of acute pain in adult medical inpatients: A systematic review. Pain Medicine. 2009; 10(7):1183–99.	Narrative review. No data on psychometric properties of the tools.
[49]	Stolee P, Hillier LM, Esbaugh J, Bol N, McKellar L, Gauthier N. Instruments for the assessment of pain in older persons with cognitive impairment. Journal of the American Geriatrics Society. 2005;53(2):319–26.	30 tools reviewed comparatively in overview tables. Reliability and validity data reported but in a form unusable for our purposes.

Bibliographic information of the reviews considered eligible for inclusion in this meta-review, but excluded from the data extraction process. For ease of referencing these are also included in the References, with the corresponding unique identifier.

The findings are structured as follows. First, we briefly summarise the reviews considered at the time of data extraction but excluded for lack of data on psychometric properties of the tools. We then describe the reviews included in our analysis – their methods and our quality assessment. Third, we describe the findings of these reviews, i.e. the pain assessment tools - characteristics, psychometric properties, feasibility of use and clinical utility. We conclude with the reviews’ overall assessment of these tools.

### Description of excluded reviews

Thirteen of the 23 reviews were excluded because they did not provide suitable data for extraction (these data were absent or not reported in a format suitable for extraction). Several of these were narrative reviews. They varied in length and details of reporting. Tools were analysed in the broader context of, for example, pain physiology, pain prevalence, patients’ attitudes and beliefs about pain. For some the focus were the processes of pain assessment and/or pain management/interventions in the elderly population, including with (but not always limited to) persons with dementia (e.g. in [40–42, 47]), and/or in specific types of pain ([44]- orofacial pain). One review [46] focused on the barriers to successful pain assessment (of which non-use of assessment tools is one).

Two reviews were updated by, or related to, a later review by the same authors ([40] is an update of [41]– both excluded; [45] is related to [33] - the latter included in our study - and aimed at reviewing the methods of the previous study).

Two reviews [38, 39] are review of reviews. They are very specific in their aims, i.e. to identify pain assessment tools for cognitively impaired adults recommended for use by paramedics [38] and district nurses [39]. They identified two and four reviews respectively, which were amongst those we also retrieved. Thus, the reasons for exclusion in their case were: review of reviews (overview, rather than a systematic review), no data for extraction, and on the grounds of repetition.

Two other reviews [45, 49] did report and discuss psychometric data, but not in a form suitable for this meta review. Their choice of reporting may suggest authors’ methodological concerns for the comparability and presentation of the data in quantitative form, given the heterogeneity of the studies and their methods.

### Description of included reviews

Each review included in this meta-review comprised between eight and 13 tools (Table 6, see also Additional file 5). The most frequently reviewed tools included: the Abbey Pain Scale, NOPPAIN, PACSLAC, PADE, CNPI and PAINAD (Table 7; abbreviations of name of tools explained in the List of abbreviations used). Reviews searched the literature across a variety of date ranges, from 1980 to 2010. The number of individual studies included in each review varied from nine [30] to 29 [21], although the number of included studies in some reviews was ambiguous (Table 6). The reasons for this ambiguity are twofold. First, the number of studies included in each review is different for each tool, thus making it difficult to aggregate in one number (‘number of included studies’). Second, the studies included in each review were found each to have reported one or more studies aimed at evaluating a tool. Thus, a number of included studies of ‘1’ may actually refer to a larger number of studies conducted.

**Table 6:** **Characteristics of included reviews: data sources and number of studies**

Review ID	Number of included studies*	Search dates	Databases searched	Number of tools reviewed	Country of origin
[25]	18	No data	PubMed, MEDLINE, EMBASE	12	UK
[33]	No data	1990 - July 2004	MEDLINE, CINAHL, PsycInfo and Health, and Psychosocial Instruments + electronic database of the National Guideline Clearinghouse + pain and gerontological conferences + personal reference databases of the authors.	10	USA
[34]	No data	No data	MEDLINE, CINAHL, PubMed, EMB Reviews	8	USA
[35]	23	1990-2010	CINAHAL, MEDLINE, Scopus, PsycInfo, ScienceDirect, Wiley-Interscience, Mosby’s Nursing Consult, Web of Science, ProQuest + reference lists to identify additional studies. Unpublished studies and grey literature not included in review	10	Singapore and Australia
[36]	21	1990 - 2007	MEDLINE, CINAHL, PsycInfo, Sociological Abstracts, Social Sciences Abstracts, and Ageline	11	USA
[30]	9	1994-2004	AHMED, CINAHL, MEDLINE, EMBASE, Science Citation Index, Psychlit, Ageinfo, Anchor housing, Index for thesis, Steinberg	9	UK
[24]	No data	1980-2005	PubMed, MEDLINE, PsycInfo, Cinahl, PiCarta	13	The Netherlands
[21]	29	1988 to January 2005	MEDLINE, PsycInfo, CINAHL + screening citations and references + Unpublished manuscripts were collected by approaching colleagues working in the field of pain among the elderly + abstract books of the 7th, 8th, 9th and 10th International Association for the Study of Pain World Congresses screened for relevant publications	12	Lebanon and Netherlands

Overview of the scope of the retrieval strategies (sources and periods) for studies of pain assessment tools, used in the reviews included in our meta-review. Information was missing for four of the reviews (no data available for extraction). * The number of included studies is possibly approximate. The reasons are twofold: 1) the number of included studies in each review is different for each tool and hard to aggregate in one number; 2) the studies included in each review may have reported one or more studies aimed at evaluating a tool – i.e. a number of included studies of ‘1’ may actually refer to a larger number of studies conducted.

**Table 7:** **Summary of how often the tools appear in the reviews (in alphabetical order)**

Name of tool	Number of reviews they appear in	Review IDs
Abbey pain scale	7	[21, 24, 25, 30, 33, 35, 36]
ADD Protocol	5	[24, 30, 33, 34, 36]
Behavior checklist	2	[24, 36]
CNPI	8	[21, 24, 25, 30, 33–36]
Comfort checklist	1	[34]
CPAT	1	[35]
Doloplus-2	6	[21, 24, 25, 30, 33, 35]
DS-DAT	6	[24, 25, 30, 33, 34, 36]
ECPA	1	[21]
ECS	1	[21]
EPCA-2	1	[25]
FACS	1	[24]
FLACC	1	[33]
Mahoney pain scale	1	[35]
MOBID	3	[25, 35, 36]
NOPPAIN	7	[21, 24, 25, 30, 33, 35, 36]
Observational pain behaviour tool	2	[21, 34]
PACSLAC	7	[21, 24, 25, 30, 33, 35, 36]
PADE	7	[21, 24, 25, 30, 33, 34, 36]
Pain assessment scale for use with cognitively impaired adults	1	[21]
PAINAD	8	[21, 24, 25, 30, 33–36]
PAINE	1	[25]
PATCOA	2	[24, 36]
PBM	1	[24]
PPI	1	[25]
PPQ	1	[34]
RaPID	1	[21]
REPOS	1	[35]

The reviews aimed to summarise the available evidence by means of a comprehensive overview (Additional file 5). Three reviews [33, 35, 36] also explicitly aimed at an evaluation of the evidence – i.e. to critically evaluate the existing tools, or to identify key components and analyse the reported psychometric properties of tools. Two reviews [21, 33] reported a systematic method for evaluation of the tools.

Not all reviews made explicit their assessment of the quality of the studies or risk of bias, or assessment of the scales considered. When this was done, the reviews highlighted the methodological limitations of both studies and scales (Additional file 6). For example, in one review [21] the overall assessment was “generally moderate”, with 11 points being the highest score out of the 20 point evaluation scale applied, and only four of the 12 tools examined reaching this score (these were Doloplus-2, ECPA, PACSLAC, PAINAD). The heterogeneity of study designs and/or inconsistencies made aggregation of findings in the reviews difficult and/or methodologically inappropriate. For example Zwakhalen et al. [21] stressed the “Considerable heterogeneity in terms of design (retrospective vs. prospective), method (pain in vivo vs. observational methods), research population (different types of dementia, different levels of impairment, different settings) and conceptualisation of pain, making their results hard to compare”.

### Quality of included reviews

We assessed the quality of the systematic reviews through the use of the AMSTAR questionnaire (original questionnaire adapted to a binary scoring: 1 if item is present, 0 if unclear, absent or not applicable) (Table 8). The mean score was about 4.9, in the range from 1 [34] to 10 [35].

**Table 8:** **Summary of quality of systematic reviews**

Review ID	Q1 - Was an ‘a priori’ design provided?	Q2 - Was there duplicate study selection and data extraction?	Q3 - Was a comprehensive literature search performed?	Q4 - Was the status of publication (i.e. grey literature) used as an inclusion criterion?	Q5 - Was a list of studies (included and excluded) provided?	Q6 - Were the characteristics of the included studies provided?	Q7 - Was the scientific quality of the included studies assessed and documented?	Q8 - Was the scientific quality of the included studies used appropriately in formulating conclusions?	Q9 - Were the methods used to combine the findings of studies appropriate?	Q10 - Was the likelihood of publication bias assessed?	Q11 - Was the conflict of interest stated?	Total score
[25]	1	0	1	0	1	1	0	1	1	0	1	7
[33]	1	0	1	1	0	1	1	0	1	0	0	6
[34]	0	0	0	0	0	1	0	0	0	0	0	1
[35]	1	1	1	0	1	1	1	1	1	1	1	10
[36]	1	0	1	0	1	1	1	0	0	1	0	5
[30]	1	0	1	0	0	0	0	1	0	0	0	3
[24]	1	0	1	0	0	0	0	0	0	0	0	2
[21]	1	0	0	1	0	0	0	1	0	0	1	4
											**Mean**	4.875

Assessment of the quality of the included reviews by application of the AMSTAR Questionnaire [28], adapted to a binary scoring – items scored 1 if present, 0 if unclear, absent or not applicable. The questionnaire is composed of 11 closed questions, with possible answers: Yes, No, Can’t Answer and Not Applicable. The quality assessment we carried out relied on the information reported in the review - we did not contact the authors of the reviews to gather information which was missing or ambiguous in their publication. As a consequence, it may be possible that the authors had performed, for example, a comprehensive literature search for their review, but they did not report this in sufficient detail in their publication. This also caused uncertainty and ambiguity between the No and Can’t Answer options, with blurred boundaries between the two. In addition, the AMSTAR questionnaire presents some double-barrelled questions (Q2, Q5, Q7) and we scored the item as present (a score of 1) only when both items in the question were answered positively. So, for example, only when both the lists of included and excluded studies were provided (Q5) a score of 1 would be awarded to the review.

Most (6/8 reviews) presented an a priori design and a comprehensive literature search (Q1; Q3). However, in general, the reporting lacked in detail. For example, as shown in Table 8, the list of included/excluded studies was provided in only three reviews [25, 35, 36]; the explicit involvement of two or more independent reviewers (Q2) was reported in only one review [35]; only three reviews [25, 33, 35] explained the methods used to combine findings (Q9) and only one review seemed to have assessed the likelihood of publication bias [35]. This lack of detail in reporting may be due to restrictions on word limits in publications; we did not contact the authors to obtain data when missing.

### Reviews’ findings – the pain assessment tools

In total, 28 pain assessment tools were assessed in the eight reviews; nine tools (Abbey Pain Scale, ADD Protocol, CNPI, DS-DAT, Doloplus-2, NOPPAIN, PACSLAC, PADE, PAINAD) were assessed in five or more reviews; one tool (MOBID) was assessed in three reviews; three tools (Behavior checklist, Observational Pain Behaviour Tool and PATCOA) were assessed in two reviews and the remaining 15 tools were assessed in one review each (see Table 7; a summary of each provided in Additional file 7).

It should be noted that there seem to be different versions of PACSLAC: a preliminary 60 items one, then modified to 36 items. There seems to be ambiguity about which version of the tool the data are reported about (a Dutch version – PACSLAC-D was also mentioned [25]). Similar ambiguity was found in relation to PADE, being unclear which version – or which of its subscales - was studied for psychometric properties. Similarly, the MOBID tool has been studied in two different versions. It is also unclear how much the Abbey Pain Scale had been refined across the studies carried out to evaluate it (including a Japanese version).

### Description of the tools

The reporting of the tools’ content and intended use was done differently by different reviews, making it difficult to provide a comprehensive comparative descriptive summary of all the 28 tools. Six of the eight reviews [21, 24, 25, 30, 34, 36] provided summary tables giving an overview description of tools’ design but these summaries focus on a varied range of aspects including: target population [24], number of items [21, 24, 25], type of behaviours identified [30, 33, 34], number of dimensions/behaviours [21], presence of the American Geriatric Society (AGS) categories [24, 33, 36] and scoring range [21, 24].

From the eight reviews, it appears that most tools (24 out of 28) are observational (Additional file 7), requiring observations by healthcare professionals. However, reviewers’ classification of these observational tools varied: FLACC was described as a ‘behavioural scale’ rather than observational [33]; Abbey Pain Scale, PADE and PAINE were classified by one review [25] as ‘caregiver or informant rating scales’; the same review [25] classified the ADD tool as ‘interactive’– i.e. an “interactive method” including “a physical and affective needs assessment, a review of the patient’s history, and the administration of analgesic medication” [25]. In a number of reviews no specific classification is made. Among the remaining four tools of the 28, one (PPI) relied on patient self–reporting and one (PPQ) was described as relying on caregivers reporting and compared pain experienced currently with pain experienced the previous week.

Twenty-five of the 28 tools appear to include an assessment of pain intensity. Three tools (ADD Protocol, Behavior checklist, Observational Pain Behaviour Tool) aimed at determining presence or absence of pain, with no scoring or rating of pain intensity. In the case of two tools (Doloplus-2 and REPOS) binary scores are summed up and the total score interpreted as presence/absence of pain.

Methods of scoring and rating of pain varied, from scores made of counting checkmarks – i.e. yes/no binary responses (item present or absent), to a variety of rating systems; total scores ranges varied from 0–6 to 0–60 – i.e. 0–6 (CNPI), 0–9 (PATCOA), 0–10 (FLACC, MOBID, PAINAD), 0–14 (ECS), 0–25 (Observational Pain Behaviour Tool), 0–27 (DS-DAT), 0–30 (Doloplus), 0–44 (ECPA), 0–54 (RAPID), and 0–60 (PACSLAC). Likert scales, binary scores, multiple choice and Visual Analogue Scale (VAS) systems were mentioned in the reviews; in three cases (PADE, Pain assessment scale for use with cognitively impaired adults, PPQ) different rating systems are used in the same tool (Likert scales/VAS; Likert scales/binary scores).

Only two out of 28 tools (CNPI, NOPPAIN) appear to be designed explicitly for pain assessment at both rest and during movement – though data about this aspect of the tool’s design may be missing for the other tools. For one tool (Doloplus-2) the score was reported to reflect the progression of the pain experienced, rather than the patient’s pain experienced at a specific moment in time.

Two tools (REPOS and ADD Protocol) combine assessment with guideline for intervention (this is discussed further in the next section).

Three reviews [24, 33, 36] explicitly analyse the tools in terms of whether their design apply the AGS [13] guidelines and categories of potential pain indicators in older persons, namely facial expressions, verbalizations/vocalizations, body movements, changes in interpersonal interactions, changes in activity patterns or routines, and mental status. These reviews cover 15 tools (Abbey Pain Scale, ADD, Behavior checklist, CNPI, DS-DAT, Doloplus-2, FACS, FLACC, MOBID, NOPPAIN, PACSLAC, PADE, PAINAD, PATCOA, and PBM).

### Settings where the tools were studied

The tools were studied in a variety of settings and with varied patient populations. The terminology used to describe settings varied, and those which appeared to be in non-acute settings included: long-term-care, nursing homes, dementia care units, psychogeriatric units, rehabilitation facilities, aged care facilities, residential care facilities, long-term care facilities, palliative care but also, geriatric clinics, care homes, residential and skilled care facilities, long-term-care dementia special care units, and a residential dementia care ward (Additional file 8).

The terminology to refer to hospital settings also varied, with reference either to patients and/or type of services: e.g. hospital patients in a long-term stay department, psychiatric hospital setting, hospital medical care unit, dementia special care units in hospital, hospital patients and older hospital patients.

### Tools psychometric data

#### Reliability

The reliability of pain assessment scores was measured using inter-rater reliability (agreement between raters), test-retest (extent to which a tool achieves the same result on two or more occasions when the condition is stable) or intra-rater reliability (agreement of the same rater at different time points) and internal consistency (Additional file 9). There were no reliability data available for four of the tools (ECS, Pain assessment scale for use with cognitively impaired adults, Observational Pain Behaviour Tool and Behavior Checklist). Overall, reliability measures were carried out on small samples of patients and raters, so data for all of the tools are limited.

##### Inter-rater reliability

This was calculated in different ways for each of the tools. Methods included percentage agreement, kappa coefficients, correlation coefficients, and intra-class correlation coefficients. The variation in calculation of reliability of the different tools makes direct comparisons difficult. Percentage agreement is the least robust measurement of reliability, and was used to calculate agreement for the FACS (43-93%), CNPI (93%), DS-DAT (84-94%), PACSLAC (94%), PATCOA (56.5-100%), NOPAIN (82-100%), and ADD protocol (86-100%). The kappa coefficient measures agreement between two observers and takes into account the agreement expected by chance. It is therefore a more robust measure than percentage agreement. With Kappa coefficients a value of 0.6 or above indicates moderate agreement. Kappa coefficients were provided for the FLACC (0.404), Mahoney Pain Scale (0.55-0.77), CNPI (0.625-0.819), MOBID (0.05-0.90), MOBID-2 (0.44-0.90), NOPAIN (0.70-0.87). Correlation coefficients were used to assess agreement for the following tools; FACS (0.82-0.92), PAINE (0.711-0.999), RaPID (0.97), DS-DAT (0.61-0.98), PAINAD (0.72-0.97). Measures of inter-rater reliability using intra-class correlations were as follows: CPAT (0.71), PBM (0.10-0.87), DS-DAT (0.74), Doloplus-2 (0.77-0.90 total scale, 0.60-0.96 subscales), PACSLAC (0.77-0.96), PADE (range from 0.54-0.96), ECPA (0.80), EPCA-2 (0.852-0.897), MOBID (0.70-0.96), and Abbey pain scale (0.44-0.845). There were no inter-rater reliability data provided for the PPQ.

Overall, the majority of the tools assessed had moderate to good inter-rater reliability. However, there were limitations in terms of the sample sizes used to evaluate their reliability.

##### Test-retest and intra-rater reliability

Intra-rater reliability was not assessed for the FLACC, Mahoney Pain Scale, PBM, PPI, PAINAD, PATCOA, ECPA, EPCA-2 and the ADD protocol. Evaluations of intra-rater reliability included percentage agreement, correlation, kappa, Nygard test-retest and intra-class correlations. In terms of intra-rater reliability, the variation in calculations makes direct comparison across the tools difficult and the use of small sample sizes indicates that all of the results should be treated with caution. Percentage agreement for intra-rater reliability was provided for the FACS (79-93%), correlations for the FACS (0.88-0.97), PAINE (0.711-0.999) and RaPID (>0.75), DS-DAT (0.6), kappa coefficients for MOBID-2 (0.41-0.83 (pain behaviour), 0.48-0.93 (visual pain recordings)), Nygard test-retest for the CNPI (0.23-0.66) and intra-class correlations for the CPAT (0.67), REPOS (0.90-0.96), PACSLAC (0.72-0.96), PADE (0.70-0.98), MOBID (0.60-0.94), and Abbey Pain Scale (0.657). As with inter-rater reliability, the values indicate moderate to good temporal stability.

##### Internal consistency

Internal consistency data were available for the Mahoney Pain Scale (total scale a = 0.76, subscales range 0.68-0.75), PAINE (0.75-0.78), RaPID (0.79), REPOS (0.49), CNPI (0.54-0.64), Doloplus-2 (0.668-0.82), PACSLAC (0.74-0.92), PADE (0.24-0.88), PAINAD (0.5-0.74, PATCOA (0.44), ECPA (0.70), EPCA-2 (0.73-0.79), MOBID (0.82-0.91), MOBID-2 (0.82-0.84), and Abbey Pain Scale (0.645-0.81). There was considerable variation in the internal consistency of scales, with the MOBID and MOBID-2 indicating the highest internal consistency and the PADE, PATCOA and PAINAD having some of the lowest ratings.

### Validity

The validity of the pain tools was primarily explored using concurrent and/or criterion validity (correlation of the pain scale with other pain scores or a benchmark criterion) and/or discriminant and/or predictive validity (e.g. ability to discriminate, or predict between pain on movement and at rest) (Additional file 9). Some reviews (e.g. [21, 33]) also provided brief insight into the conceptual foundation of the measures and ways content validity was explored. As with measures of reliability, there was considerable variation in how the validity of tools was assessed. Three tools had no validity assessment (the Comfort checklist, the Pain assessment scale for use with cognitively impaired adults and the Observational Pain Behaviour Tool). The Non-Communicative Patient’s Pain Assessment Instrument (NOPAIN) tool also had little overall formal validity assessment.

#### Content validity

In general, only limited insight was provided into the conceptual foundation of the tools (as opposed to the tool’s purpose) (Additional file 10). For the vast majority of tools, their derivation, and thus the implied conceptual basis, lay in literature reviews and/or clinical and/or research experts in pain and older patients with dementia. For other tools, for example, the Abbey Pain Scale, its basis was unclear or, as with the Behaviour Checklist, no information was provided. Two of the measures were adapted from measures originally developed for a different patient group, in particular, young children (Doloplus-2; ECPA). In contrast, the purpose of all the measures was commonly outlined. It is notable that some were developed for particular users (CPAT, for certified nursing assistant care providers; NOPPAIN, for nursing assistants), another for research purposes (DS-DAT) and two as decision support tools (the ADD Protocol and the REPOS).

#### Concurrent and criterion validity

Concurrent and criterion validity were measured by either comparing the scores of one tool to another, or comparing one tool’s scores with nurse/doctor ratings of pain, or through comparison with self-report (using VAS scales) (Additional file 11). The following is a summary of the comparisons:

- CPAT was compared to DS-DAT (rs = 22, p = 0.076, rs = 0.25, p = 0.048)
- PAINAD compared to the DS-DAT (0.56-0.76)
- DS-DAT compared to the Pittsburgh Agitation Scale (0.51) and the Cohen-Mansfield Assessment Inventory (0.25)
- Doloplus 2 compared with the PAINAD (0.34) and PACSLAC (0.29-0.38)
- REPOS compared to PAINAD (0.61-0.75)
- FACS was compared to PBM (0.02-0.41)
- PAINE compared with PADE (r = 0.65)
- PADE compared to CMAI (0.30 – 0.42)
- PPI compared with the Memorial pain Subscale (0.67), Verbal scale (0.54), RAND Health Survey and Dartmouth COOP chart (0.72)
- RaPID compared to McGill pain scale (0.8-0.86).

Comparisons to proxy pain reports (doctor or nurse) was as follows; Mahoney pain scale (k = 0.86), PAINAD (0.84), the PBM (0.62-0.73), MOBID (0.41-0.64), Abbey Pain Scale (0.586), PACSLAC (0.35-0.54), and REPOS (−0.12-0.39).

Comparison to self-report (using a VAS) included RaPID (0.8-0.86), EPCA-2 (0.846), DS-DAT (0.56-0.81), PAINAD (0.75 pain VAS and 0.76 discomfort VAS), ECPA (0.67), Doloplus 2 (0.31-0.65), PPI (0.55), CNPI (0.30-0.50), PATCOA (0.41), and PBM (r = 0.11-0.30).

Overall, the tools which had the highest correlations with each other were the RaPID when compared to the McGill pain scale, the REPOS compared to PAINAD and the PPI compared to the Memorial pain Subscale. The Mahoney pain scale and the PAINAD had the highest correlation with nurse/doctor ratings of pain, and the RaPID with self-reports of pain/discomfort. There was no one scale that appeared to be superior to the others (nor applicable as a gold standard), and no consistency in comparisons across the scales.

#### Discriminant validity

Discriminant validity was measured by comparing scores before or after a painful event. Several of the reviews reported that tools had discriminant or predictive validity without providing data – this included the reviews of the FACS and PBM. Other scales with a significant difference in scores pre and post interventions/events included the CPAT, CNPI, DS-DAT, PACSLAC, MOBID, Abbey Pain Scale, ADD protocol, and the Behaviour checklist.

#### Construct validity

Construct validity was measured by comparing scores to medication use or prescription of medications. The PPQ scores were correlated to pain medication use (0.37-0.55), and patients assessed with the PADE on psychoactive medications had significantly higher scores on the physical and verbal agitation subscales. With the PAINAD there was a significant fall in score after the administration of pain medication, and the EPCA-2 was correlated with the prescription of opioids (0.782) and non-opioids (0.730).

### Feasibility and clinical utility

The feasibility of a tool is “its applicability in daily practice”, including aspects such as ease of use and time to administer it, while clinical utility is “the usefulness of the measure for decision making”, i.e. to inform further action, such as the administration of analgesics [24]. Data on feasibility and clinical utility of tools were very limited (Additional files 12 and 13). Often data were not available in the reviews, or when data were available, it often pertained to a lack of data in the original studies (e.g. reviewers stating the item was not reported and could therefore not be assessed). More specifically: feasibility data were completely absent for six tools (Comfort Checklist, FLACC, PAINE, PATCOA, PPI, and PPQ); clinical utility data were completely or substantially absent for seven tools (ECS, FACS, Mahoney Pain Scale, PAINE, PBM, PPQ, and RaPID). For four tools reviewers explicitly noted that claims of feasibility (e.g. time required to administer the tool) were made from the authors of the study without supporting evidence (Abbey Pain Scale, Doloplus-2, PACSLAC, and PADE). There were also two instances (PACSLAC and MOBID) of conflicting data on clinical utility and feasibility from the different reviews, possibly due to an ambiguous reference to different versions of the same tool.

Specific evaluation for feasibility appears to have been carried out only for three tools - CPAT, Mahoney Pain Scale, and Pain Assessment Scale for Use with Cognitively Impaired Adults; in the first two of these cases the evaluation was done by use of questionnaires. It also appeared that users of the Abbey Pain Scale were asked for feedback in the context of the psychometric testing of the tool. In was unclear whether the ADD Protocol was also assessed for feasibility.

Specific evaluation of clinical utility appeared to have been undertaken for the Pain Assessment Scale for Use with Cognitively Impaired Adults, and possibly for the ADD Protocol and PAINAD.

It must be stressed that when reviews assessed or mentioned the feasibility and/or clinical utility of the tools, the two aspects were often confounded (reviewers, authors or users typically drawing conclusions from ease of use or brevity of a scale to its *usefulness*).

Specific dimensions of feasibility assessed were: *time to complete the assessment* (e.g. to complete a checklist), *availability of instructions* on how to use the tool and/or *availability of guidelines on how to score* pain, and *training needs* (Additional file 12).

Six tools were reported to be overall ‘easy to use’ (Abbey Pain Scale, Behavior checklist, CNPI, CPAT, Mahoney Pain Scale, and NOPPAIN), two were considered manageable/acceptable (ECPA, and RaPID), four were judged to be complex (ADD Protocol, DS-DAT, Pain assessment scale for use with cognitively impaired adults, and PADE). Conflicting views on the ease of use or complexity of the tools were apparent for five tools (Doloplus-2, MOBID, PACSLAC, PAINAD, and PBM).

Instructions for use and/or guidelines on scoring were reported to be available for 13 tools (Abbey Pain Scale, CNPI, CPAT, Doloplus-2, DS-DAT, ECS, FACS, Mahoney Pain Scale, MOBID, NOPPAIN, PACSLAC, PAINAD, REPOS) with varied assessments in terms of clarity or complexity of the instructions.

Training in the use of the tool was judged as necessary for 10 tools (EPCA-2, Mahoney Pain Scale, NOPPAIN, PADE, PACSLAC, PAINAD, ADD Protocol, CPAT, DS-DAT, and MOBID), four of which seemed to require significant training (ADD Protocol, CPAT, DS-DAT, and MOBID). For six tools it was stated that authors of studies/tools did not report on the level and length of training required (Abbey Pain Scale, CNPI, Doloplus-2, PAINAD, PACSLAC, REPOS). For the majority of the tools however, data about training were not available (Behavior checklist, Comfort Checklist , ECPA, ECS, FACS, FLACC, Observational Pain Behaviour Tool, Pain assessment scale for use with cognitively impaired adults, PAINE, PATCOA, PBM, PPI, PPQ, and RaPID).

Specific dimensions of clinical utility were less straightforward. The *availability of cut-off scores* and *of interpretation of scores for decision making* appeared to be the two dimensions supporting evidence of clinical utility. The presence of cut-off scores contributes to achieving clinical utility, for example to help discriminate between presence and absence of pain, or to couple the scale with a treatment algorithm. Otherwise, general statements were available related to e*vidence of use in clinical settings* and *evidence of clinical utility,* the latter being dependent on the first (Additional file 13).

Cut-off scores appeared to be available only for REPOS and Doloplus-2, though in the latter case they still need to be validated. The availability of guidance on how to interpret the scores and for further action following assessment was variedly reported. Data on this were missing for 18 tools (ADD Protocol, Behavior checklist, Comfort Checklist, DS-DAT, ECS, EPCA-2, FACS, FLACC, Mahoney Pain Scale, MOBID, Pain Assessment Scale for Use with Cognitively Impaired Adults, PAINE, PATCOA, PBM, PPI, PPQ, RaPID, and REPOS). For four tools it appeared that interpretations of the scores were available (Abbey Pain Scale, Doloplus-2, CNPI, and CPAT) though in two of these they were deemed unclear (CNPI, and CPAT). For five tools, it appeared interpretations of the scores were not available (ECPA, NOPPAIN, Observational Pain Behaviour Tool, PACSLAC, and PADE) and further four were reported as lacking guidance for further action following assessment (CNPI, CPAT, Doloplus-2, and NOPPAIN). It was unclear whether PAINAD does provide interpretation of scores.

Suggestive *evidence of use* in clinical settings was reported for two tools: Abbey Pain has been incorporated into the Australian pain guidelines; and the ADD Protocol was introduced in 57 long term care facilities together with an education strategy for 12 months and the evaluation was done in a study with 32 nurses in 25 facilities. It is otherwise unclear whether any of the other tools were actually used in practice beyond a period of research or testing of the tool. In one example (NOPPAIN), the testing of the tool was done by use of video recording of an actress portraying a bed-bound patient, and it remains unclear whether further testing was done in clinical setting or with patients at bedside.

With the exception of the ADD Protocol and REPOS, there was no mention of how the tools would inform intervention (e.g. choice of treatment). We found an overall lack of clarity in the descriptions of the ADD Protocol, but it seems that one of its strengths is that it links observation of behaviour with interventions. Similarly, one review suggested that the clinical utility of REPOS potentially resides in its combination with a decision tree to assist in determining interventions after pain assessment [35].

### Overall assessment of the tools

There seemed to be a general consensus among the reviewers that the current evidence on validation and clinical utility of the tools is insufficient (Additional file 14). The overall conclusion was that there is a need for further psychometric testing of each tool. Two reviews recommended that the focus should be on studying existing scales rather than creating new ones [21, 30], although one review also suggested that there may be a need to revisit the tools’ conceptual foundations [33].

Recommendations for further research and testing of the tools included the involvement of culturally diverse populations [21, 33, 36] and the provision of scoring methods and guidelines for interpretation in the evaluation of the scale [36]. Finally, a need for research emerged to link assessment with treatment algorithms [24].

Some of the reviews also concluded with recommendations for practice, for example: the use of at least two different pain assessment approaches at the same time in clinical practice and two different tools in research [25]; the importance of a comprehensive approach to pain assessment beyond the use of tools [33]; the need to involve social workers in regular holistic multidisciplinary pain assessment (in nursing homes), with training in the use of the scales [36].

Among the tools selected by the reviews as possible best candidates, albeit on limited evidence, were the DS-DAT, Doloplus 2, Mahoney Pain Scale, PACSLAC, PAINAD, Abbey Pain Scale, and ECPA. The ADD protocol was mentioned as an example of a more comprehensive approach for the identification of pain, beyond the use of an assessment tool as “a standardized tool is only one step in a complex diagnostic process” [33]. There was also agreement on recommending that patients with dementia can often reliably verbalise their pain, suggesting therefore that the use of observational scales should be limited to patients who demonstrably cannot reliably verbalise their pain.

## Discussion

The objective of our meta-review was to identify which tools are available to assess pain in adults with dementia, in which settings and patient populations they have been used, and evaluate their reliability, validity, feasibility and clinical utility. We found a relatively large number of reviews on this topic and a considerable number of pain assessment tools available for the cognitively impaired population. Of the reviews we evaluated, only one [35] met all of the quality criteria outlined in the AMSTAR checklist, and would be considered a high quality review. The review by Herr et al. [33] has an associated website where 17 tools (specifically for use in nursing homes) are listed and commented on in detail [50].

The systematic reviews commonly situated their choice of interventions to review in the context of the challenges associated with assessing pain in patients who were cognitively impaired. Whilst there was recognition that the gold standard for pain assessment was the patient’s own assessment, this was unlikely to be possible with this patient population if the person was severely cognitively impaired, thus leading to the use of observational scales. The tools included in these reviews were therefore for the greater part observational, but showed a broad variation of measures and methods of assessing pain in adults with dementia. The foundation and focus of an observational scale is *how the pain is manifested or made known* such as on the basis on the American Geriatric Society (AGS) Pain indicators [13]. An additional conceptual foundation resided on a differentiation of aspects of pain – such as “the sensory-discriminative and motivational-affective aspects” [43]. Very few tools appeared to have any strong theoretical underpinning to the development.

Overall there was *no* one tool that appeared to be more reliable and valid than the others. There was considerable variation in how reliability and validity of the tools were assessed. The majority of reliability and validity assessments were carried out on small samples in one or two different studies – so the applicability of tools across settings is yet to be evaluated. Similar conclusions can be drawn in relation to the feasibility and clinical utility of the tools. These findings have implications for research, which we briefly discuss over four points.

First, given the large number of existing tools identified within our meta-review, it seems inappropriate to develop further tools if on the basis of the same conceptual foundations. Instead, researchers need to either envision new assessment instruments on the basis of different conceptual foundations, or concentrate on extending the psychometric evidence base for the existing tools. There is a need to evaluate the tools on a wider scale, across a variety of patients and clinical settings, using rigorous methods and larger sample sizes. The studies need to ensure that the definitions of cognitive impairment and type of pain that is being assessed are clearly defined, to enable comparisons across populations. While rigorous research is needed, it must be noted that the accuracy of tools is difficult to assess when an objective biological marker or other gold standard criterion is lacking - such as is the case for pain intensity [35, 48, 49].

Second, research should be conducted in clinical practice to assess the feasibility and clinical utility of the tools, and thus their potential for use in everyday clinical practice. Tools may be more useful in detecting relative changes in individual patients than differences between patients [41]. Tools that showed high reliability in research may not display to be highly reliable in routine clinical practice if not administered as intended [51]. Pain assessment tools designed for research purposes, in order to aggregate and compare data across patients, do not necessarily transfer easily and effectively to clinical settings for everyday use. In general it has been suggested that measurement tools developed for the purpose of evaluation of policy making [52, 53] or routine general screening [51] might have ‘no meaning’ at the frontline.

Third, research on the clinical utility of the tools should include evaluation of their impact in terms of choice of treatment and patient outcomes. This question was considered in one of the excluded reviews [48]: the reviewers found no evaluations of the effect of protocols such as algorithms or pathways for assessing pain in inpatients (including those with dementia) and they reported “a study in cancer patients [that] found opioid-related over sedation and other adverse effects increased substantially after implementation of pain assessment on a numerical scale routinely with other vital signs” ([48], p11, citing [54]). Herr et al. [33] reported that “the use of the ADD Protocol was associated with a significant increase in the use of pharmacologic […] and non-pharmacologic comfort interventions” (p179). Alternatively, the use of the wrong pain assessment tool might reduce the likelihood that pain treatment will be initiated or contribute to acute exacerbations of pain (as could be inferred by data from [34]), or may be found to have no effect on the quality of pain management [55].

Finally, an instrument must be relevant to “the condition, setting and participants in the health interaction, in particular the patient and clinical users” [56]. This includes the notion of *user-centredness*, defined as “the extent to which an instrument faithfully captures both the content of the health care user’s views and the form or ways in which their views are expressed” [57]. As a number of the systematic reviews and other literature makes clear, pain is a subjective experience; thus the associated measurement gold standard is the patient’s own assessment [24]. However, where the patient is severely cognitively impaired, alternative ways to assess the patient’s level of pain must be found. Resort is made to behavioural indicators and groups of cues [58]. Ideally, these aspects would be used to assess expressed pain when the patient is at rest and when moving. Against this context, user-centredness, in terms of the actual patient’s self-assessment of pain, is unachievable. The next best option, from a user-centred perspective, becomes the assessment of a person who is *most familiar* with the patient in their everyday life in a hospital or other care setting – what Herk et al. [24] call a “silver standard”. It is critical, however, that the assessor has a high familiarity with the patient. It can plausibly be argued that the more this is achieved, the greater the likelihood of a closer fit of the proxy’s view with the patient’s own experience. At the same time, over-familiarity may lead to an attenuation of the observer’s focus on potential pain clues. However, as Smith [34] commented, one is rarely in ‘an ideal situation’ where the direct care providers know, and are thus highly familiar, with the care recipient’s personal habits and history. Thus, rather than seeking to review the degree of the user-centredness of tools in this area, it is important to evaluate the *guidance on a tool’s us*e (that is, who should administer the intervention and in what range of situations) and then explore whether or not it is *actually used* in the indicated way in everyday clinical practice. Exploration and findings on clinical utility and feasibility of use provide suggestive evidence of potential for use in everyday practice. Such evidence is a necessary but not sufficient condition for a tool’s actual use. It is notable that little or no insight is provided into the guidance over who should administer the tools in any of the systematic reviews. To gain insight into this aspect of use and possible additional evidence on a tool’s actual use in practice, one would need to examine the original instrument designers’ papers and validation studies, rather than rely on a systematic reviewer’s observations or summaries. Notwithstanding, it can be recommended that these two aspects of tool use (guidance and actual use) are incorporated into tools reviews and, given this patient group, development of evidence on the actual use of the tools in clinical practice.

### Overall completeness and applicability of evidence

We analysed 23 reviews for data extraction and included data from eight of these. Whilst we could have been more strict in our interpretation of the criteria for a systematic review and exclude a greater number of potentially eligible records before reaching the stage of data extraction, this would not have necessarily restricted the analysis to reviews with higher AMSTAR scores. It would have given us a smaller number of records for data extraction, and while this would have saved us time in the data extraction process, we do not believe that it would have changed the final outcome and the findings. It would also have reduced the number and range of tools assessed.

We analysed 28 tools included in eight reviews. However other tools are also available and were included in some of the reviews we excluded because they did not provide psychometric data for extraction. The review by Stolee et al. [49], for example, covers 30 tools, including The Proxy Pain Questionnaire, The Pain Behavior Measure, the 21-Box Scale, and the Pain Thermometer, among others.

Furthermore, this meta-review does not cover a new tool recently developed in France by the Doloplus Collective team – ALGO Plus [59]. To our knowledge, the tool has not been included yet (at the time of writing) into a systematic review.

### Potential biases in the overview process

We checked further available data when readily available, such as in supplementary tables online [25] but we did not communicate with the authors to obtain missing data. This may have introduced publication bias.

In our assessment of the reliability and validity of the tools, we did not attempt a meta-analysis; we are aware that studies had been counted in various reviews and their results could have been counted more than once. However, we do not believe this to be an issue for our narrative synthesis.

Our search did not specifically target reviews reporting on the feasibility or clinical utility of the tools – thus our evidence may be more limited on these aspects than it might have been otherwise.

We attempted to minimise bias in the review process by involving a review team with diverse expertise; by having each review assessed independently by at least two members of the team, and the whole team of reviewers involved at every stage of the process; by not excluding reviews on the basis of our assessment of their quality.

## Conclusions

The assessment of pain in patients with dementia is challenging for clinicians, due to some patients’ inability to verbalise the nature of their pain. This review highlights the current state of the evidence base in relation to pain assessment tools, and provides insights into current gaps in our understanding. We identified a total of 28 tools that could possibly be used in clinical practice to help with this process; however we cannot at present recommend any particular tool for use in any clinical setting, due to the lack of comprehensive evidence on the reliability, validity, feasibility or clinical utility of any one particular tool. Further research should be conducted on the psychometric properties of tools and in clinical practice to assess feasibility, clinical utility and guidance on use of the tools. These are necessary albeit not sufficient pre-requisites for actual use in routine clinical practice. Throughout, the aim remains the same: to gain as good an assessment as possible of the patient’s pain with a view to effective pain management.

## Electronic supplementary material

Additional file 1: **Literature Search.** Details of the retrieval process, including databases searched, adjustments to the search strategy first optimised for the OVID MEDLINE database, for use in other databases, and detailed search strategies for each database. (PDF 273 KB)

Additional file 2: **Notes on screening titles abstracts.** Details of the screening process on the basis of title and abstract of the records retrieved, how it was shared among the reviewers so that each record was screened by two independent reviewers, and the number of records referred for a third reviewer. (PDF 143 KB)

Additional file 3: **Notes on screening fulltext.** Details of the screening process on the basis of the full text of the records retrieved, how it was shared among the reviewers so that each record was screened by two independent reviewers and the number of records referred for a third reviewer. (PDF 73 KB)

Additional file 4: **Data Extraction forms.** The data extraction forms used to build a data extraction database and extract data for the meta-review. (PDF 169 KB)

Additional file 5: **Summary of reviews methods.** Overview of the included reviews, with a description of the aims and the methods used for including and analysing the studies, their quality assessment of the studies, and the tools examined. (DOCX 31 KB)

Additional file 6: **Sample of studies methodological weaknesses identified by the reviews.** A sample of studies’ methodological challenges and weaknesses identified in/by the reviews, organised by type of issue (e.g. sampling, tool design). (DOCX 24 KB)

Additional file 7: **Summary of the tools - characteristics.** Summary of the data extracted from the reviews providing a classification and description of the tools. (DOCX 74 KB)

Additional file 8: **Summary of the tools - settings of use.** Summary of the data extracted from the reviews pertaining to the settings were the tools had been studied, tested or used for clinical practice. (DOCX 21 KB)

Additional file 9: **Summary of the tools - reliability and validity.** Summary of the data extracted from the reviews regarding the reliability and validity of each tool. (DOCX 48 KB)

Additional file 10: **Summary of tools - Content-validity-User centredness.** Analysis of the data extracted from the reviews regarding conceptual foundation of each tool and ways content validity was explored. These aspects also pertain to the user-centredness of the tools. (DOCX 26 KB)

Additional file 11: **Tools concurrent and criterion validity comparison table.** Summary of the data on the concurrent and criterion validity of the tools, extracted from the reviews. (DOCX 27 KB)

Additional file 12: **Summary of tools – feasibility.** Summary of the data on the feasibility of each tool, extracted from the reviews, analysed in terms of ‘dimensions’ of feasibility – ease of use, time to complete, availability of instruction and guidelines, training required. (DOCX 35 KB)

Additional file 13: **Summary of tools - clinical utility.** A summary of the data on the clinical utility of each tool, extracted from the reviews, analysed in terms of ‘dimensions’ of clinical utility (availability of cut-off scores and interpretation of scores for decision making) and overall evidence. (DOCX 31 KB)

Additional file 14: **Summary of reviews conclusions and recommendations.** Summary of the overall conclusions and recommendations of each review and the tools considered in each of them. (DOCX 27 KB)

## Abbreviations

ADD Protocol: Assessment Discomfort in Dementia Protocol
CNPI: Checklist of Nonverbal Pain Indicators
CPAT: Certified Nurse Assistant Pain Tool
DS-DAT: Discomfort Scale - Dementia of the Alzheimer’s Type
ECPA: L’Echelle Comportementale pour Personne Agées
ECS: Edmonton Classification System
EPCA-2: Elderly Caring Assessment 2
FACS: Facial Action Coding System
FLACC: Face, Legs, Activity, Cry, Consolability Scale
MPS: Mahoney Pain Scale
MOBID: Mobilization, Observation, Behavious, Intensity, Dementia Pain Scale
NOPPAIN: Non-Communicative Patient’s Pain Assessment Instrument
PACSLAC: Pain Assessment Checklist for Seniors with Limited Ability to Communicate
PADE: Pain Assessment in Dementing Elderly
PAINAD: Pain Assessment in Advanced Dementia
PAINE: Pain Assessment in Noncommunicative Elderly persons
PATCOA: Pain Assessment Tool in Confused Older Adults
PBM: Pain Behaviour Measurement approach, also known as Pain Behavior Method [24]
PPI: Present Pain Intensity
PPQ: Proxy Pain Questionnaire
RaPID: Rating Pain in Dementia
REPOS: Rotterdam Elderly Pain Observation Scale
VAS: Visual Analogue Scale.
