# Supplementary material for: Pain assessment for people with dementia: a systematic review of systematic reviews of pain assessment tools
Source: BMC Geriatr. 2014 Dec 17;14:138. doi: 10.1186/1471-2318-14-138 (PMC4289543; doi:10.1186/1471-2318-14-138)
Supplement: Supplementary file 1 — Additional file 1: Literature Search. Details of the retrieval process, including databases searched, adjustments to the search strategy first optimised for the OVID MEDLINE database, for use in other databases, and detailed search strategies for each database. (PDF 273 KB) [file 12877_2014_1072_MOESM1_ESM.pdf]

Pain assessment for people with dementia: a systematic review of systematic reviews of pain assessment tools

Literature Search

15 March 2013

This document contains details of the retrieval process for the meta-review "Pain assessment for people with dementia: a systematic review of systematic reviews of pain assessment tools". The search strategy was first optimised for the OVID MEDLINE database and then adapted to be applied to other online databases.

Contents

DB searches 2

Database-specific search strategies – adjustments to Medline baseline search 3

Search strategies: 5

Database searches

Date / time	Database	# records retrieved (including duplicates)	# records retrieved (excluding duplicates)
12 March 2013 11:12	MEDLINE (specifically: Ovid MEDLINE 1946 to February Week 4 2013)	209	208
12 March 2013 11:22	Ovid MEDLINE In-Process & Other Non-Indexed Citations March 11, 2013	0	0
12 March 2013 11:14	All EBM Reviews - Cochrane DSR, ACP Journal Club, DARE, CCTR, CMR, HTA, and NHSEED	68	67
12 March 2013 12:05	Embase (1996 to 2013 Week 10)	74	73
12 March 2013 12:22	PsycINFO (1806 to March Week 1 2013)	68	0
12 March 2013 13:40	CINHAL	78	0
12 March 2013 14:15	The Joanna Briggs Institute (JBI) Library - The JBI Database of Systematic Reviews and Implementation Reports[1]	6	0
12 March 2013 14:49	Centre for Reviews and Dissemination database[2]	5	0
		Tot: 508	441[3]

[1] http://www.joannabriggslibrary.org/index.php/jbisrir/search/results
[2] http://www.crd.york.ac.uk/crdweb/SearchPage.asp
[3] 67 duplicates – i.e. 11 records retrieved in 2 or more databases

Database-specific search strategies – adjustments to Medline baseline search

Database	Strategy
MEDLINE	(terms optimised for this database; baseline for other searches)
Ovid MEDLINE(R) In-Process & Other Non-Indexed Citations	Same as MEDLINE
All EBM Reviews - Cochrane DSR, ACP Journal Club, DARE, CCTR, CMR, HTA, and NHSEED	Modified MEDLINE : removed the following terms:<br>*Pain Measurement/mt<br>exp *Pain Measurement/<br>(symbols not recognised by the database; retrieved 0 items)
Embase	Modified MEDLINE :<br>removed the following terms:<br>[exp Pain/di]*Pain Measurement/mt<br>meta-analysis.pt.<br>AND limited with search to max precision (study design criterion: systematic review)
PsycINFO	Modified MEDLINE :<br>removed the following terms:<br>exp Cognition Disorders/<br>[exp Pain/di]<br>[*Pain Measurement/mt]<br>meta-analysis.pt.<br>review.pt.<br>Added the following term (to compensate for review.pt. now removed from the strategy):<br>review.mp

CINHAL (Cumulative Index to Nursing & Allied Health Literature[4]	Modified MEDLINE, with the following changes:<br><br>exp Dementia/ modified as: MW Dementia [word in subject heading]<br><br>exp Alzheimer Disease/ removed (not recognised as subject heading)<br><br>Cognition Disorders/ modified as: Cognition Disorder* [all text]<br><br>exp mental retardation/ modified as: mental retardation [word in subject heading]<br><br>Assess$ adj5 pain Changed to Assess* N5 pain<br><br>Measur$ adj5 pain Changed to Measur* N5 pain<br><br>Scale$ adj5 pain Changed to Scale* N5 pain<br><br>Rating adj5 pain Changed to Rating N5 pain<br><br>exp Pain Measurement/ modified as: MW Pain Measurement [word in subject heading]<br><br>exp Pain/di modified as: MW Pain Diagnosis [word in subject heading]<br><br>*Pain Measurement/mt removed<br>exp *Pain Measurement/ removed<br><br>Pain adj3 tool$ Changed to Pain N3 tool*
The Joanna Briggs Institute (JBI) Library - The JBI Database of Systematic Reviews and Implementation Reports[5]	Simple search for pain and (dementia or cognitive impairment)
Centre for Reviews and Dissemination database[6]	Simplified search

[4] Available via EBSCO at: http://web.ebscohost.com/ehost/search/advanced?sid=86a5a873-04e4-44fa-9084-2b30aae017c5%40sessionmgr14&vid=2&hid=21
[5] http://www.joannabriggslibrary.org/index.php/jbisrir/search/results
[6] http://www.crd.york.ac.uk/crdweb/SearchPage.asp

Search strategies:

Medline	1. Dementia.mp. [mp=title, abstract, original title, name of substance word, subject heading word, keyword heading word, protocol supplementary concept, rare disease supplementary concept, unique identifier]<br>2. Alzheimer.mp. [mp=title, abstract, original title, name of substance word, subject heading word, keyword heading word, protocol supplementary concept, rare disease supplementary concept, unique identifier]<br>3. exp Dementia/<br>4. exp Alzheimer Disease/<br>5. 1 or 2 or 3 or 4<br>6. exp Cognition Disorders/<br>7. Cognitive impairment.mp. [mp=title, abstract, original title, name of substance word, subject heading word, keyword heading word, protocol supplementary concept, rare disease supplementary concept, unique identifier]<br>8. Cognitive function*.mp. [mp=title, abstract, original title, name of substance word, subject heading word, keyword heading word, protocol supplementary concept, rare disease supplementary concept, unique identifier]<br>9. exp mental retardation/<br>10. 6 or 7 or 8 or 9<br>11. 5 or 10<br>12. (Assess$ adj5 pain).mp. [mp=title, abstract, original title, name of substance word, subject heading word, keyword heading word, protocol supplementary concept, rare disease supplementary concept, unique identifier]<br>13. (Measur$ adj5 pain).mp. [mp=title, abstract, original title, name of substance word, subject heading word, keyword heading word, protocol supplementary concept, rare disease supplementary concept, unique identifier]<br>14. (Scale$ adj5 pain).mp. [mp=title, abstract, original title, name of substance word, subject heading word, keyword heading word, protocol supplementary concept, rare disease supplementary concept, unique identifier]<br>15. (Rating adj5 pain).mp. [mp=title, abstract, original title, name of substance word, subject heading word, keyword heading word, protocol supplementary concept, rare disease supplementary concept, unique identifier]<br>16. exp Pain Measurement/<br>17. exp Pain/di<br>18. *Pain Measurement/mt<br>19. exp *Pain Measurement/

	20. (Pain adj3 tool$).mp. [mp=title, abstract, original title, name of substance word, subject heading word, keyword heading word, protocol supplementary concept, rare disease supplementary concept, unique identifier]<br>21. 12 or 13 or 14 or 15 or 16 or 17 or 18 or 19 or 20<br>22. 11 and 21<br>23. meta-analysis.mp.<br>24. meta-analysis.pt.<br>25. review.pt.<br>26. search:.tw.<br>27. 23 or 24 or 25 or 26<br>28. 22 and 27
All EBM Reviews	1. Dementia.mp. [mp=title, abstract, subject headings, heading word, drug trade name, original title, device manufacturer, drug manufacturer, device trade name, keyword]<br>2. Alzheimer.mp. [mp=title, abstract, subject headings, heading word, drug trade name, original title, device manufacturer, drug manufacturer, device trade name, keyword]<br>3. exp Dementia/<br>4. exp Alzheimer Disease/<br>5. 1 or 2 or 3 or 4<br>6. exp Cognition Disorders/<br>7. Cognitive impairment.mp. [mp=title, abstract, subject headings, heading word, drug trade name, original title, device manufacturer, drug manufacturer, device trade name, keyword]<br>8. Cognitive function*.mp. [mp=title, abstract, subject headings, heading word, drug trade name, original title, device manufacturer, drug manufacturer, device trade name, keyword]<br>9. exp mental retardation/<br>10. 6 or 7 or 8 or 9<br>11. 5 or 10<br>12. (Assess$ adj5 pain).mp. [mp=title, abstract, subject headings, heading word, drug trade name, original title, device manufacturer, drug manufacturer, device trade name, keyword]<br>13. (Measur$ adj5 pain).mp. [mp=title, abstract, subject headings, heading word, drug trade name, original title, device manufacturer, drug manufacturer, device trade name, keyword]<br>14. (Scale$ adj5 pain).mp. [mp=title, abstract, subject headings, heading word, drug trade name, original title, device manufacturer, drug manufacturer, device trade name, keyword]<br>15. (Rating adj5 pain).mp. [mp=title, abstract, subject headings, heading word, drug

	trade name, original title, device manufacturer, drug manufacturer, device trade name, keyword]<br>16. exp Pain Measurement/<br>17. exp Pain/di<br>18. (Pain adj3 tool$).mp. [mp=title, abstract, subject headings, heading word, drug trade name, original title, device manufacturer, drug manufacturer, device trade name, keyword]<br>19. 12 or 13 or 14 or 15 or 16 or 17 or 18<br>20. 11 and 19<br>21. meta-analysis.mp.<br>22. meta-analysis.pt.<br>23. review.pt.<br>24. search:.tw.<br>25. 21 or 22 or 23 or 24<br>26. 20 and 25
EMBASE	1. Dementia.mp. [mp=title, abstract, subject headings, heading word, drug trade name, original title, device manufacturer, drug manufacturer, device trade name, keyword]<br>2. Alzheimer.mp. [mp=title, abstract, subject headings, heading word, drug trade name, original title, device manufacturer, drug manufacturer, device trade name, keyword]<br>3. exp Dementia/<br>4. exp Alzheimer Disease/<br>5. 1 or 2 or 3 or 4<br>6. exp Cognition Disorders/<br>7. Cognitive impairment.mp. [mp=title, abstract, subject headings, heading word, drug trade name, original title, device manufacturer, drug manufacturer, device trade name, keyword]<br>8. Cognitive function*.mp. [mp=title, abstract, subject headings, heading word, drug trade name, original title, device manufacturer, drug manufacturer, device trade name, keyword]<br>9. exp mental retardation/<br>10. 6 or 7 or 8 or 9<br>11. (Assess$ adj5 pain).mp. [mp=title, abstract, subject headings, heading word, drug trade name, original title, device manufacturer, drug manufacturer, device trade name, keyword]<br>12. (Measure$ adj5 pain).mp. [mp=title, abstract, subject headings, heading word, drug trade name, original title, device manufacturer, drug manufacturer, device trade name, keyword]<br>13. (Scale$ adj5 pain).mp. [mp=title, abstract, subject headings, heading word, drug

	trade name, original title, device manufacturer, drug manufacturer, device trade name, keyword]<br>14. (Rating adj5 pain).mp. [mp=title, abstract, subject headings, heading word, drug trade name, original title, device manufacturer, drug manufacturer, device trade name, keyword]<br>15. exp Pain Measurement/<br>16. exp Pain/di<br>17. 11 or 12 or 13 or 14 or 15 or 16<br>18. (Pain adj3 tool).mp. [mp=title, abstract, subject headings, heading word, drug trade name, original title, device manufacturer, drug manufacturer, device trade name, keyword]<br>19. exp *Pain Measurement/<br>20. 18 or 19<br>21. Cochrane database of systematic reviews.jn.<br>22. Search.tw.<br>23. Medline.tw.<br>24. Systematic review.tw.<br>25. 21 or 22 or 23 or 24<br>26. 5 or 10<br>27. 17 or 20<br>28. 25 and 26 and 27
PsycINFO	1. Dementia.mp. [mp=title, abstract, heading word, table of contents, key concepts, original title, tests & measures]<br>2. Alzheimer.mp. [mp=title, abstract, heading word, table of contents, key concepts, original title, tests & measures]<br>3. exp Dementia/<br>4. exp Alzheimer Disease/<br>5. 1 or 2 or 3 or 4<br>6. Cognitive impairment.mp. [mp=title, abstract, heading word, table of contents, key concepts, original title, tests & measures]<br>7. Cognitive function*.mp. [mp=title, abstract, heading word, table of contents, key concepts, original title, tests & measures]<br>8. exp mental retardation/<br>9. 6 or 7 or 8<br>10. 5 or 9<br>11. (Assess$ adj5 pain).mp. [mp=title, abstract, heading word, table of contents, key concepts, original title, tests & measures]<br>12. (Measur$ adj5 pain).mp. [mp=title, abstract, heading word, table of contents, key

	concepts, original title, tests & measures]<br>13. (Scale$ adj5 pain).mp. [mp=title, abstract, heading word, table of contents, key concepts, original title, tests & measures]<br>14. (Rating adj5 pain).mp. [mp=title, abstract, heading word, table of contents, key concepts, original title, tests & measures]<br>15. exp Pain Measurement/<br>16. exp *Pain Measurement/<br>17. (Pain adj3 tool$).mp. [mp=title, abstract, heading word, table of contents, key concepts, original title, tests & measures]<br>18. 11 or 12 or 13 or 14 or 15 or 16 or 17<br>19. 10 and 18<br>20. meta-analysis.mp.<br>21. review.mp. [mp=title, abstract, heading word, table of contents, key concepts, original title, tests & measures]<br>22. search:.tw.<br>23. 20 or 21 or 22<br>24. 19 and 23
CINHAL	1. AB meta-analysis<br>2. PT review<br>3. AB search*<br>4. PT systematic review<br>5. 1 or 2 or 3 or 4<br>6. TX Cognition Disorder*<br>7. TX Cognitive impairment<br>8. TX Cognitive function*<br>9. MW mental retardation<br>10. 6 OR 7 OR 8 OR 9<br>11. Dementia<br>12. Alzheimer<br>13. MW Dementia<br>14. 11 OR 12 OR 13<br>15. Assess* N5 pain<br>16. Measur* N5 pain<br>17. Scale* N5 pain<br>18. Rating N5 pain<br>19. MW Pain Measurement<br>20. MW pain diagnosis

	21. TX Pain N3 tool*<br>22. 15 OR 16 OR 17 OR 18 OR 19 OR 20 OR 21<br>23. 10 OR 14<br>24. 23 AND 22 AND 5
The JBI Database of Systematic Reviews and Implementation Reports	"pain assessment" and (dementia or "cognitive impairment")
Centre for Reviews and Dissemination database[7]	1. dementia<br>2. cognitive impairment<br>3. 1 OR 2<br>4. pain NEAR5 (assess* or measur*)<br>5. pain near5 tool*<br>6. 4 OR 5<br>7. 3 AND 6<br>(limited to record type : systematic review)

[7] http://www.crd.york.ac.uk/crdweb/SearchPage.asp
