# Supplementary material for: Pain assessment for people with dementia: a systematic review of systematic reviews of pain assessment tools
Source: BMC Geriatr. 2014 Dec 17;14:138. doi: 10.1186/1471-2318-14-138 (PMC4289543; doi:10.1186/1471-2318-14-138)
Supplement: Supplementary file 2 — Additional file 2: Notes on screening titles abstracts. Details of the screening process on the basis of title and abstract of the records retrieved, how it was shared among the reviewers so that each record was screened by two independent reviewers, and the number of records referred for a third reviewer. (PDF 143 KB) [file 12877_2014_1072_MOESM2_ESM.pdf]

First screening of identified records, on the basis of title and abstract

19 April 2013

441 records were identified through database searches. Two reviewers screened each record for inclusion in the next stage of the review process (i.e. screening on the basis of full text).

In order to have each record screened by two reviewers, a copy of the database was created and then shared as follows:

Original database:

1. [reviewer 1] to review the whole set (441 records)

Copy of the database:

2. [reviewer 2] to review 200 records (ca. 45.3%)

2. [reviewer 3] to review 200 records (ca. 45.3%)

3. [reviewer 4] to review 41 records (ca. 9.3%)

Results

As detailed in Tables below, these were the results of the screening process:

In 36% of the cases (157 records), the reviewers agreed to accept the paper for inclusion to the next stage of the review process by answering either YES or MAYBE to the question ‘Accept?’ (41 records: agreed YES; 24 records: agreed MAYBE, 79 records: disagreement between YES/MAYBE). (see Table 1).

In 46% of the cases (205 records) there was agreement that the paper should *not* be included to the next stage (reviewers answered NO to the question ‘Accept?’).

In 18% of the cases (79 records), reviewers disagreed on whether the paper should be included or not to the next stage. Their answers to the question ‘Accept?’ were a combination of either YES/NO or MAYBE/NO. These records were subject to further review to establish whether to reject them or not.

It was agreed that a reviewer who had not screened the record before would carry out a third review and have the final say. Thus, the subset of 79 records was redistributed to two of the reviewers as follows:

- 18 originally reviewed by [reviewer 1] and [reviewer 2] were sent to [reviewer 3] for a third opinion

- 61 originally reviewed by [reviewer 1] and [reviewer 4] or [reviewer 3], were sent to [reviewer 2] for a third opinion.

For these 79 were the initial two reviewers had not reached consensus, the final say of the third reviewer was (Table 2) that 26 records (33%) were to be accepted to the next stage (screen on the basis of full text) and 53 (67%) were to be excluded.

In summary, at the end of this process, 183 (41%) records were accepted for inclusion to the next stage of the review process; 258 (59%) were rejected.

Table 1. Results of the screening on the basis of title & abstract – 2 reviewers

Screening outcome	Unique records (number)	(%)
Agreement - YES	46	10%
Agreement - NO	205	46%
Agreement - MAYBE	24	5%
Disagreement – outcome: Inclusion (equivalent to Maybe)	87	20%
Disagreement - outcome: potential Exclusion	79	18%
Total	441	100%

Table 2. Results screening on the basis of title & abstract – 3rd reviewer

Screening outcome	Unique records (number)	(%)
[reviewer 2]- YES	23	29%
[reviewer 2]- NO	38	48%
[reviewer 2]- MAYBE	0	
[reviewer 3]- YES	1	1%
[reviewer 3]- NO	15	19%
[reviewer 3]- MAYBE	2	3%
Total	79	100%

Table 3. Overall results of the screening on the basis of title & abstract

Screening outcome	Unique records (number)	(%)
2R: Agreement – YES	46	10%
2R: Agreement – MAYBE	24	5%
2R: Disagreement (YES/MAYBE)	87	20%
3R: Decision – YES/MAYBE	26	6%
Subtotal	[183]	[41%]
2R: Agreement – NO	205	46%
3R: Decision – NO	53	12%
Subtotal	[258]	[59%]
Total	441	100%
