# Supplementary material for: Pain assessment for people with dementia: a systematic review of systematic reviews of pain assessment tools
Source: BMC Geriatr. 2014 Dec 17;14:138. doi: 10.1186/1471-2318-14-138 (PMC4289543; doi:10.1186/1471-2318-14-138)
Supplement: Supplementary file 3 — Additional file 3: Notes on screening fulltext. Details of the screening process on the basis of the full text of the records retrieved, how it was shared among the reviewers so that each record was screened by two independent reviewers and the number of records referred for a third reviewer. (PDF 73 KB) [file 12877_2014_1072_MOESM3_ESM.pdf]

Screening of potentially relevant records, on the basis of full text

30 May 2013

183 records were accepted to the review of full text.

In the process of looking for the full text of these 183 records [reviewer 1] found :

- 15 duplicates
- 26 are not in English - some of these are also duplicates.

This left us with 145 to share:

- [reviewer 1] review the whole set (145)
- [reviewer 2], [reviewer 3], [reviewer 4] to share the whole set (50, 50 and 45 records respectively).

Results

Results of screening process:

Agreement to accept in the review 13 records (9%) and to reject 99 (68%). Reviewers were in doubt about 1 and disagreed on the case of 32 records: both of these (33 records) were referred to third review [reviewer 5].

The third reviewer accepted further 9, but was in doubt over 2.

A meeting was held among all reviewers to discuss the third review of the 33 records and 10 records were accepted, albeit in two cases with the intention to further assess them at the stage of data extraction.

To note:

1 of the 99 records that did not make it into the review was a 2010 edition of a review updated in 2012:

NG, S. Q., J. D. BRAMMER and D. K. CREEDY. 2010. The psychometric properties, feasibility and utility of behavioural observation methods in pain assessment of cognitively impaired elderly people in acute and long-term care: A systematic review. Joanna Briggs Library 2010, 8(24 Suppl). http://www.joannabriggslibrary.org/index.php/jbisrir/article/view/644

Copies were requested as interlibrary loan of both the 2110 and 2012 editions but the supplying library reported that these are duplicates and supplied only the 2012 record. The latter was included in our review.

Table 1. Results of the screening on the basis of full text – 2 reviewers

Screening outcome	Unique records (number)	(%)
Agreement - YES	13	9%
Agreement - NO	99	68%
Agreement - MAYBE	1	1%
Disagreement	32	22%
Total	145	100%

Table 2. Results screening on the basis of full text – 3rd reviewer

Screening outcome	Unique records (number)	(%)
YES	9	27%
NO	22	67%
Maybe	2	0.6%
Total	33	100%

Table 3. Overall results of the screening of full text

Screening outcome	Unique records (number)	(%)
Accepted	23	16%
Rejected	122	84%
Total	145	100%
