# Supplementary material for: Pain assessment for people with dementia: a systematic review of systematic reviews of pain assessment tools
Source: BMC Geriatr. 2014 Dec 17;14:138. doi: 10.1186/1471-2318-14-138 (PMC4289543; doi:10.1186/1471-2318-14-138)
Supplement: Supplementary file 4 — Additional file 4: Data Extraction forms. The data extraction forms used to build a data extraction database and extract data for the meta-review. (PDF 169 KB) [file 12877_2014_1072_MOESM4_ESM.pdf]

Forms for data extraction database

11 June 2013 – revised version

This document contains the data extraction forms used to build a data extraction database and extract data for the meta-review.

Form 1. Review Bibliographic Information

Field	Content
Publication ID	
Author(s)	e.g. Smith, A; White, LB; Red, K.
Title	e.g. The review of pain tools for patients with headaches
Year	e.g. 2013
Journal	
Volume	
Issue	
Pages	
URL	

Form 2. Review Quality Assessment (AMSTAR)

Field	Content
Publication ID	Linking this record to Table 1
Reviewer	
Date	Date this record was last modified by the reviewer – automatic date stamp
**Question 1. Was an 'a priori' design provided?**	☐ Yes<br>☐ No<br>☐ Can't answer<br>☐ Not applicable
**Question 2. Was there duplicate study selection and data extraction?**	☐ Yes<br>☐ No<br>☐ Can't answer<br>☐ Not applicable
**Question 3. Was a comprehensive literature search performed?**	☐ Yes<br>☐ No<br>☐ Can't answer<br>☐ Not applicable
**Question 4. Was the status of publication (i.e. grey literature) used as an inclusion criterion?**	☐ Yes<br>☐ No<br>☐ Can't answer<br>☐ Not applicable
**Question 5. Was a list of studies (included and excluded) provided?**	☐ Yes<br>☐ No<br>☐ Can't answer<br>☐ Not applicable
**Question 6. Were the characteristics of the included studies provided?**	☐ Yes<br>☐ No<br>☐ Can't answer<br>☐ Not applicable
**Question 7. Was the scientific quality of the included studies assessed and documented?**	☐ Yes<br>☐ No<br>☐ Can't answer<br>☐ Not applicable
**Question 8. Was the scientific quality of the included studies**	☐ Yes

**used appropriately in formulating conclusions?**	□ No<br>□ Can't answer<br>□ Not applicable
**Question 9. Were the methods used to combine the findings of studies appropriate?**	□ Yes<br>□ No<br>□ Can't answer<br>□ Not applicable
**Question 10. Was the likelihood of publication bias assessed?**	□ Yes<br>□ No<br>□ Can't answer<br>□ Not applicable
**Question 11. Was the conflict of interest stated?**	□ Yes<br>□ No<br>□ Can't answer<br>□ Not applicable
**Reviewer Notes**	

Form 3. Review content / method

Field	Content
Publication ID	Linking this record to Form 1
Reviewer	
Date	
Aim of Review/Objectives	
Number of included studies	
Search dates	Years covered in the review – e.g. 2000 – 20013
Databases searched	
Country of origin	
Inclusion/exclusion criteria	
Methods of Analysis	
Quality of the studies	What the authors thought about the quality and risk of bias of original studies
Recommendations from authors	
Tools included in the review	List - Link to Form 4
**Reviewer Notes**	

Form 4. Tool Validity/Reliability/Utility Data

Field	Content
Name of Tool	
Publication ID	Linking this record to Form 1
Reviewer	
Date	Date this record was last modified by the reviewer – automatic date stamp
Number of studies	Number of studies used by the Publication to inform the analysis
List of studies	List the studies used as the source for this evaluation
Purpose of tool	Describe purpose of tool as outlined in the review
Background	What review authors suggest was the purpose of the tool and who it was designed to be used by
Description	Describe the tool itself- number of items, what they are designed to measure
User-centredness	What does the review say about where the tool items were generated from
Setting	The setting the tool was designed for and the setting where it was tested or used (if different).
Reliability	Data providing evidence on tool reliability
Validity	Data providing evidence on tool validity
Specificity	Data providing evidence on tool specificity
Sensitivity	Data providing evidence on tool sensitivity
Feasibility	How easy is it to use - how much training is needed?
Clinical Utility	Evidence of clinical utility and whether the tool has been used in/tested in practice
Other comments	Anything else relevant to the study
**Reviewer Notes**	
