# Supplementary material for: Pain assessment for people with dementia: a systematic review of systematic reviews of pain assessment tools
Source: BMC Geriatr. 2014 Dec 17;14:138. doi: 10.1186/1471-2318-14-138 (PMC4289543; doi:10.1186/1471-2318-14-138)
Supplement: Supplementary file 5 — Additional file 5: Summary of reviews methods. Overview of the included reviews, with a description of the aims and the methods used for including and analysing the studies, their quality assessment of the studies, and the tools examined. (DOCX 31 KB) [file 12877_2014_1072_MOESM5_ESM.docx]

Table AF5. Summary of reviews methods - criteria for inclusion, method for analysis, and quality of the studies

Overview of the included reviews, with a description of the aims and the methods used for including and analysing the studies, their quality assessment of the studies, and the tools examined (table cells left empty when no data were available).

| **Review. ID** | **Aim of Review/Objectives** | **Number of included studies** | **Inclusion/exclusion criteria**  **(defined in terms of either instruments or studies’ characteristics)** | **Tools included in the review** | **Methods of Analysis** | **Quality of the studies** |
| --- | --- | --- | --- | --- | --- | --- |
| [22] | To provide a comprehensive overview of current evidence regarding pain in dementia to guide the evidence-based assessment and treatment of pain in individuals with this disease. | 18 | *Criteria defined in terms of the instruments:*  Whether instrument: still available; specifically validated in people with dementia; had good overall quality and clinical utility, based on traffic light rating system by 3 of the reviewers. Traffic light system focused on psychometric properties of scale, time needed for administration, utility, required training for health and care professionals using instrument. No data on how these were used to make decision about inclusion/exclusion. | 12 tools: Abbey Pain Scale; ADD; CNPI; Doloplus-2; DS-DAT; EPCA-2; NOPPAIN; PACSLAC-D; PADE; PAINAD; PAINE; PPI. | Narrative | - |
| [37] | To critically evaluate the existing tools used for pain assessment in nonverbal older adults with dementia to provide recommendations to clinicians. | - | *Criteria defined in terms of the instruments:*  To be included in this review, assessment tools met the following criteria: (1) based on behavioral indicators of pain, (2) developed for assessment of pain in nonverbal older adults with severe dementia or evaluated for use with nonverbal older adults, (3) available in English, and (4) at least one published research report of psychometric evaluation available in English. | 10 tools: Abbey Pain Scale; ADD protocol; CNPI; DS-DAT; Doloplus 2; FLACC; NOPPAIN; PACSLAC; PADE; PAINAD. | A critique guide was developed based on measurement theory with criteria and indicators in five areas: conceptualization, subjects, administration, scoring and feasibility, and reliability and validity. Geriatric pain literature was consulted to establish a framework for evaluation of content and comprehensiveness of nonverbal pain indicators (content analysis of tools).  Each tool was critiqued independently by each of the three authors and rated for evidence that supported the criteria and indicators as defined by using a 4-point scale (3 = available evidence is strong, 2 = available evidence supports need for further testing, 1 = available evidence is insufficient and/or tool revisions are needed, and 0 = evidence is absent). Ratings were then compared and discussed in detail until consensus was reached for each criterion. The preliminary critique was mailed to the tool developer for review for accuracy and submission of additional data or publications was invited. When new data were submitted tool critiques were discussed by the review team members and adjustments made when appropriate. A final score was assigned based on the available data | Assessed |
| [43] | Not clear - to provide overview of literature on pain assessment in older adults with advanced dementia, and overview of assessment methods (?) | - | - | 8 tools: ADD; CNPI; DS-DAT; Comfort checklist; Observed Pain Behavior Scale; PADE; PAINAD; PPQ. | None given, appears to be narrative overview. | - |
| [41] | To identify key components involved in behavioral pain assessment in cognitively impaired elderly people; to analyse the reported pscyhometric properties (reliability, validity, sensitivity, specificity) feasibility & utility of behavioral pain assessment tool. | 23 | *Criteria defined in terms of the studies:*  Studies: any study design - purpose to assesse reliability, validity, sensitivity, specificity, feasilibity, utility of tools used in behavioral pain assessment; Participants: institutionalised elderly people over 65 years of age defined as cognitively impaired, elderly people unable to communicate, conducted in acute care, aged care or nursing home settings;  Outcomes: identification of behavioral criteria for assessment of pain, development of behavioral pain assessment tool, evaluation of the use of existing behavioral pain assessment tool, investigation of any aspect of psychometric properties, feasibility or utility of tool;  Exclusion: published in language other than English, where administration of tool not by nurses or nursing assistants, studies looking at tools for children or critically ill sedated hospitalised patients. | 10 tools: Abbey Pain Scale, CNPI; CPAT; Doloplus-2; Mahoney Pain Scale; MOBID; NOPPAIN; PACSLAC; PAINAD; REPOS. | Provision of narrative summary for each tool.  Quantitative studies reviewed for psychometric properties, feasibility and utility data. Statistical pooling of data not possible so categorised according to assessment tool and critiqued. | Studies showed considerable heterogeneity in terms of methods.  (Review did not provide an overview of assessment of quality). |
| [42] | To evaluate the existing observational scales for assessing pain among older adults with severe dementia or cognitive impairments who are unable to report their pain. In particular, to examine whether each scale has acceptable reliability and validity scores and clinical utility.  Focus of review: pain assessment by social workers / nursing home settings. | 21 | *Criteria defined in terms of the instruments:*  To be included in this review, assessment tools met the following criteria:  (1) included behavioral or (and) emotional indicators of pain,  (2) assessed pain in older adults with severe dementia or cognitive impairments that prevent them from reporting their pain,  (3) had an English version available, (4) had been published between 1990 and 2007 (5) had been used in nursing homes or long-term care units in hospitals. | 11 tools: Abbey Pain Scale; ADD; Behavior Checklist; CNPI; DS-DAT; MOBID; NOPPAIN; PACSLAC; PADE; PAINAD; PATCOA. | - | Major methodological limitations pertaining to the reviewed scales identified. |
| [27] | To present the findings of a literature review related to pain in the older adult. Emphasis on the use of assessment in adults with cognitive impairment. | 9 | *Criteria defined in terms of the studies:*  Population: individuals between ages of 60-100.  Interventions: included pharmacologic, non-pharmacologic, assessment methods, complementary approaches.  Outcomes: clinical outcomes of interventions such as quality of life or depression and socioeconomic information.  Study design: all designs; excluded articles that were not research based, related to chronic pain and/or older people. | 9 tools: Abbey Pain Scale; ADD; CNPI; Doloplus-2; DS-DAT; NOPAIN; PADE; PAINAD; PACSLAC. | Narrative analysis | No reported assessment of the quality of studies - though highlighted the limitations in studies’ approach to evaluating tool reliability and validity. |
| [44] | To review the pain observation scales used in or developed for older adults with severe cognitive impairments, communication difficulties or both. | - | *Criteria defined in terms of the instruments:*  Inclusion: pain observation scale used in an empirical study, psychometric properties reported in older adults with severe cognitive impairments, communication difficulties or both, or if a scale had been developed specifically for use in older adults.  Exclusion:- pain observation scales developed specifically for children and critically ill sedated hospitalized patients; non-English articles (unless an English abstract available) | 13 tools: Abbey Pain Scale; ADD; Behavior checklist; CNPI; Doloplus-2; DS-DAT;FACS; NOPAIN; PACSLAC; PADE; PAINAD; PATCOA; PBM. | No data on this - appears to be a narrative synthesis | No data on this - discussed robustness of studies testing of reliability and validity of the tool. |
| [21] | To identify behavioural pain assessment tools available to assess pain in elderly people with dementia and the psychometric qualities and clinical utility of these tools. | 29 | *Criteria defined (mainly) in terms of the instruments:*  To be included in this review, assessment tools to meet the following criteria:  (1) assessment instrument/ scale for elderly patients with dementia or a subgroup of elderly patients with dementia, for example, Alzheimer patients. (2) The assessment scale had to have been used to measure pain by means of self-reports by patients or behavioural measures. (3) Publications had to be in English, Dutch, German or French. (4) Publications had to be other than case reports or secondary sources/reviews | 12 tools: DOLOPLUS2;  ECPA; ECS; Observational Pain Behavior Tool; CNPI;  PACSLAC; PAINAD; PADE; RaPID; Abbey Pain Scale; NOPPAIN; Pain Assessment Tool for Use with Cognitive Impaired Adults. | Data abstraction criteria used to evaluate behavioural assessment scales were defined prior to assessment (partly based on Streiner & Norman's requirements for health measurement scales, based on quality judgement criteria relating to validity, reliability and homogeneity)  A small part of the data abstraction process (N = 3 articles) was conducted by two reviewers, as a quality check (with overall agreement found to be 90%). | The authors commented on both the quality of the scales and the quality of the studies. Heterogeneity in terms of study design was found. On the quality of the scales, their overall assessment is “generally moderate”. Only 4 of the 12 scales scored 11 points out of 20. They noted “that most of the scales are still under construction, especially with regard to criterion and construct validity.”[21] |
