# Supplementary material for: Pain assessment for people with dementia: a systematic review of systematic reviews of pain assessment tools
Source: BMC Geriatr. 2014 Dec 17;14:138. doi: 10.1186/1471-2318-14-138 (PMC4289543; doi:10.1186/1471-2318-14-138)
Supplement: Supplementary file 6 — Additional file 6: Sample of studies methodological weaknesses identified by the reviews. A sample of studies’ methodological challenges and weaknesses identified in/by the reviews, organised by type of issue (e.g. sampling, tool design). (DOCX 24 KB) [file 12877_2014_1072_MOESM6_ESM.docx]

**Table AF6. Summary of studies’ methodological issues identified by the reviews**

This table provides a sample of studies’ methodological challenges and weaknesses identified in/by the reviews, organised by type of issue (e.g. sampling, tool design).

| **ID** | **Absence or inappropriate comparison scale for assessment of validity** | **Sampling (sample size, type of pain, measure of cognitive impairment)** | **Tool design (e.g. indicators of pain used)** | **Studies methodological weaknesses** |
| --- | --- | --- | --- | --- |
| [37] | Clinicians are eager for a tool that can objectively score pain behaviors to determine intensity of pain. But because there is no gold standard available for validating presence of pain in nonverbal older adults establishing validity of tools that measure intensity is very difficult. | There is considerable variability between patients with dementia in their expression of pain via behavioral demonstration; different patterns of behavior could both indicate the presence of pain, but not be represented easily in a tool to quantify pain related behaviour. | Tools that assess a broad range of possible pain behaviors may have greater clinical utility and capture those pain responses that are less obvious. They would have greater sensitivity. However, many subtle indicators, such as mental status change, are not exclusive to pain. Thus, they may increase the likelihood of identifying pain when it is not present (false positives) and thus result in decreased specificity. Research has not yet established the sensitivity of presence of individual behaviors as indicators of pain. | There is evidence to support ability of providers and caregivers to accurately identify presence of pain, but not Intensity.  The individual variability in pain presentation again impacts the ability to capture intensity in a manner that reliably reflects the diversity of individual presentations. |
| [42] | No widely accepted and empirically validated gold standard scale for measuring behavioral and emotional (e.g., facial expression) components of pain in nonverbal older adults with cognitive impairments or dementia. | Convenience sampling, relatively small sample sizes. | Some of the scales do not include all six indicators in the AGS guidelines.  Scales appear to be limited in the range of emotional and behavioral components. Some do not provide scoring methods or interpretations of scores measured. | None of the scales has been extensively tested in a range of residential care settings by a range of clinicians. |
| [44] |  |  | Need to develop cut off scores for scales to indicate whether or not to provide interventions for pain - need to link assessment with treatment algorithms. |  |
| [21] | To assess validity, instruments were correlated with a VAS or alternative intensity scale filled in by a proxy. The legitimacy of this approach is questionable. | Small samples of participants or a limited number of pain situations for (at times) large number of items in the scale. | Concerns about indicators collected to construct pain scales. These may be influenced by the type of pain focused on the setting in which indicators are collected.  Heterogeneous items in the scale: overlapping items might be most common and important ones, while unusual items might be more characteristic of the target group but less useful for a general pain scale for elderly people with dementia. | Not enough information about the frequency of endorsement of certain items in the population examined, or the importance of items at rest and during a painful situation can affect results.  Most studies lack information on sensitivity and specificity, and without this information, a scale is useless for clinical practice. |
