# Supplementary material for: Pain assessment for people with dementia: a systematic review of systematic reviews of pain assessment tools
Source: BMC Geriatr. 2014 Dec 17;14:138. doi: 10.1186/1471-2318-14-138 (PMC4289543; doi:10.1186/1471-2318-14-138)
Supplement: Supplementary file 7 — Additional file 7: Summary of the tools - characteristics. Summary of the data extracted from the reviews providing a classification and description of the tools. (DOCX 74 KB) [file 12877_2014_1072_MOESM7_ESM.docx]

**Table AF7. Summary of the tools: characteristics**

Summary of the data extracted from the reviews providing a classification and description of the tools (table cells left empty when no data were available).

| **Review ID** | **Name of tool** | **Classified as** | **Tool format** | **Items** | **Measurement** | **Application/Interpretation** | **Origin** |
| --- | --- | --- | --- | --- | --- | --- | --- |
| [21] [22] [27] [37] [41] [42] [44] | **Abbey Pain Scale** | All but one of the reviewers who included the Abbey Pain Scale in their reviews classified the instrument as an observational rating scale based on the healthcare professional’s observation and interpretation.  However, one review classified the Abbey Pain Scale as being a caregiver/informant rating scale. In doing this the reviewers suggest that the person undertaking the rating should have “at least daily contact with the patient”. | All the reviewers identified the Abbey Pain Scale as using a six item scale.  Three of the reviewers also described the tool as being a brief assessment scale for people with end-stage dementia. | The reviewers identified the six items as being:   1. vocalization 2. facial expression 3. change in body language 4. behavioural change 5. physiological change 6. physical change. | One review indicated that the Abbey Pain Scale can be used by a variety of care staff, whereas three reviews appeared to suggest nurses using the pain scale.  The other two reviewers either did not discuss who would use the Abbey Pain Scale, or use the generic term “staff”.  The authors indicated that each item is levelled on a 4-point scale for intensity of the behaviour (absent = 0, mild = 1, moderate = 2, severe = 3) with total score ranging from 0 to 18. However, judging from the explanation of the levelling scale, it would appear that a 3-point scale is used.  One review did not provide detailed information on measurement of the items, while two reviews indicated that the cut-off scores are based on a cross-tabulation of the Abbey Pain Scale scores against holistic pain measures by the participating nurses (named holistic measure). | The total score is interpreted as intensity of pain: no pain = 0--2, mild = 3--7, moderate = 8--13, and severe = 14-18. The type of pain (acute, chronic, acute on chronic) is noted.  Two reviews did not provide detailed information on interpretation of the score.  Two reviews indicated in their reviews that clear scoring instructions and detailed item definitions are lacking. This was not identified by the other authors. | Australia |
| [27] [37] [43] [42] [44] | **ADD Protocol** | One review described the ADD Protocol as an interactive rating scale. In using this scale, physical and affective needs assessment, review of the patient’s history and the administration of analgesics are included in the assessment.  Other reviewers did not explicitly classify the ADD Protocol. However, their description of the instrument’s use suggested a multi-facetted approach that would be in keeping with an interactive rating scale. | A protocol of five categories of pain behaviours with dichotomous items specified within each category Is used to identify potential pain behaviours.  One review identified physical assessment, history review and assessment of affective needs as part of the ADD Protocol. | One review suggested that the behavioural symptoms in the protocol mirror the six AGS pain indicators (physiological indicators, facial expression, motor behaviour, social behaviour or mood, vocalization, eating or sleeping pattern).  One review named six, slightly different, categories: facial expression, mood, body language, voice, behaviour and other. Whereas one review named five categories and indicated the number of items in each: facial expression (8 items), mood (5 items), body language (9 items), voice (9 items) and behaviour (11 items).  One review provided a more descriptive set of behaviours: Tense body language and repetitive movement, fidgeting, physical aggression, tearfulness, delusions, withdrawal behaviour, sad or frightened facial expression, verbal outburst, repetitive walking at night, phobias or fears, hallucinations, noise breathing. | Two reviews clearly identified nurses as using the ADD Protocol.  Four reviews did not discuss who would use the ADD Protocol  Two reviews described the ADD Protocol as having five steps:   1. physical assessment 2. review of history for potentially painful conditions and use of interventions related to the physical assessment findings 3. affective assessment and implementation of non-pharmacologic comfort measures 4. use of a prescribed, as needed non-opioid analgesic if behaviours persist 5. consultation with the physician or other health provider if behaviours persist, or use of a prescribed, as needed psychotropic drug.   Four reviews provided no detail on the operationalization of the ADD Protocol. | Two reviews highlighted that changes in facial expression, mood, body language, voice, and behaviour are used as cues to apply the ADD Protocol.  Two reviews stated that changes in behaviour and interpretation of the rating scale is based on the assumption that behavioural change in patients with dementia occurs when basic needs interventions have failed to provide comfort. | USA |
| [42] [44] | **Behavior checklist** | Two reviews both classified the Behavior Checklist as an Observational rating scale. | The checklist consists of twenty items. | The Behavior Checklist includes twenty items such as moaning, quiet, crying easily, rocking, agitated, or withdrawn. | Neither review included information on who would use the Behavior Checklist.  The Checklist measures absence or presence of pain.  One review indicated that the Behavior Checklist does not measure pain frequency or intensity. | - | USA |
| [21] [22] [27] [37] [43] [41] [42] [44] | **CNPI** | All the reviewers classified the instrument as an observational rating scale. | The CNPI consists of six items. | Six of the eight reviews provided details of the six pain behavioural items commonly included in the CNPI:   1. nonverbal vocalizations 2. facial grimacing/wincing 3. bracing 4. rubbing/massaging 5. restlessness 6. vocal complaints   Two reviews provided no detail on the items included in the checklist. | Three reviews suggested that the checklist is easy to use, but did not explicitly indicate who should use the CNPI. Three reviews provided no information on who should use the CNPI.  One review was not explicit, but appeared to suggest nurses using the CNPI. In contrast, another suggested that staff nurses would use the checklist in practice.  Each item is scored on a dichotomous scale (1 = present, 0 = not present) at rest and on movement.  One review also identified a dichotomous “yes/no” scale, but added that behaviour is measured after a 5 minute observation period.  One review provided no information on the checklist’s application. | Two reviews indicated that an item is scored as '1' if the behaviour was observed during activity or rest and as '0' if the behaviour was not observed (range of total scale 0–6). After adding up the two scores (for movement and rest) the interpretation is as follows: '1–2' mild pain, '3–4' moderate pain, '5–6' severe pain.  Scoring involves patient observation at rest and during movement and the dichotomous score has a possible range of 0 to 6 points for each situation and a maximum of 12 points..  Two reviews provided no information on the practical use or interpretation of the checklist.  Two reviews indicated that the method of administration and scoring instructions are clear. However, these authors went on to suggest that interpretation of the checklist score is unclear and that no further instructions are provided following the assessment.  One review raised concern that the cut off scores ('1–2' mild pain, '3–4' moderate pain, '5–6' severe pain) are not developed. | USA |
| [43] | **Comfort Checklist** | The Comfort Checklist was classified as being an observational rating scale. | The Checklist contains five domains of assessment. | The five domains in the Comfort Checklist were:   1. vocalization 2. motor signs 3. behavioural indicators 4. facial expressions 5. miscellaneous symptoms. | The review included no information on who would use the Comfort Checklist.  Specific signs are specified for each domain with a suggested rating from none, to moderate, to severe. | The checklist does not assign numeric rating of discomfort to the measurement; instead it indicates the complex and multifactorial assessment that may be needed to assure comfort among non-communicative patients with Alzheimer’s disease. | USA |
| [41] | **CPAT** | The review classified the CPAT as an observational rating scale. | The tool consisted of observational pain behaviour indicators in five categories. | The five categories of the CPAT were identified as:   1. facial expression 2. behaviour 3. mood 4. body language 5. activity. | The review indicated that the CPAT is designed for use by Certified Nurse Assistants.  Pain indicators are scored as 1 and non-pain indicators are scored as 0 with a maximum total possible score of 5. | The review indicated that the method of administration and scoring instructions are clear, but that interpretation of the tool score is unclear and no further instructions are provided following the assessment. | USA |
| [21] [22] [27] [37] [41] [44] | **Doloplus-2** | All the included reviews categorised the Doloplus-2. As being an observational rating scale. | Doloplus-2 consists of three dimensions/sub-groups and a total of ten items (situations). | The reviewers identified the three dimensions contained in the Doloplus-2:   1. somatic reactions (5 items) 2. psychomotor reactions (2 items) 3. psychosocial reactions (3 items)   Two reviews explained the items more fully as being somatic complaints, body posture, protection of sore areas, expression, sleep patterns, washing and dressing, communication, social life and behaviour problems. | One review included no information on who would use the Doloplus-2.  Two reviews were not explicit, but appeared to suggest nurses using the Doloplus-2.  However, one review indicated the preference for multidisciplinary scoring when using Doloplus-2.  Each item has four behavioural descriptions, representing increasing intensity of pain rated from 0 to 3. Individual item scores are summed to arrive at a total score ranging from 0 to 30 points.  Observations of patient behaviour in ten different situations (10 items/3 dimensions) that could reveal pain. | A score greater than or equal to five out of 30 (maximum pain score) confirms pain. However, the reviewers argued that pain cannot be ruled out if the older adult has less than five points.  One review stated that the DOLOPLUS2 score does not represent pain experience at a specific moment but reflects on the progression of experienced pain.  One review indicated that the method of administration and scoring instructions are clear.  Three reviews indicated that the cut off score of 5 is not validated. | France |
| [22] [27] [37] [43] [42] [44] | **DS-DAT** | Four reviews clearly identified the DS-DAT as an observational rating scale.  One review suggested a classification of an observational rating scale, but are not explicit.  One review did not classify the instrument. | The DS-DAT consists of nine items; rating pain frequency, intensity and duration.  Two reviews provided no information on the tool format. | Nine items were identified by most of the authors who included the DS-DAT in their review:   1. noisy breathing 2. negative vocalizations 3. content facial expression 4. sad facial expression 5. frightened facial expression 6. frown 7. relaxed body language 8. tense body language 9. fidgeting.   One review provided no information on the tool items. | One review indicated that the DS-DAT is designed to be used by an observer not familiar with the patient, and appears to suggest that nursing staff would use the instrument.  Five reviews provided no information on who would use the DS-DAT.  Each of the nine items are measured after a 5-minute observation, according to the following three variables.  **Frequency:** each of the 9 items during a 5-minute observation period.  One review stated the observation should take place a minimum of 30 minutes after an intervention, while Herr et al (2006) stated a minimum of 15 minutes after a discomfort period and a minimum 5-min rest period.  **Intensity**:   - low intensity = barely to moderately perceptible - high intensity = present in moderate to great magnitude.   **Duration**:   - short duration < 1 min - long duration > 1 min.   Two reviews provided no information on the tool’s application. | One review stated that the DS-DAT tool focusses specifically on discomfort rather than pain.  The nine items are scored for absence or presence of discomfort, which, if present, is scored for frequency, duration, and intensity. Each item may achieve a score of 0-3 points yielding a total score from 0 = no observed discomfort to 27 = high level of observed discomfort.  One review indicated that sensitivity to change and cut off scores are not developed. | UK |
| [21] | **ECPA** | Observational rating scale. | Eleven items in three dimensions. | Eleven items in the three dimensions :   1. pre-care 2. post-care 3. during activities. | The review included no information on who would use the ECPA.  The review indicated that the scale consists of eleven items with *five response modalities scored from 0 (no pain) to 4 (absolute pain)*, but then provide the example of:  0= relaxed face  1= concerned face  2= face sometimes grimacing  3= frightened, face contorted with pain.  With a total score range from 0 (no pain) to 44 (absolute pain) which suggests that there are *four* response modalities, rather than five. | The range of scores from 0 to 44 represents increasing degrees of pain. | France |
| [21] | **ECS** | Observational rating scale. | Ten items that are scored on three, four or five levels, depending on the item. | Example of items:   1. sleep 2. verbal reaction 3. interaction with the environment   Further detail of the items is not provided. | The review included no information on who would use the ECS.  The first six items are assessed after care, while items 7 and 8 are assessed during care, and items 9 and 10 every 24 hours. Scoring range 0–14. | - | Canada |
| [22] | **EPCA-2** | Observational rating scale. | Eight item scale. | Eight item scale in two subscales. | The review provided no information on who would use the EPCA-2.  Intensity rated on a five point scale; assessment after 5 min observation before and during care-giving. | - | France |
| [44] | **FACS** | Observational rating scale. | Forty-six items. | The FACS identified forty-six discrete facial action units (AUs) involving specific muscles, such as brow raise, lip stretch or mouth stretch. | Two reviews provided no information on who would use the FACS. Measurement is made by noting the frequency and intensity of facial action units. | One review indicated that facial expressions are interpreted to indicate pain. | USA |
| [37] | **FLACC** | The review described the FLACC as a behavioural scale, but implied that the behaviour would be measured using an observational rating scale. | Five items. | The five items included in the FLACC are:   1. face 2. legs 3. activity 4. cry 5. consolability | The review included no information on who would use the FLACC.  Items levelled on a 3-point scale for intensity by behavioural descriptors for a total score range from 0 to 10. | No information on the interpretation of the measurement was included in the reviews.  Consolability appears more a response to an intervention, rather than a pain assessment. Criteria for measuring the behavioural descriptors have not been validated for adults with dementia (FLACC was developed for use with children). | USA |
| [41] | **Mahoney Pain Scale** | Observational rating scale. | Eight items divided into two subscales. | Subscale 1 (items 1-4 assessing four pain behaviours):   1. facial expression 2. vocalisations 3. body languages 4. breathing changes   Subscale 2 (items 5-8 differentiating pain from agitation). | The review appeared to suggest nurses using the MPS.  Items are rated on a 4-point scale (0=minimal pain and 3=severe pain). | Scores are summated to estimate pain severity (little pain, mild, moderate or severe pain).  Method of administration, scoring instruction and interpretation of tool score are clear. | Australia |
| [22] [41] [42] | **MOBID** | Observational rating scale. | A two-part tool assessing pain intensity based on observed pain behaviour after guided movements  One review did not identify the MOBID-2 as being a two-part tool. | Part 1: musculoskeletal (five items).  Part 2: head, skin and internal organs  One review identified the five musculoskeletal items in the Part 1 protocol as being:   1. mobilization of the hand 2. mobilization of the arms 3. mobilization of the legs 4. turn over in bed 5. sitting on bedside (at first). | Two reviews were clear that nurses use the MOBID-2.  PART 1: The nurse moves the patient through the protocol of five movements and indicates when pain behaviour indicators are observed.  The nurse then rates the intensity of each pain behaviour on a 0-10 point Numerical Rating Scale (NRS).  PART 2: The nurse records the localisation of pain on body outline diagrams. The inferred pain intensity originating from internal organs for each item is rated on the NRS.  After completion of the whole protocol, an overall pain intensity is completed, again using the NRS. | No information on the interpretation of the measurement was included in the reviews.  Method of administration is clear.  Scoring instructions are unclear as no criteria were established to determine the intensity of pain ratings. | Norway |
| [21] [22] [27] [37] [41] [42] [44] | **NOPPAIN** | The NOPAIN was categorised as an observational rating scale in most of the reviews.  One review did not specifically state how this tool is categorised. | The NOPAIN contains four main sections and six items.  One review indicated that the NOPAIN contains nine activities of daily living.  In contrast, one review indicated that the tool contains 17 items. No further detail is provided. | The NOPAIN is used while doing common care tasks. Six specific pain behaviours (pain words, pain noises, pain faces, bracing, rubbing and restlessness) are incorporated in the four main sections/ components.  Five reviews identified the NOPAIN tool as having four main sections/ components:   1. Observed pain in response to activities of daily living (bathing, dressing, transfers, etc.) 2. Presence/absence of pain behaviours: vocalization and facial expression (pain response) 3. bracing and restlessness (pain location) 4. a global rating of pain for that day (pain thermometer).   Although one review also identified four components in the tool, they name these as being:   1. Care conditions under which pain behaviour is observed. 2. The presence/absence of pain behaviours (pain faces, pain words, pain noises, bracing, rubbing and restlessness). 3. Pain intensity rated on a 6-point Likert scale. 4. Overall pain intensity rated on a pain thermometer.   One review explained the NOPAIN is divided into four sections;  1. Carer is asked to provide information regarding care being delivered at time of assessment.  2. Then the caregiver is provided with the six specific pain behaviours and asked to state if they were identified during care.  3. The carer then scores them on a scale of 1-5 depicting intensity.  4. Lastly, the carer rates the overall pain intensity for the day using a pain thermometer. | One review indicated that “caregivers” would use this instrument, while three reviews indicated that the NOPPAIN is designed for use by Nursing Assistants.  Pain is assessed at rest and with movement. After patients are observed during care activity, the caregiver completes the NOPPAIN. Pain behaviour intensity ratings using a 6-point Likert scale; and a pain thermometer for rating overall pain intensity are noted.  Two reviews described the scoring as combining information about pain behaviour, care conditions and a Likert-type scale of pain intensity.  One review indicated a score range of 0-30.  One review is the only review that identified that the NOPAIN contains pictures to facilitate use for caregivers with poor English language skills. | Method of administration was reported as clear. However, one review reported that cut off scores were not developed, and two reviews that no criteria are set for measuring intensity and the interpretation of scores is unclear.  One review also indicated that there is no indication how to proceed after the items in the NOPAIN instrument have been rated. | USA |
| [21] [43] | **Observational Pain Behaviour Tool** | Both authors classified the Observational Pain Behaviour Tool as an Observational rating scale. | Seven domains (one review)/seven dimensions (one review) containing twenty-five items. | Twenty-five items (observable pain behaviours) in the Observational Pain Behaviour Tool are divided among seven domains. One review indicated that the number of verbal descriptions per domain ranges from three to eight and are not mutually exclusive categories.  One review provided more detail regarding the spread of items across the dimensions:   1. verbal response (N = 8) 2. facial expression (N = 3) 3. body language (N = 5) 4. conscious state (N = 3) 5. physiological change (N = 3) 6. behavioural change (N = 1) 7. feedback from others (N = 2). | One review indicated that the Observational Pain Behaviour Tool can be used by carers without extensive prior knowledge of the patient. However, the reviewers are not explicit as to who is meant to use the instrument.  The tool consists of a data sheet, a pain assessment chart and a menu of observable pain behaviours (N = 25) that are to be recorded. Scoring range 0–25.  Each item is rated by selecting the verbal description that best fits the patient’s presentation. Scoring is based on recording the behaviour on the sheet as being present at a certain moment and does not include information on pain intensity. | One review provided no information on the interpretation of the measurement. | UK |
| [21] [22] [27] [37] [41] [42] [44] | **PACSLAC** | The PACSLC was categorised as an observational rating scale in the included reviews  Two reviews provided no categorisation for the PACSLC.  A variation - PACSLC-D - was categorised as an observational rating scale in one review | The PACSLC consists of sixty items in four dichotomous subscales based on pain behaviour.  A variation - PACSLC-D consists of 24 items (one review). | A checklist with four subscales and a total of 60 items:   1. facial expressions (N=13) 2. activity/body movements (N=20) 3. social/personality/mood (N=12) 4. physiological indicators (eating and sleeping changes, vocal behaviours) (N=15).   Three reviews identified the four subscales, while one review also indicated the number of items in each subscale.  Similarly, one review identified a checklist with four subscales and a total of 60 items, but use a different terminology:   1. facial expressions 2. activity/body movements 3. Social/personality/mood 4. Other (appetite and sleep patterns).   While one review illustrated a checklist with five subscales:   1. activity/body movements 2. facial expressions 3. vocal behaviours 4. social/personality/mood 5. eating and sleeping.   In PACSLC-D - the items are divided between three dichotomous subscales (one review). | Five reviews provided no information as to who would use the PADE.  Two reviews appeared to suggest nurses using the pain scale.  Each item is scored on a dichotomous scale (present or absent). | Subscale scores are derived from counting the checkmarks (present or absent) in each column to arrive at a total score ranging from 0 to 60.  The number of observed pain behaviours indicate pain intensity.  Two reviews indicated that the method of administration and scoring instructions are clear. However, three reviews noted that interpretation of total score is not provided  One review stated that the cut off score is not validated.  In PACSLC-D - the number of observed pain behaviours indicates pain intensity (one review). | Canada, Netherlands |
| [21] [22] [27] [37] [43] [42] [44] | **PADE** | While six reviews did not specifically classify the PADE, their descriptions seem to suggest this this an observational rating scale.  One review classified the PADE as being a caregiver/ informant rating scale. In doing it was suggested that the person undertaking the rating should have “at least daily contact with the patient”. | The PADE consists of a twenty-four item scale in three parts. | Most of the reviews indicated that the tool PADE has three parts with a total of twenty-four items.  Part I: physical (includes observable facial expression, breathing pattern, and posture).  Part II: global assessment (involves proxy evaluation of pain intensity).  Part III: functional (includes activities of daily living including dressing, feeding oneself, and transfers from wheelchair to bed).  One review provided detail of the twenty-four items.  **Physical signs**  1. “Sad” facial expression  2. “Anxious/frightened” facial  expression  3. Frowning  4. “Tense” body language  5. Restlessness  6. Grimacing, bracing, groaning during transfers  7. Guarding affected area  8. Moaning, groaning  9. Distress speech, vocalizations  10. Language coherence, complexity  11. Loud, gasping breathing  12. Rapid breathing, hyperventilation  13. Grooming, overall appearance  **Global assessment**  14. Caregiver rating of resident’s pain level  **Functional abilities**  15. Independence in eating  16. Amount eaten  17. Location of meal  18. Time awake in 24 hours  19. Hours out of bed while awake  20. Hours interacting with others  21. Cooperation during personal care  22. Level of agitation  23. Wandering, pacing fidgeting  24. Indications of wanting to leave facility. | Five reviews provided no information as to who would use the PADE. One review appeared to suggest use of the PADE by nurses and indicates that an observer who knows the patient well would achieve a more accurate assessment, while one review referred to a generic group of caregivers.  Different anchors are used in the tool: The twenty-four item scale in three parts; including VAS items; Likert scales 1-4, narrative descriptions of pain experienced over the previous two weeks obtained from the documentation (three reviews).  One review indicated that some items are scored retrospectively, others are not e.g. items are rated on a VAS after 5 min of observation. In addition, chart documentation data concerning the last 24 hours are recorded. However one review also indicated that the twenty-four item are recorded in 4-point scale and using multiple choice.  Smith (2005 is more explicit and indicates that fifteen items are scored on a 1 to 4 scale using description to anchor the item scoring (e.g., none to extreme; calm to extremely restless). The remaining nine items offer four categorical choices that are also scored from 1 to 4 in response to a question.  One review indicated that all twenty-four items are scored on a VAS after 5 min of observation. | One review indicated that the PAINAD is based on an assumption that caregivers can reliably rate the intensity of an older adult’s pain.  One review highlighted that the score range is 24-96 and one review suggested that throughout the scale, lower scores are associated with more functional behaviour and higher scores are associated with dysfunctional or distressed behaviours.  Three reviews indicated that interpretation of the measurement is unclear (partly due to the different scoring methods).  Cut off score not available (two reviews). | USA |
| [21] | **Pain assessment scale for use with cognitively impaired adults** | Self-reporting and observational rating scale. | Eleven sections/parts. | The multidimensional Pain assessment scale for use with cognitively impaired adults includes the following sections/parts:   1. existing painful conditions 2. physiological measures 3. self-report of pain (vocalisation) 4. facial expression 5. usual behaviours (activities of daily living) 6. changes in behaviours 7. body posture 8. cognitive functioning 9. physical changes 10. usual comfort measures 11. new comfort measures. | The review indicated that the Pain assessment scale for use with cognitively impaired adults is designed for use by Nursing Assistants.  The review indicated that the items/sections are rated using different scoring methods. | - | Australia |
| [21] [22] [27] [37] [43] [41] [42] [44] | **PAINAD** | The reviewers agreed that the PAINAD is categorised as an observational rating scale. | The PAINAD consists of five items. | Five items:   1. breathing, 2. negative vocalization, 3. facial expression, 4. body language 5. consolability.   One review identified the five items/domains in the PAINAD (see above), but also provided specific descriptions for each level of discomfort:  **Breathing: independent**  **of vocalization**  0 = Normal  1 = Occasional laboured breathing; short period of  hyperventilation  3 = Noisy laboured breathing; long period of  hyperventilation; Cheyne-Stokes respirations  **Negative vocalization**  0 = None  1 = Occasional moan or groan; low level of speech with negative or disapproving  Quality.  2 = Repeated troubled calling out; loud moaning or  groaning; crying  **Facial expression**  0 = Smiling or Inexpressive  1 = Sad; frightened; frown  2 = Facial grimacing  **Body language**  0 = Relaxed  1 = Tense; distressed, pacing; fidgeting  2 = Rigid; fists clenched; knees pulled up; pulling  or pushing away; striking out  **Consolability**  0 = No need to console  1 = Distracted or reassured by voice or touch  3 = Unable to console, distract, or reassure. | One review appeared to suggest nurses using the pain scale.  However, the remaining reviewers included no information on who would use the PAINAD.  Each item measured on a 3-point scale is rated 0, 1, or 2. One review also identified the use of 3-point scales, but added that the assessment is undertaken after a 5-minute observation period.  Total scores range from 0 for no pain, to 10 for maximum pain.  (Examples of response modalities included in the ‘facial expression item are:  0 = smiling,  1 = sad, frightened or frowning,  3 = facial grimacing). | One review indicated that the method of administration, scoring instruction and interpretation of the tool score are clear.  One review indicated that the PAINAD is based on an assumption that caregivers can reliably rate the intensity of an older adult’s pain. They indicated in their review that the interpretation guidelines are unclear and that no specific length of observation is recommended (5min observation was used during pilot study).  Two reviews stated that cut off scores are not reported. | USA |
| [22] | **PAINE** | The review indicated the PAINE as being a caregiver/informant rating scale. In doing this the reviewers suggest that the person undertaking the rating should have “at least daily contact with the patient”. | The tool consists of twenty-two items. | The twenty-two items include pain-related behaviour such as:   - facial expressions - verbalizations - body movements - changes in activity patterns or routines. | The review indicated that the PAINE is designed to be used by an observer who knows the patient well, but the authors are not explicit as to who would use the instrument.  The review indicated that assessment is done on a seven-point frequency scale over previous 2 weeks. | - | USA |
| [42] [44] | **PATCOA** | Observational rating scale. | Nine pain indicators in four categories. | Four categories:   1. vocalizations 2. behaviours 3. motor activities 4. facial expressions. | The reviews included no information on who would use the PATCOA).  Scores range from 0 to 9. Total score is the number of items present.  Tool measures absence or presence of pain. | No information was included in the reviews with regard to the interpretation of the measurements.  One review indicated that cut off scores were not reported. | USA |
| [44] | **PBM** | Observational rating scale. | The PBM contains five pain behaviours. | The five pain behaviours are:   1. guarding 2. bracing 3. rubbing 4. grimacing 5. sighing. | The review included no information on who would use the PBM.  Besides indicating that the five pain behaviours are scored for the presence or absence of each behaviour, the review provided no information on the practical use of the instrument. | - | USA |
| [22] | **PPI** | Self-Report rating scale. | One item indicating pain experience was suggested by the review, but this is not explicit. | In the body of the review it is indicated the PPI as consisting of an eight-point scale. However, in the supplementary table the scale was identified as being a six-point scale. | The review included no information on who would use the PPI.  Comparison of how pain is experienced currently with how pain was experienced the previous week. |  | Canada |
| [43] | **PPQ** | Observational rating scale. | The PPQ consists of three questions. | The three questions are about:   1. presence (i.e., “Within the last week has the resident experienced pain?”) 2. frequency (i.e., “How often does the resident experience pain?”) 3. intensity (i.e., “When this resident has pain, how would you describe the extent of the pain?”). | The review indicated that the PPQ is designed to be used by an observer who knows the patient well familiar with the patient, and appears to suggest that nursing staff/nursing assistants would use the instrument.  Presence is answered with a yes/no choice.  Frequency and intensity are rated on a 13-point horizontal Likert-type scale anchored with verbal descriptors (i.e., never, occasionally, moderately often, often, and always) for frequency; mild, moderate, and severe for intensity. | - | USA |
| [21] | **RaPID** | Observational rating scale. | The RaPID consists of four dimensions that include eighteen (clustered) items. | The four dimensions and the include clustered items are as follows:   1. behavioural (N = 11) 2. emotional (N = 2) 3. autonomic (N = 2) 4. postural (N = 3). | The review included no information on who would use the PPI.  The items are scored on a four-point scale (0 absent to 3 severe), where the total score of the scale ranges from 0 to 54.  Examples of items include tense body language, tearfulness, sweating, and general increase in muscle tone.  Observers score each item based on complaints, symptoms and signs occurring during one week, prior to using clinical judgements from a range of information such as clinical notes and interviews with staff, patient, and carer. | - | USA |
| [41] | **REPOS** | Observational rating scale. | REPOS consists of a list of ten behaviours. | The ten behaviours are related to:   - facial expression - emotional status - motor behaviour - vocalisation. | The review indicated that the REPOS is designed for use by nurses.  The ten behaviours are scored as absent (=0) or present (=1) after a two-minute observation. | A REPOS score of ≥ 3 indicates pain, and requires intervention on the guidance of a step-by-step decision tree. Part of the decision tree requires the nurse to rate the pain intensity experienced. Pain is present and action is needed if REPOS score is ≥ 3 and NRS score ≥ 4.  Method of administration, scoring instruction and interpretation of tool score are clear. | Netherlands |
