# Supplementary material for: Pain assessment for people with dementia: a systematic review of systematic reviews of pain assessment tools
Source: BMC Geriatr. 2014 Dec 17;14:138. doi: 10.1186/1471-2318-14-138 (PMC4289543; doi:10.1186/1471-2318-14-138)
Supplement: Supplementary file 8 — Additional file 8: Summary of the tools - settings of use. Summary of the data extracted from the reviews pertaining to the settings were the tools had been studied, tested or used for clinical practice. (DOCX 21 KB) [file 12877_2014_1072_MOESM8_ESM.docx]

**Table AF8. Summary of the tools: settings of use**

Summary of the data extracted from the reviews pertaining to the settings were the tools had been studied, tested or used for clinical practice (table cells left empty when no data were available).

| **Review ID** | **Name of Tool** | **Setting** |
| --- | --- | --- |
| [21] [22] [27] [37] [41] [42] [44] | **Abbey Pain Scale** | Six of the seven reviews provided information on the setting where the scale was developed or has been evaluated. Five of the reviews stated that it was tested in aged care and/or residential care facilities. One review stated it was evaluated in long term care facilities. Two reviews stated that it was evaluated with patients who had end or late stage dementia. One review mentioned that evaluations tool place in Australia. |
| [27] [37] [43] [42] [44] | **ADD Protocol** | Three of the reviews discussed the setting in which the tool was tested. All three stated it was tested in long-term care facilities, one mentioned it was tested on people with dementia, one gave details as follows: used by 32 volunteer nurses from 25 long term care facilities. Applied when patient displayed signs of discomfort - nurse asked to record certain behavioural indicators. |
| [42] [44] | **Behavior checklist** | Both reviews provided an overview of the setting: hospitalized older patients. One review also stated it was used with cognitively impaired patients showing pain. |
| [21] [22] [27] [37] [43] [41] [42] [44] | **CNPI** | No data for two reviews. The tool was tested in a convenience sample of hospitalised patients aged 65 and older with a hip fracture. Also studied in nursing homes in USA and Norway (Norwegian sample diagnosed with dementia). Cognitively impaired and intact hospital patients with postoperative pain. |
| [43] | **Comfort Checklist** | - |
| [41] | **CPAT** | Long term care facilities in the USA |
| [21] [22] [27] [37] [41] [44] | **Doloplus-2** | Two reviews provided no data.  Three reviews stated it was tested in palliative care hospitals and geriatric clinics (and unclear what other setting) in France and Switzerland. One review stated it was studied with nursing home patients with dementia (Netherlands), residents with moderate to severe dementia in dementia care units in long term care facilities (Taiwan), or patients with severe cognitive impairment resident in nursing homes (Norway). |
| [22] [27] [37] [43] [42] [44] | **DS-DAT** | One review provided no data on the setting, but specified the target population as patients with Alzheimer Disease. One review mentioned nursing homes. One review reported it was tested in a range of settings - long term care facilities and in hospitals -, including the acute care setting at two medical units of a large, tertiary care hospital and three other reviews stated this included Veterans Administration facilities. |
| [21] | **ECPA** | The review indicated that the tool was tested with hospitalised patients in a long-term stay department. |
| [21] | **ECS** | - |
| [22] | **EPCA-2** | - |
| [44] | **FACS** | Used in several studies of cognitively impaired older adults |
| [37] | **FLACC** | Long-term care |
| [41] | **Mahoney Pain Scale** | Nursing homes |
| [22] [41] [42] | **MOBID** | Two reviews indicated that the tool was tested with nursing home patients. One review stated that both MOBID-1 and MOBID-2 were tested with severely cognitively impaired residents of nursing homes in Norway. The third review provided no information. |
| [21] [22] [27] [37] [41] [42] [44] | **NOPPAIN** | There was variation of where the NOPAIN was evaluated in practice. One review each identified nursing homes, care homes and assisted living facilities (these may be the same type of institution, but use of different terminology is confusing). One review identified that there was a mixture of cognitively intact and individuals with mild to moderate cognitive impairment. One review identified a sample of nursing assistants who completed the pain assessment, while another review identified that the pain assessment tool was used by both trained and untrained staff. Two reviews identified the use of video recordings of an actress portraying a bed-bound patient with severe dementia during caregiving. One review contained no data about the setting and one review was unclear about the setting. |
| [21] [43] | **Observational Pain Behaviour Tool** | One review suggested the tool was designed for (and studied in) a hospital setting; the other provides no data on this. |
| [21] [22] [27] [37] [41] [42] [44] | **PACSLAC** | Two reviews provided no data. From the other six reviews, the setting remains unclear (both for setting the tool was designed for and studied in, if different). One review suggested the tool was tested with registered nurses (unclear which setting); another that nurses working in long term care facilities and nursing home patients with dementia were involved in the study; from one review it could be inferred that the setting was the nursing home. |
| [21] [22] [27] [37] [43] [42] [44] | **PADE** | No data from one review. From the other six reviews, it appeared that the tool was studied (and perhaps designed for) long-term care residential settings. However there is some confusion on the nature of this setting, and whether it involved more than one. The setting was described as: long-term care facilities; residential and skilled care facilities; three clinical settings - all appeared to be long term care settings with residents suffering from dementia; and the people residing in this setting as: older adults or elderly people; residents with advanced levels of dementia; residents suffering from dementia; or elderly people with mostly severe dementia. |
| [21] | **Pain assessment scale for use with cognitively impaired adults** | A dementia care unit and a psycho-geriatric unit |
| [21] [22] [27] [37] [43] [41] [42] [44] | **PAINAD** | The setting was variously described a long-term-care dementia special care units; dementia special care units in hospital; patients with moderate cognitive impairment in nursing homes; and a residential dementia care ward. Three reviews provided no data. |
| [22] | **PAINE** | - |
| [42] [44] | **PATCOA** | Both reviews reported on a hospital setting. |
| [44] | **PBM** | Rehabilitation facility |
| [22] | **PPI** | - |
| [43] | **PPQ** | - |
| [21] | **RaPID** | A hospital setting (psychiatric and medical care units) |
| [41] | **REPOS** | Nursing homes |
