# Supplementary material for: Pain assessment for people with dementia: a systematic review of systematic reviews of pain assessment tools
Source: BMC Geriatr. 2014 Dec 17;14:138. doi: 10.1186/1471-2318-14-138 (PMC4289543; doi:10.1186/1471-2318-14-138)
Supplement: Supplementary file 9 — Additional file 9: Summary of the tools - reliability and validity. Summary of the data extracted from the reviews regarding the reliability and validity of each tool. (DOCX 48 KB) [file 12877_2014_1072_MOESM9_ESM.docx]

**Table AF9. Summary of the tools: reliability and validity**

Summary of the data extracted from the reviews regarding the reliability and validity of each tool (table cells left empty when no data were available)

| **Review ID** | **Name of Tool** | **Reliability** | **Validity** |
| --- | --- | --- | --- |
| [21] [22] [27] [37] [41] [42] [44] | **Abbey Pain Scale** | All seven reviews mentioned the reliability of the scale. One review did not provide data on reliability coefficients.  A summary of the data provided across the reviews as follows:  Internal consistency (measured using Cronbach’s alpha): 0.74 – 0.81 (on a sample of 61 patients). One review summarised data from a Japanese study as follows: n=171 alpha =0.645, MMSE=0 n=58 alpha=0.719. Overall estimates of internal consistency therefore range from 0.645-0.81  Inter rater reliability (measured using intraclass correlation coefficients): estimates given in the reviews as follows: 0.44-0.63 (n=18 patients); 0.845 (n=45). Overall estimates of inter rater reliability therefore range from 0.44-0.845.  One review provided data for test-retest reliability (measured using intraclass correlation): 0.657 (n=57) | All seven reviews mentioned validity, however one review only outlined confounding factors. All of the other reviews summarised the concurrent validity of the scale, which was evaluated against the holistic impression of pain as assessed by the nurse: gamma 0.586, p < 0.001. One review also stated that validity testing based on nurse judgment of pain intensity is not substantiated in the literature, particularly, as in this case, without evidence supporting the expertise of the raters.  Two reviews also provided data for the scales predictive validity, which was assessed by a change in mean pain score from pre-intervention (M=9.02; two values for the SD 0.48 and 3.75) to post-intervention (M=4.21; two values for SD 0.41 or 3.20), p<0.001. One review also suggested that it is unclear what unit of analysis was used for examining pre and post test score changes (pain, episode, resident, mean score). |
| [27] [37] [43] [42] [44] | **ADD Protocol** | All reviews mentioned reliability. One review stated it has demonstrated inter-rater reliability. The other four reviews provided an assessment of reliability (the same data) as follows: calculated as percentage of agreement between two nurses assessing four residents, was 86% for the total tool. Inter-rater reliability was 100% for medication use, 76% for nonpharmacological interventions and 87% for behavioral assessment of symptoms.  Two reviews indicated that it has not been tested for test-retest reliability or internal consistency. One review suggested that internal consistency evaluation may not be appropriate. | Four of the five reviews mentioned tool validity. One review stated it demonstrates predictive validity. Two of the reviews provided the same data on predictive validity as follows: Pre-intervention sample had an average of 32.9 (SD 16.8) behavioral symptoms associated with discomfort compared to 23.5 (SD 16.5) for post-intervention, a significant decrease in discomfort (t = 6.56, p = 0.000). This data is reported slightly differently by one review, which reported a change of behavioural symptoms of 84%. One review also reported data on use of interventions as follows: use of the ADD protocol was associated with a significant increase in the use of pharmacologic (t = 2.56, p = 0.012) and nonpharmacologic comfort interventions (t = 3.37, p = 0.001). |
| [42] [44] | **Behavior checklist** |  | 1 review suggested that there were lower scores after pain medication (n=13) but no other data provided. |
| [21] [22] [27] [37] [43] [41] [42] [44] | **CNPI** | One review did not report reliability data.  **Inter-rater reliability**  Six of the reviews reported percent agreement 93%. Five reviews also reported kappa statistics of: 0.625 to 0.819 for behaviours observed (on 12 subjects)  **Internal consistency**  Seven reviews reported internal consistency data. All studies reported the results as a Kuder Richardson 20 (KR 20), six of the studies gave values as: at rest, alpha coefficient 0.54 (confidence interval [CI] =0.36 to 0.68 and 0.64 on movement (95% CI = .49 to .75). However, one study reported alphas of 0.54 (95% confidence interval, CI 0.38-0.68) at rest and 0.54 (95% CI 0.38-0.68) with movement  One review also reported the following data: Nygard: test retest n=46 k=0.23-0.66, interrater n=46 k=0.45-0.69; Ersek internal consistency n=60 at rest; a=0.92-0.97 movement: a=0.74-0.90; interrater n=60 at rest k=0.43 movement k=0.25 | Seven reviews reported data on concurrent validity – where self reports of pain using the Verbal Descriptor Scale were compared to CNPI scores. Evaluations differed as follows:  Spearman r=0.30-0.50 (n=32)  Spearman correlation coefficients at rest were 0.37 (p = 0.001) and with movement 0.43 (P < 0.001) (n=64). Total population (cognitively intact and cognitively impaired groups).  Cognitively impaired group (n = 32), coefficients at rest were 0.30 (p = 0.076) and with movement 0.46 (p = 0.009).  Cognitively intact group (n = 32), coefficients at rest were 0.50 (p = 0.003) and with movement 0.39 (p = 0.032).  Concurrent validity suggested as higher CNPI scores during movement than at rest. |
| [43] | **Comfort Checklist** | - | - |
| [41] | **CPAT** | Inter rater reliability n=80 ICC=0.71  Test retest n=88 ICC 0.67. | Concurrent (corr. with self report/other pain tool) DS-DAT (r=22, p=0.076; r_s_=0.25 p=0.048).  Discriminant validity differences in scores before and after a known painful event M=0.33 p=0.043 |
| [21] [22] [27] [37] [41] [44] | **Doloplus-2** | Inter rater reliability: 5 reviews mentioned inter rater reliability with considerable variation in the data presented. One review summarised data in terms of mean scores for two samples (43 patients, 28 males and 15 females mean age 73.5, SD 7.2 and 41 patients, 9 males and 32 females) mean age 82 (SD 8.3). Total mean score for Rater A was 11.4/30 (SD 5) and for Scorer B 10.9/30 (SD 4.8). In the second site, the sample included Scorer A had a total mean score of 17.3/30 (SD 4.0) and Scorer B 17.1/30 (SD 4.6). Two reviews summarised the same data suggesting that interater correlation between physicians was not significant (p<0.0001). One review summarised it in terms of ICC, with values from 0.77-0.90 for the total scale and 0.60-0.96 for the subscales.  One review summarised test-retest reliability (83 patients): at Time 1 the mean score was 9.33/30 (SD 5.17) and at Time 2 it was 9.36/30 (SD 5.47). A student’s t-test was not statistically significant.  Internal consistency: five reviews provided data with a range from 0.668-through to 0.82 for the total scale. One review provided data for the subscales with estimates as follows:  Somatic a=0.60-0.79; psychomotor a=0.77-0.87; psychosocial a=0.58-0.78 | Six reviews mentioned validity. Of these 3 reviews provide data.  Convergent/construct validity:  Correlation between VAS and Doloplus-2: Spearman rho=0.46 (1 review); r=0.65 (n=143) (1 review), significant but not data (2 reviews). Between the VAS/VRS/FPS (n=141) r = 0.31-0.40 (1 review).  One review gives data for comparisons with PAINAD r=0.34 PACSLAC r=0.29 and functional ability r=0.38 p<0.05. |
| [22] [27] [37] [43] [42] [44] | **DS-DAT** | One review gave no detail on reliability data.  Three reviews reported the findings from one study where interrater reliability was evaluated at four time points for five to nine subjects noting Pearson correlation coefficients ranging from 0.86 to 0.98. Another 3 reviews summarise the findings from additional studies giving Pearson correlation coefficients ranging from 0.61-0.98 with an ICC of 0.74.  One review reports percentage agreement results of 84% after 30 hours training and data collection (12 subjects) and 94% with an additional 5 hours training (32 subjects).  Four reviews reported test-retest reliability data: Pearson correlation coefficients of 0.60 (p < 0.001) and non significant paired t-test (p = 0.46) indicating no change after 1 hour (n=68).  Six reviews report internal consistency data giving values ranging from an alpha of 0.74 to 0.89. | Construct validity: correlations with Pittsburgh Agitation Scale (r =0.51, p < .01) (mentioned by 3 reviews)  The DS-DAT was able to detect significant differences in discomfort in a sample of 20 subjects identified by staff as having a fever episode (F1,19 = 167.02, P < 0.001): the mean score at baseline was 7.7 (SD 1.2), at peak 11.9 (SD 1.0), and on resolution 8.1 (SD 1.2) (2 reviews)  Significant correlations between the DS-DAT and the aggressive subscale of the Cohen-Mansfield Assessment Inventory (r = 0.25) and Verbal Descriptor Scale (r = 0.35), respectively (1 review)  Significant correlation (r =0.56 [pain by VAS] and r =0.81 [discomfort by VAS]) between DS-DAT and subscales of self-report VAS) (n=19). (2 reviews)  Concurrent (corr. between two scores) n=19 PAINAD r=0.76 (2 reviews)  3 reviews also report scores on DS-DAT were higher in uncomfortable situations compared to comfortable situations.    One review highlights that external validity limited because scale only tested on males. |
| [21] | **ECPA** | Internal consistency: α = 0.70  Inter-rater reliability by Intra Class r = 0.80. Review added that no data is available on Intra-rater or Test-retest reliability. | Construct validity (relation to other pain tools): VAS-EPCA Pearson r = 0.67 (N = 16). |
| [21] | **ECS** | - | - |
| [22] | **EPCA-2** | Internal consistency α=0.79; subscale at rest = 0.73; subscale during caregiving 0.75.  Inter-rater reliability by Intraclass Correlation as being ICC=0.877 (n=340); doctor-doctor ICC=0.869 (n=56); nurse-nurse ICC=0.897 (n=63); doctor-nurse ICC=0.852 (n=55). | Reported tool has good face validity. The review reported on the convergent validity; Calculated by the correlation between EPCA-2 and Clinical Pain Scores r=0.846 (n=340), and EPCA-2 and analgesic prescription of opioids r=0.782 (n=112) and non-opioids r=0.730 (n=171); responsiveness was assessed by changes of EPCA-2 and Clinical Pain Scores after treatment r=0.663 (n=283) |
| [44] | **FACS** | Inter rater: 43-93% agreement (frequency); Pearson’s r =0.82-0.97 (intensity).  Intrarater: 79-93% agreement (frequency); Pearson’s r = 0.88-0.97 (intensity) | Concurrent (corr. between 2 pain scales): n=58. PBM: r=0.02 (guarding), 0.13 (bracing), 0.41 (grimacing);  Concurrent (corr. between pain scale and proxy pain report) n=26 AU (frequency), r=0.62; AU (intensity) r=0.73;  Convergent (corr. between pain scale and self report) n=55 CAS r=0.05, n=82 CAS/VDS, n=12 CAS;  Discriminant n=26 - higher frequency and intensity of AUs during painful event, n=82 significant higher scores during knee-bending >standing >reclining |
| [37] | **FLACC** | Internal consistency reliability data are not available.  Interrater reliability was evaluated based on 69 valid FLACC observations rated by three trained research observers independently recording pain assessments. Kappa statistic was 0.404 or less. These researchers concluded that the FLACC is not a useful pain assessment tool for cognitively impaired elderly; however, small sample limits conclusions. No data are available regarding test-retest reliability. | Based on 69 valid FLACC observations and 56 observations on the Modified University of Alabama (UAB Pain Behavior Scale), the FLACC and UAB Pain Behavior Scale were significantly correlated. However, Spearman’s rho data are not reported. Moreover, the UAB has not been validated in older adults with dementia and is questionable as an appropriate criterion measure for establishing construct validity in this population |
| [41] | **Mahoney Pain Scale** | Internal consistency - total scale a=0.76, behavioral observation subscale a=0.75, indicators for pain vs agitation subscale a=0.68. Inter rater reliability n=75 pleasant activity k=0.43-0.77, aversive activity k=0.55-0.85 | Criterion validity (corr. with proxy score) n=60 presence of pain k=0.86 |
| [22] [41] [42] | **MOBID** | Internal consistency in two reviews was noted as high (α = 0.90 to 0.91) for MOBID-1 (after exclusion of the two items: observation at rest and observation of teeth/mouth care).  The third review rated internal consistency as α=0.82-0.84.  Two reviews indicated the inter-rater reliability for inferred pain intensity as good (ICC = .70 to .96) with an overall score of ICC=0.76-0.82.  Interrater reliability for specific pain expressions were not comparable across the reviews. One review included subscale pain noises k=0.42-0.84, facial expression k=0.06-0.77, defence k=0.05-0.87 while a second review rated for pain behaviour k=0.41-0.90.  One review included test-retest reliability of pain intensity as ICC=0.60-0.94. The second review indicated the ICC between three raters as ICC=0.86.  One review provided data for MOBID-2: internal consistency a=0.82-0.84. interrater reliability for pain behaviour indicators k=0.44-0.90, test-retest k=0.41-0.83; for inferred pain intensity inter rater reliability ICC=0.90-0.94 test retest reliability ICC=0.60-0.92. Visual pain recordings inter rater reliability k=0.46-0.80 test retest reliablity k=0.48-0.93. Pain intensity inter rater reliabiilty ICC=0.80-0.84, test retest reliability ICC=0.61-0.94. | Two reviews indicated that construct validity had been evidenced. One review indicated correlation of MOBID-1 (r=0.82) and MOBID-2 (r=0.61) with the total score.  Two reviews provided data for concurrent validity between MOBID-2 and Numerical Rating Scale by doctors (r=0.41-0.64).  Two reviews indicate values for discriminant validity in that pain intensity scores were substantially higher after the MOBID procedure than after regular care activities. One review indicated that higher overall pain scores were registered during the MOBID-1 procedure (M=4.4, SD±1.8) than after regular care activities (M=3.0, SD±1.9). |
| [21] [22] [27] [37] [41] [42] [44] | **NOPPAIN** | Three reviews indicated an inter-rater agreement of k=0.87 and a fourth review mentioned inter-rater reliability without providing statistical data.  Two reviews stated pain level comparisons of 82-100% agreement.  One review indicated presence of pain indicators k=0.72-1.00, pain intensity ratings ICC=0.72-1.00, test-retest reliability presence of pain indicators k=0.70-0.85 and pain intensity ratings ICC=0.68-0.95 for study 2.  One review indicated a moderate reliability testing and another review included no data on reliability.  One review indicated that no report of internal consistency was currently available. The review went on to report that interrater reliability was evaluated in Study 2 (using videotapes of nursing assistants performing morning care tasks with residents with dementia). Twenty-six videos were shown to six untrained nursing assistants and to 6 nursing assistants who had received 1 hour of training on use of the NOPPAIN.  The same review reported that interrater reliabilities were moderate to strong for all tool items and that they improved with 1 hour of training. Test-retest reliability was evaluated in Study 2 with a subset of untrained nursing assistants. Results indicated low to moderate test-retest reliability at both 2 and 24 hours. Only the pain thermometer was stronger at 2 than 24 hours. | Three reviews indicated that validation was measured by using video uptakes of an actress portraying a patient with severe dementia receiving care from a nursing assistant. Two reviews stipulated that six standard videotaped patient scenarios portraying the continuum of pain intensity levels were used. One review explained that a geropsychologist and a palliative care physician developed the nonverbal scripts for each pain condition. The standardised videotaped patient scenarios were regarded as the gold standard.  One review specifically mentioned that the NOPPAIN pain intensity was measured using a Likert response scale.  One review indicated that nursing assistants watched and rated videos using the NOPPAIN assessment process and completed global pain rating for each video. The review went on to say that nursing assistant’s global pain rating on the NOPPAIN and pain levels portrayed in the videos resulted in a weighted kappa statistic of 0.87. A second review substantiated the overall agreement between raters and the video portrayal of pain as being K = 0.87.  One review identified convergent validity by comparing NOPPAIN scores with detailed-coded behavioural observation ratings. Ratings between the two measures had been scored by two raters who were undergraduate nursing students and were significantly correlated for each of the six pain indicators in the total sample (p<0.001).  One review indicated a discriminant validity rating as Bradley-Terry model: deviance GFI=18.14 (10).  Four reviews stated a pain level comparison of 82%-100% agreement. Two reviews indicated that the lowest intensity pain condition had the smallest parameter, with parameter size increasing with each subsequent level of the pain response scale. To assess construct validity in Study 2, sensitivity and specificity were evaluated comparing NOPPAIN ratings by untrained nursing assistants to physician NOPPAIN ratings and physician pain classification (pain/no pain).  One review only mentioned that the tool has moderate validity. |
| [21] [43] | **Observational Pain Behaviour Tool** | - | - |
| [21] [22] [27] [37] [41] [42] [44] | **PACSLAC** | Inter-rater reliability: 4 reviews provided data. Reliability reported as 94% agreement (2 reviews), ICC 0.77-0.96 (three reviews, one suggested that this is for the subscales). One review reported ICC of 0.93-0.96 for the total scale.  Intra-rater or test/retest reliability: ICC of 0.72-0.92 –(one review suggested this is for the subscales). ICC for total scale =0.86 (1 review)  Internal consistency: Five reviews reported data for the complete scale giving values as follows 0.82-0.86; 0.85, 0.82-0.87 (a=0.82); 0.74 -0.92.  Four reviews provided data for subscales giving ranges as follows: 0.72-0.82; 0.55 to 0.85; 0.55-0.73; 0.20-0.76. | Discriminant validity: Five reviews reported discriminant validity, evaluated through the ability to discriminate among painful, calm, and nonpain-related distress events (F3,117 = 108.1, p < 0.001) (2 reviews). Two reviews reported r=0.80 between two pain situations.  Criterion validity: compared through using nurses’ perceptions of patient’s pain. Pearson correlation coefficient for Pain Event 1 was 0.35 (p < 0.05) and for Pain Event 2 was 0.54 (p < 0.001) (1 review), proxy pain ratings from nurses and expert rater; r=0.72-0.80 (1 review); Global intensity ratings, r=0.39-0.54 (4 reviews). |
| [21] [22] [27] [37] [43] [42] [44] | **PADE** | No data from 1 review.  **Inter rater reliability:** Reported by 6 reviews. Overall ICC are given as; 0.93-0.95 (n=25 and 40); 0.54-0.96 (3 reviews); 0.81-0.96. Three studies also provided data for subscales; Part 1 (physical) 0.93-0.95 (2 reviews) 0.93 (1 review); Part II (physical) 0.54-0.89 (2 reviews) 0.81 (1 review); Part III (functional) 0.93-0.94 (2 reviews) 0.96 (1 review). (n=24 residents, 784 observations for 1 review that reported separate scale results)  **Test-retest reliability (temporal stability**): Reported by 6 reviews. Reported as an overall figure: ICC = 0.70-0.98 (1 review), by subscale as follows (3 studies): Part I (physical) ranging from 0.70 to 0.98, for Part II (global assessment) from 0.34 to 0.70, and for Part III (functional) from 0.89 to 0.98 and by study (1 review) ICC = 0.34-0.89 (study 1) and 0.70-0.98 (study 2).  **Internal consistency:** Reported by 5 reviews.  Overall internal consistency reported by one review α = 0.77-0.88; and α = 0.24 to 0.88 (3 reviews). Three reviews reported by subscale Part 1 (physical): 0.76-0.88; 0.77; and Part III (functional): 0.23-0.63; 0.63; 0.24-0.63 | No data from 1 review.  **Construct/Discriminant validity:** All six reviews with data, reported comparison to the Cohen-Mansfield Agitation Inventory (CMAI). One review gave no data, two provided ranges of r=0.30-0.40; three provide data of correlation between the CMAI verbal subscale and PADE part 1: r=0.296, p<0.001; one review provided data on correlation between PADE Part III and the three CMAI subscales; r=0.40, 0.40, and 0.42, and one review the correlation between PADE Part II and the three subscales (physically, verbally, and nonphysically agitated): r = 0.396, 0.398, and 0.421 respectively, all p < 0.01  **Criterion validity**  One review reported evaluation as follows: Significant painful conditions rated by chart review: 10 positive compared to 30 negative; no statistically significant differences in PADE or agitation scale scores. Significant painful conditions as judged by nurse: 8 positive compared to 32 negative; statistically significantly higher scores in the pain group on verbal agitation (z = −2/83, p < 0.011), Part I (z = −3.44, p < 0.01), Part II (z = −4.30, p < 0.01) and Part III (z = −2.31, p < 0.01) Psychoactive medication use: number of subjects in each group not reported; those on psychoactive medications had statistically significantly higher scores on physical agitation subscale (z = −3.34, p = 0.01) verbal agitation subscale (z = −1.95, p = 0.05) and Part III (z = −3.25, p < 0.01).  Another review reports the following: Criterion validity was conducted in the nursing home. Construct validity was measured in the nursing home by dividing patients, using a chart review, into two categories: with painful conditions and without painful conditions. No significant differences between the two groups were found on the PADE subscales, providing support for the validity of the measure. Significantly higher scores in group 'pain is a clinical factor'. No significant differences between with and without painful conditions. |
| [21] | **Pain assessment scale for use with cognitively impaired adults** | - | - |
| [21] [22] [27] [37] [43] [41] [42] [44] | **PAINAD** | Inter-rater reliability ranges given were: r=0.82-0.97 (five reviews) and 0.72-0.89 (one review). No data for two reviews, one of these stated that IRR was adequate.  Ranges on scores for Internal consistency given were: α=0.5-0.65; 0.5-0.67; 0.69-0.74 (two reviews); <0.7; 0.7-0.72; 0.85-0.86.  Breathing was the poorest item. | Criterion validity: Correlation coefficients with comparable scales: Pain VAS 0.75,0.75 p<0.001; DS-DAT 0.76, 0.56 p<0.16; and Discomfort VAS 0.76.  Before and after pain medication comparisons: One review stated a significant difference (detail missing), one stated significant fall in score p<0.001. High correlations between PAINAD and nurses reports (Kendell’s tau 0.84)  Comparison with another behavioural measure (PACSLAC) was mentioned but correlation not specified. One review described construct validity as adequate but did not provide data. |
| [22] | **PAINE** | Internal consistency alpha = 0.75-0.78;  IRR r=0.999 (n=41) and r = 0.711 (n=36);  test retest reliability r = 0.783 (n=39) | Correlation with PADE r=0.65, n=91 |
| [42] [44] | **PATCOA** | Both reviews reported inter-rater reliability ranging from 56.5% to 100% for each non-verbal indicator. Internal consistency was poor, α=0.44. Correlation with a pain VAS was low at r=0.3. No cut-off score was established. | Content validity was established based on factor analysis. Convergent validity was assessed by correlating scale score with VAS self-report (r=0.41). The sample was very specific, including only older adults after orthopaedic surgery. |
| [44] | **PBM** | Interrater: ICC = .10-.87 | Concurrent (corr. between pain scores) n=58 r=0.02-0.41;  Convergent (corr. between pain scale and self report) n=55, CAS: r=0.11(bracing), 0.21(guarding), 0.30 (grimacing);  Discriminant n=82, significant higher scores during knee-bending > standing > reclining |
| [22] | **PPI** | Scale reliability 0.95-0.97 (n=114);  Test-retest reliability (Pearson value r=0.436) | PPI significantly correlated with Memorial Pain Subscale (r=0.67), Verbal Scale (r=0.54), VAS (r=0.55), RAND Health Survey and Dartmouth COOP Chart (r=0.72). |
| [43] | **PPQ** | Test–retest reliability: All three items were significantly and highly correlated with each other at the two time points (presence of pain, r = 0.84, p = 0.0007; frequency of pain, r = 0.87, p = 0.0003; intensity of pain, r = 0.84, p = 0.0006) | Construct validity: PPQ scores were also significantly related to amount of pain medication used by residents during that time (presence of pain, r = 0.37, p = 0.0075; frequency of pain, r = 0.55, p = 0.0001; intensity of pain, r=0.41, p = 0.0022) |
| [21] | **RaPID** | Inter-rater reliability: mean 0.97 based on interviews with caregiver-patient. Intra-rater or Test-retest reliability: test-retest >0.75 for all items based on Interviews with caregiver-patient.  Internal consistency - the total scale (α = 0.79) Homogeneity IC : 0.79 total scale  Good inter-rater reliability (mean .97). Similar high scores were found for test-retest reliability (ranging from 0.84 to 0.98) | Construct validity - relation other pain tools: RaPID/McGill/VAS scores r = 0.8–0.86 To establish concurrent validity, RaPID scores were compared with the McGill Pain Questionnaire and a VAS. Findings showed that the instruments correlated highly with each other. |
| [41] | **REPOS** | Internal consistency of the REPOS was moderate (KR=0.49).  Inter-rater agreement (ICC=0.92) and intra-rater agreements (ICC=0.90-0.96). Case Group 124, Control Group 50 | Convergent validity; Correlations between REPOS and proxy nurse ratings using the VRS were low to moderate (r_s_=-0.12-0.39).  Criterion validity; correlation between REPOS and PAINAD; (r_s_=0.61-0.75). |
