# Supplementary material for: Pain assessment for people with dementia: a systematic review of systematic reviews of pain assessment tools
Source: BMC Geriatr. 2014 Dec 17;14:138. doi: 10.1186/1471-2318-14-138 (PMC4289543; doi:10.1186/1471-2318-14-138)
Supplement: Supplementary file 10 — Additional file 10: Summary of tools - Content-validity-User centredness. Analysis of the data extracted from the reviews regarding conceptual foundation of each tool and ways content validity was explored. These aspects also pertain to the user-centredness of the tools. (DOCX 26 KB) [file 12877_2014_1072_MOESM10_ESM.docx]

**TableAF10: Summary of the tools: dimensions related to content validity and user centredness**

Summary of the data extracted from the reviews regarding conceptual foundation of the tools and ways content validity was explored. These aspects also pertain to the user-centredness of the tool - the instrument must be relevant to the condition, setting, the patient and the clinical users (table cells left empty when no data were available).

| **Reviews ID** | **Tools** | **Purpose and Target**  **Patient Group** | **Conceptual Foundation**  **and Derivation** | **Content Validity**  **Validation** |
| --- | --- | --- | --- | --- |
| [21] [22] [27] [37] [41] [42] [44] | **Abbey Pain Scale** | Measure intensity of pain in people with end-stage dementia | No conceptual presentation of conceptual basis. Based on existing studies. | Face and content validity established via focus groups / Delphi study with nursing and medical gerontology and pain experts. |
| [27] [37] [43] [42] [44] | **ADD Protocol** | Assess and treat discomfort and pain in people with moderate to severe dementia | Based on review of the literature and adaptation on the DS-DAT scale identifying 6 behaviour categories to assess pain and discomfort (facial expression, mood, body language, voice, behaviour and other).  Drawn up both as an assessment and treatment management approach. | - |
| [42] [44] | **Behaviour Checklist** | Assess pain in cognitively impaired older adults | - | - |
| [21] [22] [27] [37] [43] [41] [42] [44] | **CNPI** | Assess pain in older adults with cognitive impairment in acute and long-term care settings | Scale is a modification of the University of Alabama Birmingham Pain Behaviour Scale (UAB PBS) [designed to measure chronic pain].  Review of literature on pain behaviour in demented elders led to some items being eliminated and other redefined. | No formal content validation (e.g. by expert panel). Pilot tested with cognitively-impaired hip-fracture patients. |
| [43] | **Comfort Checklist** | Establish a set of documentation of behavioural symptoms that signal distress and possible pain, for people with Alzheimer’s disease | - | - |
| [41] | **CPAT** | Assess pain in nursing home residents with dementia.  Developed for use by certified nursing assistant direct-care providers | Items drawn from literature review and input of certified nursing assistants. | No formal content validation by an expert panel. |
| [21] [22] [27] [37] [41] [44] | **Doloplus-2** | Assess pain in non-verbal or cognitively impaired older adults.  Originally designed for use with young children. | Based on observation of three types of behaviour (somatic, psychomotor, psycho-social) in 10 different situations that could reveal pain (30). Coherence with research literature on pain in older adults with dementia.  One item supported by pain dementia literature in older adults. | - |
| [22] [27] [37] [43] [42] [44] | **DS-DAT** | Measure discomfort in older adults with advanced dementia who have lost cognitive capacity and verbal communication ability.  Developed for research. | Generated from semi-structured interviews with staff in Alzheimer centres -> list of 26 behaviours to rate.  Pilot tested and reviewed -> cut to 18 behaviours. Second pilot study re-tested the items -> a 9 item scale. | - |
| [21] | **ECPA** | Originally designed for use with young children | Indicates only that the scale was adapted for use with the elderly. | - |
| [21] | **ECS** | Detect changes in behaviour in older patients with and without communication difficulties | Designed by a multidisciplinary team of nurses and medical staff. Six of the ten items are assessed prior to care, two after care and two every 24 hours. | - |
| [22] | **EPCA-2** | Assess pain intensity in older persons | Based on 5 minutes observation before and during care-giving. Covers five categories recommended by the AGS Panel Evaluation. | - |
| [44] | **FACS** | Assess a range of emotions, including pain | Coders rate frequency and intensity of facial actions (such as raising brow, muscles, mouth stretch). Not designed specifically for older people. | - |
| [37] | **FLACC** | Assess post-operative pain intensity in young children | Conceptual soundness for use with older adults with dementia. Some items (e.g. leg kicking, squirming, quivering chin) have not been found to be related to pain in studies of older adults with dementia; moreover, scale does not cover all the AGS behavioural category guidelines. | - |
| [41] | **Mahoney Pain Scale** | Assess the presence of pain and estimate its severity in people with advanced dementia | Based on extensive literature review and survey with a large sample of aged care nurses caring for persons in nursing homes. | No formal content validity validation with an expert group. |
| [22] [41] [42] | **MOBID** | Assess pain in patients with severe cognitive impairment | One review indicated, it was based on extensive literature review by multi-disciplinary panel of experts in psychometrics, pain assessment and its management in cognitively impaired elderly.  Another review suggested developed in consultation with a panel of experts on pain in nursing home patients. | Face and content validity by a focus group of persons experienced in the evaluation and management of pain in nursing home patients. |
| [21] [22] [27] [37] [41] [42] [44] | **NOPPAIN** | Assess pain behaviours in patients with dementia.  Designed for use by a nursing assistant. | Multi-disciplinary panel of clinical and research pain experts in pain and psychometrics, focusing on observation of specific pain behaviours while doing common care tasks, while the patient is at rest and with movement. | Same group established its face and content validity. |
| [21] [43] | **Observational Pain Behaviour Tool** | Assess pain in elderly patients in hospital settings. | Developed by designers, using an “amalgam of pain behaviours”. These were adapted from an observation too of Keefe and Block for assessing chronic low back pain. No detail given on process of adaptation. | - |
| [21] [22] [27] [37] [41] [42] [44] | **PACSLAC** | Assess common and subtle pain behaviour for older people with severe dementia | Items derived in a three-phased study: interviews with 28 primary professional long-term caregivers of older adults with severe communicative limitations due to dementia; use of the checklist while carrying out potentially pain producing procedures; and evaluation of the tool in determining pain events.  Participating caregivers reported from their memory of patients they had cared for. | Validation by 40 experienced nurses (and care assistants). |
| [21] [22] [27] [37] [43] [42] [44] | **PADE** | Assess pain in patients with advanced dementia.  Designed to help caregivers assess patient behaviour that may indicate pain. | Based on a literature review, interviews with nursing staff in long-term care facilities, including care homes and patient observations in a dementia unit.  Based on assumption that caregivers can reliably rate pain intensity; this is not supported by the literature. | Content validity established with expert professional nurses with relevant experience in dementia and pain. |
| [21] | **Pain Assessment Scale for Use with Cognitively Impaired Adults** | Assess pain in cognitively impaired adults. | Developed from a literature review, advice from experts and focus group discussion with clinicians. | - |
| [21] [22] [27] [37] [43] [41] [42] [44] | **PAINAD** | Measure pain in patients with severe dementia. | Based on a literature review and consultation with experts (professional nurses / clinicians). Items were adapted ones from DS-DAT and FLACC (designed for measuring post-operative pain intensity in young children) | - |
| [22] | **PAINE** | Assess pain amongst older people with difficulties in communication | - | - |
| [42] [44] | **PATCOA** | Assess post-operative pain in acutely confused older adults | Based on nine cues in four categories (vocalisation, behaviour, motor activity, facial expressions) – no other information provided. | Tested as part of wider tool validation with older patients who were ‘cognitively intact’ |
| [44] | **PBM** | Measure pain in persons with chronic low back pain | Assesses pain behaviours based on videotape (10 minutes length) during walking, shifting, sitting, standing and reclining. | - |
| [22] | **PPI** | Measure self-reported pain, current compared to a week before | Self-assessment suggests problematic in use with persons with dementia. | -- |
| [43] | **PPQ** | Assess presence, frequency and intensity of pain | Relies on report of care-giver who knows the patient well. Designed for use by certified nursing assistants. | - |
| [21] | **RaPID** | Assess pain in elderly people with dementia | Developed from expert advice and literature review. No specific information is provided about the origin of the items in the instrument. | - |
| [41] | **REPOS** | Assess pain in nursing home residents incapable of self-report | Items developed via an expert panel to identify key pain behaviours based on video-taped observation of residents, leading on to a decision tool to enable nurses to determine relevant pain management interventions. | - |
