# Supplementary material for: Pain assessment for people with dementia: a systematic review of systematic reviews of pain assessment tools
Source: BMC Geriatr. 2014 Dec 17;14:138. doi: 10.1186/1471-2318-14-138 (PMC4289543; doi:10.1186/1471-2318-14-138)
Supplement: Supplementary file 11 — Additional file 11: Tools concurrent and criterion validity comparison table. Summary of the data on the concurrent and criterion validity of the tools, extracted from the reviews. (DOCX 27 KB) [file 12877_2014_1072_MOESM11_ESM.docx]

**Table AF11. Tools concurrent and criterion validity comparison table**

A summary of the data on the concurrent and criterion validity of the tools, extracted from the reviews (table cells left empty when no data were available).

|  | **Comparison with** | | | | | | | | | | | | | |
| --- | --- | --- | --- | --- | --- | --- | --- | --- | --- | --- | --- | --- | --- | --- |
| Name of tool | **CMAI** | **DS-DAT** | **Pittsburgh Agitation Scale** | **Cohen-Mansfield Assessment Inventory** | **PAINAD** | **PACSLAC** | **PADE** | **PBM** | **Memorial pain Subscale** | **Verbal scale** | **RAND Health Survey and Dartmouth COOP chart** | **McGill pain scale** | **Proxy pain reports (doctor or nurse)** | **Self-Report**  **(VAS)** |
| Abbey Pain Scale | - | - | - | - | - | - | - | - | - | - | - | - | 0.586 | - |
| ADD Protocol | - | - | - | - | - | - | - | - | - | - | - | - | - | - |
| Behavior checklist | - | - | - | - | - | - | - | - | - | - | - | - | - | - |
| CNPI | - | - | - | - | - | - | - | - | - | - | - | - | - | 0.30-0.50 |
| Comfort Checklist | - | - | - | - | - | - | - | - | - | - | - | - | - | - |
| CPAT | - | r_s_=22, p=0.076  r_s_=0.25, p=0.048 | - | - | - | - | - | - | - | - | - | - | - | - |
| Doloplus-2 | - | - | - | - | 0.34 | 0.29-0.38 | - | - | - | - | - | - | - | 0.31-0.65 |
| DS-DAT | - | - | 0.51 | 0.25 | - | - | - | - | - | - | - | - | - | 0.56-0.81 |
| ECPA | - | - | - | - | - | - | - | - | - | - | - | - | - | 0.67 |
| ECS | - | - | - | - | - | - | - | - | - | - | - | - | - | - |
| EPCA-2 | - | - | - | - | - | - | - | - | - | - | - | - | - | 0.846 |
| FACS | - | - | - | - | - | - | - | 0.02-0.41 | - | - | - | - | - | - |
| FLACC | - | - | - | - | - | - | - | - | - | - | - | - | - | - |
| Mahony Pain Scale | - | - | - | - | - | - | - | - | - | - | - | - | k=0.86 | - |
| MOBID | - | - | - | - | - | - | - | - | - | - | - | - | 0.41-0.64 | - |
| NOPPAIN | - | - | - | - | - | - | - | - | - | - | - | - | - | - |
| Observational Pain Behaviour Tool | - | - | - | - | - | - | - | - | - | - | - | - | - | - |
| PACSLAC | - | - | - | - | - | - | - | - | - | - | - | - | 0.35-0.54 | - |
| PADE | 0.30 – 0.42 | - | - | - | - | - | - | - | - | - | - | - | - | - |
| Pain assessment scale for use with cognitively impaired adults | - | - | - | - | - | - | - | - | - | - | - | - | - | - |
| PAINAD | - | 0.56-0.76 | - | - | - | - | - | - | - | - | - | - | 0.84 | 0.75 pain VAS and 0.76 discomfort VAS |
| PAINE | - | - | - | - | - | - | r=0.65 | - | - | - | - | - | - | - |
| PATCOA | - | - | - | - | - | - | - | - | - | - | - | - | - | 0.41 |
| PBM | - | - | - | - | - | - | - | - | - | - | - | - | 0.62-0.73 | r=0.11-0.30 |
| PPI | - | - | - | - | - | - | - | - | 0.67 | 0.54 | 0.72 | - | - | 0.55 |
| PPQ | - | - | - | - | - | - | - | - | - | - | - | - | - | - |
| RaPID | - | - | - | - | - | - | - | - | - | - | - | 0.8-0.86 | - | 0.8-0.86 |
| REPOS | - | - | - | - | 0.61-0.75 | - | - | - | - | - | - | - | -0.12-0.39 | - |
