# Supplementary material for: Pain assessment for people with dementia: a systematic review of systematic reviews of pain assessment tools
Source: BMC Geriatr. 2014 Dec 17;14:138. doi: 10.1186/1471-2318-14-138 (PMC4289543; doi:10.1186/1471-2318-14-138)
Supplement: Supplementary file 12 — Additional file 12: Summary of tools – feasibility. Summary of the data on the feasibility of each tool, extracted from the reviews, analysed in terms of ‘dimensions’ of feasibility – ease of use, time to complete, availability of instruction and guidelines, training required. (DOCX 35 KB) [file 12877_2014_1072_MOESM12_ESM.docx]

# **Table AF12. Summary of tools: feasibility**

# Summary of the data on the feasibility of the tools, extracted from the reviews, analysed in terms of ‘dimensions’ of feasibility – ease of use, time to complete, availability of instruction and guidelines, training required (table cells left empty when no data were available).

| **Review ID** | **Name of Tool** | **Overall ‘Ease of use’** | **Time to complete** | **Instructions on use** | **Guidelines on scoring** | **Training** | **Other evidence of/claims for feasibility** |
| --- | --- | --- | --- | --- | --- | --- | --- |
| [21] [22] [27] [37] [41] [42] [44] | **Abbey Pain Scale** | Three reviews indicated that this scale is easy to use. One review stated that it is a short and manageable scale. | Reviews agreed that it takes apparently 1 minute to complete but one review states that there is no published data to support this; another review suggests that information was derived from qualitative analysis of nurses’ feedback after testing. | Three reviews stated that clear instructions (specifically for scoring) and detailed item definitions are lacking. One review identified that few limited instructions are provided on the tool schema and some are presented on a poster. The same review points out that nurses are asked to use the tool when pain is suspected, but that it is unclear what triggers the assessment. | (see instructions on use) | One review mentioned that the level and length of training required is not reported. | - |
| [27] [37] [43] [42] [44] | **ADD Protocol** | Two reviews suggested that this tool appears complex, because it involves multiple steps and may require additional documentation to complete. | One review suggested that the use of the tool may require a considerable amount of time. | One review reported that method of administration is adequately described in articles about the tool. | - | Two reviews recognised the need for extensive training/education: one states that extensive training is required before use of the tool; the other that because the protocol involves complex clinical decisions, it also requires extensive education. | Two reviews concluded that the protocol appears to be comprehensive and conceptually sound but that it may be too complex for routine use and requires extensive training. One of the two reviews concludes that it may not be *clinically practical* for use in nursing homes.  One review reported that “evaluation of the protocol identified a number of problems with its use“ including time and lack of education [27]. |
| [42] [44] | **Behavior checklist** | One review suggested that the tool appears to be easy to use. | - | - | - | - | - |
| [21] [22] [27] [37] [43] [41] [42] [44] | **CNPI** | Six reviews suggested that the tool appears easy to use. Though one review mentioned that the tool’s ease of use has not been formally evaluated. | One review stated that the time needed to administer the tool has not been formally evaluated; one that it hasn’t been reported. | Three reviews stated that the method of administration is clearly described. | One review stated that the scoring instructions are available and clearly explained. | One review stated that the level of training and length of training time required to use scale are not reported. | - |
| [43] | **Comfort Checklist** | - | - | - | - | - | - |
| [41] | **CPAT** | Tool described as easy to use. | Tool described not too time consuming. Authors suggest takes about 1 minute to complete. | Method of administration reported as clear. | Scoring instructions reported as clear. | Training consisted of 45 minute training session. | Evaluation of feasibility of the tool by use of Practicality of Instruments Survey |
| [21] [22] [27] [37] [41] [44] | **Doloplus-2** | One review suggested that the tool appears easy to use; one review states that the tool has been reported ‘rather complex for staff within the care home settings’. | Time taken to administer the tool was mentioned in 3 reviews: one review stated that completion time was not reported; one review stated that it takes a few minutes to complete; one review that the authors state that the tool only takes a few minutes to complete, but without data reported to support this. | Two reviews stated that method of administration is clear; two reviews note that lexicon and instructions for use are available.  One of these reviews reported that a comprehensive supporting Web site is available, both in French and in English, but that translation issues are evident for use of Doloplus 2 in English. | Two reviews stated that scoring procedures/instructions are clear. However one review expressed concerns that certain items seem difficult to understand or interpret. | Two reviews noted that training requirements (e.g. level and length of training) are not reported. One of the reviews makes specific reference to the prospected user groups (health and social care providers as well as family of the older adult). | One review claimed that: “Validation provides evidence that repetition of assessments is *feasible* on one rating scale” [22] |
| [22] [27] [37] [43] [42] [44] | **DS-DAT** | One review expressed concerns over the complexity of the scoring schema, especially scoring of intensity and duration. | One review reported this to be a time-consuming scoring system. One review explained that the administration procedure requires waiting 15 minutes after a possible discomfort event, then observation of the individual at rest (min. 5 minutes); it concluded that the actual time needed to administer the tool may be considerably longer due to the complexity of the scoring. | - | Two reviews reported that the scoring instruction are comprehensive. | Three reviews reported that the training required is extensive – i.e. to be administered by well-trained raters. | Two reviews concluded that the tool’s feasibility is hindered by its complexity. One review noted how despite practical concerns over complexity and time to administer, DS-DAT “remains the gold standard by which nearly all additional observational pain assessment tools are evaluated by their developers” [43]. |
| [21] | **ECPA** | Tool described as ‘manageable’ | - | - | - | - | - |
| [21] | **ECS** | - | - | Lexicon and users' instructions reported available | - | - | - |
| [22] | **EPCA-2** | - | - | - | - | Tool described as requiring time for staff training and effective administration. | - |
| [44] | **FACS** | - | - | The review reported extensive information available on a Web site | - | - | The review suggested that the tool is *unsuitable for clinical practice* because of the complexity of learning the system and the use of video recordings |
| [37] | **FLACC** | - | - | - | - | - |  |
| [41] | **Mahoney Pain Scale** | Nurses reported finding it easy to use. | Time to administer the tool not reported. | Methods of administration reported as clear. | Scoring instructions and interpretation of tool score reported as clear. | The review reported that nurses had a 2h training session prior to use, but that it is unclear whether this would be required for routine use. | Evaluation of *clinical feasibility* by use of the Pain Scale Usage Questionnaire |
| [22] [41] [42] | **MOBID** | One review reported weaknesses with regard to the usability in clinical practice. | One review reported that the tool requires time commitment. However another review states that MOBID -2 appears to be time-efficient in use (mean 4.37 min, range 2-7 min). | One review stated the method of administration is clear; one that it is not. | Two reviews identified lack of clarity with regard to the scoring instructions. | One review reported that training involves a 2h session. | One review reported that studies [of MOBID-2] “indicate high to excellent aspects of reliability and validity of this assessment scale, including *feasibility* in clinical practice” [22].  However, one review suggested that the lack of scoring methods and the time required hinders the *clinical utility* of the tool [i.e. its feasibility]. |
| [21] [22] [27] [37] [41] [42] [44] | **NOPPAIN** | One review reported that according to the authors, the scale is easy to administer. | Three reviews indicated that the tool requires little time to complete.  There was a discrepancy with regard to the time – from 30 seconds to 15 minutes. This may be due to the inclusion or exclusion of the period of observation required prior to tool completion. | Three reviews indicated that the method of administration was described or clearly identified.  Two reviews indicated that the availability of illustrations make use in practice easier. | Two reviews indicated that scoring procedures are unclear.  Two reviews indicated that no criteria are provided for establishing low to high intensity of pain behaviour. One review stated that caregivers found it difficult to discriminate between mild and moderate pain. | One review reported the authors’ claim that the scale can be used with very little training. However, three reviews expressed concerns for the lack of clarity or data as to what investment in training is needed. | One review suggested that one of the strengths of the tool is *the ease of administration by nursing assistants.* |
| [21] [43] | **Observational Pain Behaviour Tool** | - | - | - | - | - | One review [31] suggests that the authors of the study “claim that the tool is practical” |
| [21] [22] [27] [37] [41] [42] [44] | **PACSLAC** | Three reviews seemed to agree that the tool, although being long, appears to be easy to use.  One reviews suggested that the tool appears complicated. | Three reviews indicated that the tool requires a limited amount of time i.e. about 5 minutes. However one review states that no data was provided on time taken to administer tool. | Three reviews stated that clear instructions for use were available on the tool form. | Two reviews stated that simple instructions on how  to score the tool are clearly described on the tool form | Two reviews mentioned training, one because level and length of training were not reported, and the other as it appears complicated at face value and necessitating training. | One review suggested that time to administer the tool would be “an important determinant in evaluating [its] *utility* [i.e. feasibility] [..] considering that it has 60 items” [41] |
| [21] [22] [27] [37] [43] [42] [44] | **PADE** | - | Five reviews noted that it takes 5 to 10 minutes to complete. However, one of the reviews pointed out that “data to support this [statement] are not provided” in the original study, and that “considering the complexity of the items, the variety of scaling approaches, and the expectation of finding answers in the patient record, this tool may take considerably longer to administer than the suggested 5-10 minutes” [19]. This observation was supported by two other reviews. | One review mentioned that an instruction manual has been developed but not available for the review. | Two reviews suggested the scale is long and has difficult format due to different scaling (Likert, VAS, Multiple choice), making scoring a challenge. | Two reviews suggested the tool is used by mostly or primarily nursing assistants. One the reviews suggested the use is after training and under nurse supervision.  One review suggested time required for training should be considered. | One review concluded that “time to complete is not known, and the complexity of some items, the variety in scoring, and the need to review chart documentation of the last 24 hours make the PADE *less feasible* in clinical practice” [44] |
| [21] | **Pain assessment scale for use with cognitively impaired adults** | Nurses reported the tool to be complex; difficult format due to different scaling and a long list. | Nurses reported the tool to be time consuming | - | - | - | - |
| [21] [22] [27] [37] [43] [41] [42] [44] | **PAINAD** | Two reviews reported the tool as user friendly/easy to use.  However, one review indicated that it seems easy to use, but the tool is complicated by an extensive list of explanations and definitions. | One review suggested that tool takes a few minutes to complete. One review explained that subjects are observed for 5 min before completing the tool, but that a clear recommendation for length of observation is not provided. | Two reviews reported that method of administration is clear and a guide with definitions of items is provided. | Two reviews indicated the scoring procedures are clearly described | Three reviews indicated that training is needed to use the scale – but this was variously described as from limited training to two hours. One review stated that level and length of training time required was not reported by the authors. | - |
| [22] | **PAINE** | - | - | - | - | - | - |
| [42] [44] | **PATCOA** | - | - | - | - | - | - |
| [44] | **PBM** | Described as short scale, easy to use. | - | Described as easy to use in terms of conciseness and clear item definitions . | - | - | The review suggested that the use of video recordings makes this scale *less feasible* in clinical practice. |
| [22] | **PPI** | - | - | - | - | - | - |
| [43] | **PPQ** | - | - | - | - | - | - |
| [21] | **RaPID** | Described as clustered but list of acceptable length |  |  | The review reported scoring interpretation not available. | - | - |
| [41] | **REPOS** | - | Two minute observation time required before completing the tool. | Method of administration clearly explained. | The review reported scoring instructions and interpretation of tool scores clearly explained. | The review reported level and length of training time not reported. | - |
