# Supplementary material for: Pain assessment for people with dementia: a systematic review of systematic reviews of pain assessment tools
Source: BMC Geriatr. 2014 Dec 17;14:138. doi: 10.1186/1471-2318-14-138 (PMC4289543; doi:10.1186/1471-2318-14-138)
Supplement: Supplementary file 13 — Additional file 13: Summary of tools - clinical utility. A summary of the data on the clinical utility of each tool, extracted from the reviews, analysed in terms of ‘dimensions’ of clinical utility (availability of cut-off scores and interpretation of scores for decision making) and overall evidence. (DOCX 31 KB) [file 12877_2014_1072_MOESM13_ESM.docx]

# Table AF13. Summary of tools: clinical utility

# A summary of the data on the clinical utility of the tools, extracted from the reviews, analysed in terms of dimensions of clinical utility (availability of cut-off scores and interpretation of scores for decision making) and overall evidence (table cells left empty when no data were available).

| **Review ID** | **Name of Tool** | **Availability of cut off scores** | **Availability of interpretation of scores for decision making** | **Evidence of *use* in clinical settings (for psychometric test and/or routine practice)** | **Evidence of clinical utility (or related summary statements)** |
| --- | --- | --- | --- | --- | --- |
| [21] [22] [27] [37] [41] [42] [44] | **Abbey Pain Scale** | - | One review stated that the interpretation of the tool score is clear. | It was reported that the tool was tested in residential care facilities. One review mentioned that it had been incorporated into the Australian pain guidelines. | One review noted the authors’ claim that the users reported that the scale was *useful* and brief. |
| [27] [37] [43] [42] [44] | **ADD Protocol** | - | - | One review mentioned that the protocol was introduced in 57 long term care facilities together with an education strategy for 12 months; the evaluation was done in a study with 32 nurses in 25 facilities. | One review pointed out that the protocol combines observation of pain behaviour and *a treatment intervention plan* for physical pain, affective discomfort, or both, to follow. One review reported that “evaluation of the protocol identified a number of problems with its use“ but that 44% of study participants commented that “they found it helpful” but suggested that the evaluation may have been clouded by the implementation/education strategy [27]. One review reported that  “In 88% of the cases, nurses reported the ADD  protocol as somewhat helpful to very helpful” [44]. |
| [42] [44] | **Behavior checklist** | - | - | - | One review reflected on the content of the tool, and how the tool may not be sufficient because lacks measures of frequency and intensity which are critical to determining the level of pain treatment necessary.  One review suggests that it provides important clinical information for assessing pain related behaviours in long-term care facilities |
| [21] [22] [27] [37] [43] [41] [42] [44] | **CNPI** | Two reviews reported that information on cut-off scores is not available | Two reviews stated that interpretation of score is unclear. One pointed out that there are no further instructions provided following assessment. | The tool was tested in acute care setting. | There appeared to be conflicting data: one review suggested this to be a clinically useful approach, though further evaluation of its usefulness being required; one review suggested the use of the scale may reduce the likelihood that pain treatment will be initiated and contribute to acute exacerbations of pain on movement. |
| [43] | **Comfort Checklist** | - | - | - | The review made a general statement that “in many care situations, descriptive methods *like the checklist* are *valuable* early in the assessment process when deciphering possible antecedents to behavioral symptoms is underway” [43]. |
| [41] | **CPAT** | - | The review state that interpretation of tool score is unclear and no instructions provided following assessment. | - | - |
| [21] [22] [27] [37] [41] [44] | **Doloplus-2** | One review reported that cut off scores are available but still to be validated | Two reviews reported that scoring interpretations are available. One of these noted that the tool “does produce a score that could be used to determine analgesia delivery, although the authors do not suggest how this aspect could be applied” [27]. | French version extensively tested in a variety of clinical settings | One review noted that “In acute settings, its *value might be limited* because patients must be well known to the nurses who have to complete the DOLOPLUS2, whereas the value of a scale becomes greater if it can be used without in-depth knowledge of the patient” [21].  One review suggested no practical utility to inform optimum timing of medication. |
| [22] [27] [37] [43] [42] [44] | **DS-DAT** | Two reviews reported that information on cut-off scores is not available | - | Tested in a variety of clinical settings | One review judged the scale to be of most use in research settings; for two reviews the usefulness of the tool depends on extensive/adequate training.  One review noted that treatment protocols for discomfort (measured with this tool) are different from those for pain which is not measured with this tool (limited utility for pain assessment could be inferred from this). |
| [21] | **ECPA** | - | Scoring interpretation not available | Tested in a sample of hospitalised patients in a long-term stay department | The tool’s clinical value needs to be examined further. |
| [21] | **ECS** | - | - | Not tested. Unclear whether it was used in practice. | - |
| [22] | **EPCA-2** | - | - | - | The tool’s clinical value needs to be examined further. The review judged the scale to be of most use in research settings. |
| [44] | **FACS** | - | - | - | - |
| [37] | **FLACC** | - | - | The review reported that the tool “is being used in some clinical settings with older adults” [19]. | Designed for use with children. Clinical usefulness of the tool in older adults remains unknown. |
| [41] | **Mahoney Pain Scale** | - | - | Tested in nursing homes | - |
| [22] [41] [42] | **MOBID** | - | - | Tested with nursing home patients. | One review suggested that “registering pain behavior indicators through active, guided movements is *helpful* in revealing pain intensity scores in older adults with severe cognitive impairments despite the varying degrees of reliability noted.” [42] |
| [21] [22] [27] [37] [41] [42] [44] | **NOPPAIN** | - | Three reviews reported that scoring interpretation is not available. One of these indicated that the there is no indication on how to proceed following assessment. | Unclear whether evaluation was carried out in a clinical setting. | One review suggested that: “The tool appears to be clinically useful given the ability of nursing assistants to use and the limited time required for completion.” [37] |
| [21] [43] | **Observational Pain Behaviour Tool** | - | One review reported that scoring interpretation is not available. | Pilot-tested in elderly hospitalised patients by observing pain behaviours, carrying out pain interventions and re-observing later to verify the effectiveness of the intervention. | One reviews pointed out that “the fact that carers without in-depth knowledge of the patient were able to use the tool is an important clinical advantage” [21]. |
| [21] [22] [27] [37] [41] [42] [44] | **PACSLAC** | One review reported that cut-off scores are not available. | Three reviews reported that scoring interpretation is not available. | Studied with nursing homes residents. | - |
| [21] [22] [27] [37] [43] [42] [44] | **PADE** | - | Two reviews reported scoring interpretation is not available | Tested in clinical setting. | Two reviews suggested clinical utility is hindered by the complexity of the scale and time-consuming nature. [i.e. by its feasibility]  One review noted “its clinical utility needs to be determined at the bedside” . |
| [21] | **Pain Assessment Scale for Use with Cognitively Impaired Adults** | - | - | Testing over a 3 months period. | The review reported that “The clinical utility of the scale has been pilot-tested in a small sample (N = 27 cognitively impaired elderly patients) by implementing the scale in practice over a three month period.” |
| [21] [22] [27] [37] [43] [41] [42] [44] | **PAINAD** | One review reported that cut-off scores are not available. | Conflicting data: One review reported that no guide to interpretation of the tool score is provided; one review that the interpretation of tool scores is clear. | Studied in clinical setting. Unclear whether long-term care dementia special care units or residential setting. | One review reported “positive findings in detection of changes in pain behavior following intervention in the quality improvement study” [37] – it could be inferred that clinical utility was formally evaluated. |
| [22] | **PAINE** | - | - | - | - |
| [42] [44] | **PATCOA** | The reviews reported that cut-off scores are not available. | - | Tested with cognitively intact patients with pain following orthopaedic surgery. | The reviews noted that the sample included cognitively intact older people - its clinical utility for people with dementia is unknown. |
| [44] | **PBM** | - | - | - | - |
| [22] | **PPI** | - | - | - | Self-reporting scale, often less applicable for individuals with severe dementia |
| [43] | **PPQ** | - | - | - | - |
| [21] | **RaPID** | - | - | Testing done in a hospital settings | - |
| [41] | **REPOS** | Cut-off scores available and validated | - | Studied in nursing homes | The review stated that “the REPOS is a potentially clinically useful tool in that it comprises a decision tree to enable nurses to determine relevant interventions required after pain assessment” |
