# Supplementary material for: Pain assessment for people with dementia: a systematic review of systematic reviews of pain assessment tools
Source: BMC Geriatr. 2014 Dec 17;14:138. doi: 10.1186/1471-2318-14-138 (PMC4289543; doi:10.1186/1471-2318-14-138)
Supplement: Supplementary file 14 — Additional file 14: Summary of reviews conclusions and recommendations. Summary of the overall conclusions and recommendations of each review and the tools considered in each of them. (DOCX 27 KB) [file 12877_2014_1072_MOESM14_ESM.docx]

**Table AF14. Summary of reviews conclusions and recommendations**

Summary of the overall conclusions and recommendations of each review and the tools considered in each of them

| **Review. ID** | **Tools included in the review** | **Conclusions and Recommendations** |
| --- | --- | --- |
| [22] | 12 tools: Abbey Pain Scale; ADD; CNPI; Doloplus-2; DS-DAT; EPCA-2; NOPPAIN; PACSLAC-D; PADE; PAINAD; PAINE;PPI | The majority of tools available require validation in people with dementia. Instruments appear sensitive to changes in pain intensity during treatment studies. Agreement between tools is limited. Some not validated in non-English speaking populations. Use of at least two different assessment approaches recommended. |
| [37] | 10 tools: Abbey Pain Scale; ADD protocol; CNPI; DS-DAT; Doloplus 2; FLACC; NOPPAIN; PACSLAC; PADE; PAINAD. | A number of tools demonstrate potential, but are in early stages of development and testing. With the exception of the DS-DAT and Doloplus 2, tools have limited testing beyond the initial study setting and sample. No testing done for use with [ethnic?] minority older adults with dementia. Strong evidence of reliability for only one tool, the DS-DAT, and none of the tools have demonstrated strong support for validity. This may reflect a need to revisit the tools’ conceptual foundation.  There is no standardized tool based on nonverbal behavioural pain indicators in English that may be recommended for broad adoption in clinical practice.  Authors emphasize that identification of pain indicators using a standardized tool is only one step in a complex diagnostic process. Use of a tool to identify pain behaviors should be integrated within a comprehensive approach to pain assessment in this population. One tool included in this review, the ADD protocol, is an example of such an approach. |
| [43] | 8 tools: ADD; CNPI; DS-DAT; Comfort checklist; Observed Pain Behavior Scale; PADE; PAINAD; PPQ | Each tool has merit and limitations. Need to further test tools reviewed. |
| [41] | 10 tools: Abbey Pain Scale, CNPI; CPAT; Doloplus-2; Mahoney Pain Scale; MOBID; NOPPAIN; PACSLAC; PAINAD; REPOS | No single tool identified to be recommended with confidence for use across both acute and long term care settings. Limitations include absence of cut-off scores indicating pain. Three of the tools; the MPS, the PACSLAC and the PAINAD show most promising outcomes and potential for use in acute and long term care settings. |
| [42] | 11 tools: Abbey Pain Scale; ADD; Behavior Checklist; CNPI; DS-DAT; MOBID; NOPPAIN; PACSLAC; PADE; PAINAD; PATCOA | Of the 11 scales reviewed, the PACSLAC appears to be the best pain assessment scale for nonverbal older adults with cognitive impairments or dementia. However more psychometric testing is recommended. Future research on pain scales for this population should identify which scales are reliable and valid at various levels of cognitive impairment.  Some of the reviewed scales do not provide either scoring methods or interpretations of scores. Therefore, it is recommended that scoring methods and their accurate interpretation be included in the assessment of these scales. In addition to behavioral and emotional pain indicators, future studies should focus on accurate pain assessment in culturally diverse older adults.  Social workers should be involved in a regular holistic multidisciplinary pain assessment (in nursing homes) and be trained in the use of the scales. |
| [27] | 9 tools: Abbey Pain Scale; ADD; CNPI; Doloplus-2; DS-DAT; NOPAIN; PADE; PAINAD; PACSLAC | Assessment tools such as a verbal rating scale are the preferred method for assessing pain and behavioural scales should only be used when severe cognitive impairment is present.  Collectively the tools reviewed corroborate the use of common behavioral indicators of pain, but don't demonstrate sufficient evidence for the use of a particular scale.  Need to focus on scales that already exist and spend more time validating them. Most promising scales for practice and research: the PACSLAC, Abbey Pain Scale, and DOLOPLUS-2. |
| [44] | 13 tools: Abbey Pain Scale; ADD; Behavior checklist; CNPI; Doloplus-2; DS-DAT;FACS; NOPAIN; PACSLAC; PADE; PAINAD; PATCOA; PBM | PAINAD seems best feasible scale for practice. Suggests need to develop cut off scores for scales to indicate whether or not to provide interventions for pain - need to link assessment with treatment algorithms. |
| [21] | 12 tools: Abbey Pain Scale; CNPI;DOLOPLUS2;ECPA; ECS; NOPPAIN; Observational Pain Behavior Tool; PACSLAC; PADE; Pain Assessment Tool for Use with Cognitive Impaired Adults;  PAINAD; RaPID | None of these assessment scales is convincingly the most appropriate, and therefore preferable, scale for assessing pain in elderly people with dementia. PAINAD, PACSLAC, DOLOPLUS2 and ECPA show the best (but moderate) psychometric qualities (none scored more than 12 points out of 20) among those reviewed.  For implementation of one of these tools in clinical practice, two further criteria were added: scale items specifically geared towards elderly persons with dementia and most comprehensively tested in clinical settings. After adding these criteria to the psychometric properties, PACSLAC and DOLOPLUS2 appear the most appropriate scales among those currently available.  Recommendations for further research:  1. Further testing in clinical practice is needed. 2. Prevent excessive growth of newly developed tools. Further psychometric evaluation of existing scales should be given priority over developing new scales for future use.  3. Research to determine the utility, validity and reliability of pain assessment using a pain scale that takes the type of dementia into account.  4. Research to address the effect of cultural background on pain.  5. The relation between pain and behavioural and psychological symptoms of dementia needs to be explored. Research to determine sensitivity in relation to these other concepts, as well as the way pain affects these symptoms and how these symptoms affect pain expression. |
